# Nursing roles, competencies, and education in precision oncology: a scoping review

**DOI:** 10.1016/j.eclinm.2026.104080

**Published:** 2026-07-21

**Authors:** Amanda Drury, Sarah Sheehan, Alicia Cunningham, Memnun Seven, Fiona Hurley, Paula Flanagan, Mary Tanay, Kristen R. Haase, Sara Colomer Lahiguera, Maura Dowling

**Affiliations:** a School of Nursing and Midwifery, Trinity College of Dublin, University of Dublin, Dublin, Ireland; b Trinity Centre for Practice & Healthcare Innovation, School of Nursing and Midwifery, Trinity College Dublin, Ireland; c School of Nursing, Psychotherapy and Community Health, Dublin City University, Ireland; d Elaine Marieb College of Nursing, University of Massachusetts Amherst, MA, USA; e Berkshire Cancer Centre, Royal Berkshire NHS Foundation Trust, Reading, United Kingdom; f School of Nursing, Faculty of Applied Science, University of British Columbia, Vancouver, Canada; g Institute of Higher Education and Research in Health Care (IUFRS), University of Lausanne (UNIL), and Department of Oncology, Lausanne University Hospital (CHUV), Lausanne, Switzerland; h School of Nursing and Midwifery, University of Galway, Ireland

**Keywords:** Precision oncology, Genetics, Genomics, Nursing roles, Nursing competencies, Nursing education, Advanced practice nursing, Mainstreaming

## Abstract

**Background:**

Nurses contribute across the precision cancer care pathway, providing continuous, person-centred care and supporting genomic testing integration. However, the scope of nursing competencies required for precision cancer care and the extent to which current education prepares nurses for these roles remain unclear. This scoping review maps and synthesises evidence on nurses’ roles and competencies in precision cancer care, and educational provision in this field.

**Methods:**

A JBI scoping review was conducted; MEDLINE, CINAHL, PsycINFO, Embase and Scopus were systematically searched on July 18th 2025. International postgraduate and continuing professional education programmes relevant to nurses were identified via a generative artificial intelligence grey literature search undertaken between July 25th and July 30th 2025. Data were synthesised using convergent integrated thematic synthesis.

**Findings:**

Fifty-two publications were included, addressing nursing roles and/or competencies (*n* = 44) and educational interventions (*n* = 23). Sixty-five education programmes were identified. Five themes captured nursing roles: 1) genomic risk assessment and stratification; 2) genomic communication and shared decision-making; 3) precision cancer care pathway coordination and clinical navigation; 4) interprofessional collaboration and genomic service integration; and 5) professional governance, quality assurance, and advanced practice roles. Educational provision predominantly addressed foundational competencies, with less explicit preparation for roles requiring professional autonomy, interpretive expertise, leadership, and governance.

**Interpretation:**

Alignment between nurses’ competency expectations and educational preparation is uneven. We propose a three-level model of precision cancer nursing practice to differentiate foundational capabilities from advanced practice roles, distinguishing precision-informed, enhanced, and specialist precision cancer nursing practice. Explicit mapping of postgraduate curricula to stratified competency levels may support safe expansion of nursing scope within mainstreamed genomic cancer care.

**Funding:**

This research was supported with funding from the 10.13039/501100001638Dublin City University Undergraduate Summer Research Internship 2025, which supported AC’s time on the project. The authors alone are responsible for the content of this presentation.

**Research in context**

**Evidence before this study**

Precision medicine and genomics are increasingly integrated into cancer care, with nurses recognised as important contributors across the care pathway. Before undertaking this review, we searched MEDLINE, CINAHL, PsycINFO, Embase, and Scopus for published studies examining nursing roles, competencies, and education in precision medicine, cancer genomics, and related fields. Search terms included combinations of “precision medicine”, “genomics”, “cancer”, and “nursing”. Eligible studies included empirical research, reviews, guidelines, and educational reports without geographic restriction. Existing evidence was heterogeneous and largely descriptive, with variable quality and limited synthesis. Prior studies focused predominantly on genomic literacy, confidence, and educational interventions, with less attention to applied nursing roles, competency stratification, governance responsibilities, and alignment between education and practice. No previous synthesis was identified that integrated nursing roles, competencies, and educational preparation within precision cancer care pathways.

**Added value of this study**

This study provides a comprehensive synthesis of nursing roles, competencies, and educational provision in precision cancer care. It integrates evidence from peer-reviewed and grey literature, including international postgraduate and continuing education programmes. The review identifies five core domains of nursing contribution; 1) genomic risk assessment and stratification; 2) genomic communication and shared decision making; 3) precision cancer care pathway coordination and clinical navigation; 4) interprofessional collaboration and genomic service integration; and 5) professional governance, quality assurance, and advanced practice roles. The review also demonstrates a mismatch between expanding role expectations and predominantly foundational educational provision, particularly for responsibilities requiring greater autonomy, interpretive expertise, leadership, governance, and escalated decision-making. Building on these findings, the study proposes a three-level model of precision cancer nursing practice, distinguishing precision-informed, enhanced, and specialist precision cancer nursing practice, and explicitly linking competencies to scope of practice and professional accountability.

**Implications of all the available evidence**

Available evidence indicates that nursing roles are increasingly central to the implementation of precision cancer care but are not consistently supported by education and training for roles requiring advanced autonomy, interpretation, leadership, and pathway governance. Aligning education with clearly defined and stratified competencies is important to support safe role expansion within mainstreamed genomic cancer care. The proposed model provides a framework to guide curriculum development, workforce planning, governance structures, and service design. Future research should evaluate implementation of tiered education models, assess competency attainment, and examine how regulatory and service contexts enable or constrain nursing scope, accountability, and integration within precision cancer care systems.

## Introduction

Precision cancer care is now embedded within contemporary cancer care, with tumour sequencing, biomarker testing, and targeted therapies informing diagnosis, treatment, and surveillance strategies across tumour types.^1^ As clinical genomics becomes integrated into routine oncology pathways, responsibility for initiating, coordinating, and communicating testing increasingly extends beyond specialist genetics services to multidisciplinary cancer care teams.^2^, ^3^, ^4^, ^5^ As an integral part of the multidisciplinary team, nurses are central to prevention, screening, treatment delivery, survivorship, and palliative care in oncology.^6^ These evolving developments have implications not only for oncologists and laboratory professionals, but also for nurses, who provide continuous, person-centred care across the cancer continuum.

Precision cancer care increasingly relies on multidisciplinary teams in which genomic expertise is distributed safely across professional roles rather than confined to specialist genetics services. Genetic counselling is central to this landscape and can be understood both as a recognised profession, with specific education, credentialing, and regulatory requirements, and as a clinical process involving genomic risk assessment, informed decision-making, communication of genetic and genomic information, psychosocial support, and facilitation of family communication.^7^ In mainstreamed oncology pathways, selected elements of this process are increasingly undertaken by non-genetics professionals, including nurses, within defined scopes of practice. This does not imply equivalence between nursing roles and the professional role of genetic counsellors; rather, it reflects the need for competency-based workforce models that clarify shared and profession-specific responsibilities across the precision oncology workforce.^8^, ^9^, ^10^, ^11^

Professional organisations have recognised genomics as foundational to nursing practice and have developed competency frameworks to guide integration across levels of responsibility.^9^^,^^10^^,^^12^ At the same time, precision health agendas are broadening to include risk stratification, longitudinal surveillance, data integration, and system redesign, further expanding the potential scope of nursing practice.^13^^,^^14^ However, the pace of scientific and technological developments has raised questions regarding workforce readiness, role clarity, and the adequacy of existing educational pathways. Within precision cancer care, nurses may identify patients and families who could benefit from genomic assessment, supporting informed decision-making, explain possible testing outcomes and implications, coordinate testing pathways, reinforce results-related information, and facilitate referral to specialist genetics services when complexity exceeds their scope or competence.^4^^,^^15^^,^^16^ Clarifying how nursing roles intersect with, adopt elements of, or differ from genetic counselling practice is therefore essential for safe and equitable implementation of precision cancer care.

Nursing competence and scope of practice progress with preparation, clinical experience, professional authority, and accountability.^17^ Distinctions between generalist, specialist, and advanced levels of nursing practice are well established within the nursing literature and are commonly used to differentiate increasing complexity of clinical decision-making, autonomy, leadership, and responsibility for service development and evaluation.^17^ Specialist nursing roles are typically characterised by higher levels of expertise within a defined field of practice, whereas advanced practice roles encompass expanded autonomy, complex decision-making, systems leadership, and accountability for service improvement and governance.^18^ Competency expectations similarly progress across registered nurse, specialist nurse, and advanced practice nurse roles, reflecting increasing responsibility for coordination, interpretation, clinical judgement, interprofessional leadership, and pathway governance.^19^ In this review, we therefore distinguish between foundational competencies expected of the wider registered nursing workforce, specialist competencies associated with nurses working in specialist cancer settings, and advanced practice competencies requiring greater autonomy, interpretive responsibility, leadership, and governance.^17^^,^^18^ This distinction is important because safe integration of genomics into nursing practice depends on alignment between competence, role expectations, and professional accountability.^18^

Despite growing attention to genomic literacy among nurses, evidence suggests variability in confidence and preparedness for applied genomic decision-making, particularly in interpreting and communicating complex results.^20^ Education in precision medicine is expanding internationally, including postgraduate and interdisciplinary programmes delivered across biomedical, technological, and clinical faculties.^21^ Yet the extent to which these initiatives address nurses’ discipline-specific scope of practice, specialist competencies, and progression to advanced roles in cancer care remains unclear. To date, no comprehensive synthesis has mapped the roles that nurses currently undertake or are expected to undertake in precision cancer care, alongside the competencies and educational strategies intended to support these roles. In the absence of such synthesis, it is difficult to determine whether education, policy, and service development are aligned with emerging clinical realities. Therefore, this scoping review aims to map and synthesise existing evidence on nurses’ roles and competencies in precision cancer care, identify educational strategies designed to prepare nurses for these roles, and propose a stratified competency framework to support safe role operationalisation.

## Methods

### Design

This scoping review was conducted in accordance with the Joanna Briggs Institute (JBI) methodology for scoping reviews.^22^ A scoping approach was selected to accommodate heterogeneity in evidence and to map the breadth and nature of evidence on nurses’ roles, competencies, and education in precision medicine and cancer care. Scoping reviews are recommended for complex and heterogeneous literature, concept clarification, and descriptive mapping of emerging fields.^23^, ^24^, ^25^ Given the inclusion of qualitative, quantitative, mixed methods, and descriptive sources, a convergent integrated approach was used to synthesise evidence across methodologies.^26^ The authors developed a review protocol to guide the review process; however, it was not prospectively registered.

### Eligibility criteria

Eligibility criteria were defined using the Population, Concept, and Context (PCC) framework. Publications were included if they focused on registered nurses, advanced practice nurses, nurse educators, or nursing students involved in, or being prepared for, roles in precision cancer care, cancer genomics, or related fields (including genetics, omics, and personalised health). Eligible sources described nursing roles, scope of practice, competencies, and/or education and training initiatives, including curriculum content, delivery approaches, and reported outcomes. Clinical, academic, and policy contexts were eligible with no geographic restrictions. Publications were limited to those published from 2015 onwards to reflect the rapid expansion of precision oncology, mainstream genomic testing pathways, and contemporary genomics competency and education frameworks within cancer care.^1^^,^^20^^,^^21^ Eligible sources included primary research, reviews, narrative reports, expert commentaries, and relevant practice guidelines. Publications were excluded if they did not focus on nursing roles or did not involve nurses directly, or consisted solely of editorials, blogs, or anecdotal opinion pieces without substantive data or evidence-based discussion. Studies focused exclusively on medical or allied health roles without nursing relevance were also excluded.

### Search strategy

The search strategy was developed using the PCC framework. MEDLINE, CINAHL, PsycINFO, Embase and Scopus were searched on July 18th, 2025, combining MeSH terms and keywords relating to precision medicine (e.g., “precision medicine”, “personalised medicine”, “genomics”, “omics”, “genetics”, “precision cancer care”, “cancer genomics”) and nursing (e.g., “nurse”, “nurses”, “nursing”) (Appendix 1). Preliminary scoping searches indicated that inclusion of the terms “genetic counselling/counseling” retrieved a substantial body of literature focused primarily on the professional role of genetic counsellors rather than nursing practice and were therefore excluded to maintain specificity to the review objectives. To manage the volume of results and improve specificity, searches were restricted to the title and abstract fields.

A preliminary grey literature search using conventional web-based methods, including Google Advanced Search, was initially undertaken to identify postgraduate and CPD education programmes in precision oncology/genomics accessible to nurses across global regions. However, these searches generated a high volume of non-specific, duplicate, and non-relevant results, including programmes unrelated to nursing or precision cancer care, which limited feasibility and reproducibility. To support broader international mapping and improve identification of potentially relevant programmes across multiple countries and languages, a structured generative AI-assisted search approach was subsequently undertaken using a custom large language model (LLM), configured with Deep Research mode enabled in ChatGPT v4.2 (Appendix 2).

Searches were undertaken between July 25th, and July 30th 2025, and were run sequentially by global region (Europe, Africa, Asia, North America, South America, Oceania), using a standardised prompt protocol specifying scope, inclusion/exclusion criteria, and predefined Boolean search strings (e.g., precision medicine/genomics/omics AND nurse/nursing AND country name). Outputs were returned as structured country tables (programme title, country, institution, qualification, notes). Following initial mapping, the candidate programme list was re-submitted to the custom LLM for cross-checking against additional web sources. All candidate programmes identified through the AI-assisted searches were cross-checked against the preliminary conventional searches and manually verified against institutional webpages for eligibility and data extraction. Two authors (AD, AC) independently validated all included programmes against the original source programme webpages. Only programmes active during the original (August 2025) and updated (January 2026) searches were included; discontinued, duplicate, non-relevant, or programmes not specifically recruiting nurses were excluded.

### Study selection

All search results were imported into Covidence for deduplication and screening. Title and abstract screening were conducted independently by two reviewers (AD; AC). Full-text articles were retrieved for all records that met the inclusion criteria or for which eligibility was unclear. Full-text screening was performed independently by the same reviewers, with disagreements resolved through discussion. Grey literature search results were collated into a structured Excel database and independently screened by two reviewers (AD; AC). The final list of included programmes was cross-checked by a third author (SS). Records were included only where eligibility could be confirmed from publicly accessible institutional webpages; programmes presented in languages other than English were translated using the Google translate webpage function. Consensus on inclusion was achieved through discussion.

### Data extraction and charting

Data extraction was undertaken using a structured data extraction tool in Excel, which was developed and piloted on a subset of included studies. Two reviewers (AD; AC) independently extracted data, with discrepancies resolved through discussion. Extracted data from peer-reviewed publications included publication characteristics, study design, setting, population, descriptions of nursing roles and scope of practice, and reported knowledge, skills, competencies, or educational content relevant to precision medicine and oncology. Data from grey literature sources included programme identification (country, continent, institution, URL), institutional characteristics and accreditation status, qualification level and programme type, delivery mode, duration, credits, and award on completion. Detailed educational characteristics were recorded, including target audience, entry requirements, learning outcomes, curriculum content, and competency frameworks referenced. While the full data extraction dataset retained source identifiers (including programme titles, institutions, and URLs), all programmes were deidentified for publication to minimise institutional bias and ensure consistent reporting.

### Data analysis

Data were analysed by two reviewers (AD; MD) using a convergent integrated approach to synthesis consistent with JBI guidance for mixed methods reviews.^26^ All included evidence sources, including qualitative, quantitative, mixed methods, and grey literature publications, were imported into NVivo for manual coding and analysed within a single integrated analytic framework. An initial deductive coding phase was undertaken using three broad analytical categories aligned with the review objectives; nursing roles, competencies, and education programmes. Within each category, inductive thematic analysis was subsequently conducted to identify recurrent concepts, patterns, and role functions emerging from the data.^27^ Findings from quantitative studies were managed qualitatively through narrative interpretation to integrate them directly with qualitative and mixed methods evidence. Coding was conducted iteratively over multiple rounds, with ongoing comparison and refinement of codes and categories. In the later stages of analysis, related codes across the three analytical categories were consolidated to identify overarching thematic groupings connecting nursing roles, competencies, and educational preparation. This process enabled evidence derived from different methodologies to contribute jointly to integrated themes rather than being synthesised separately. Education programme literature and grey literature sources were analysed using the same coding and thematic procedures as peer-reviewed studies to ensure consistency across evidence types.

Competencies were further interpreted with reference to published competency frameworks and standards.^9^^,^^10^^,^^12^ Competency statements were aligned to each theme and stratified by practice level; foundational, specialist, or advanced, based on role descriptions, published competency frameworks, and the degree of autonomy, decision-making complexity, and governance responsibility described in the literature.^17^, ^18^, ^19^ This approach enabled transparent linkage between reported nursing roles and the competencies needed to support practice across levels of responsibility.

### Role of the funding source

The funder of the study had no role in study design, data retrieval, data extraction, data analysis, data interpretation, or writing of the report.

## Results

### Characteristics of included data sources

**Fig. 1:**
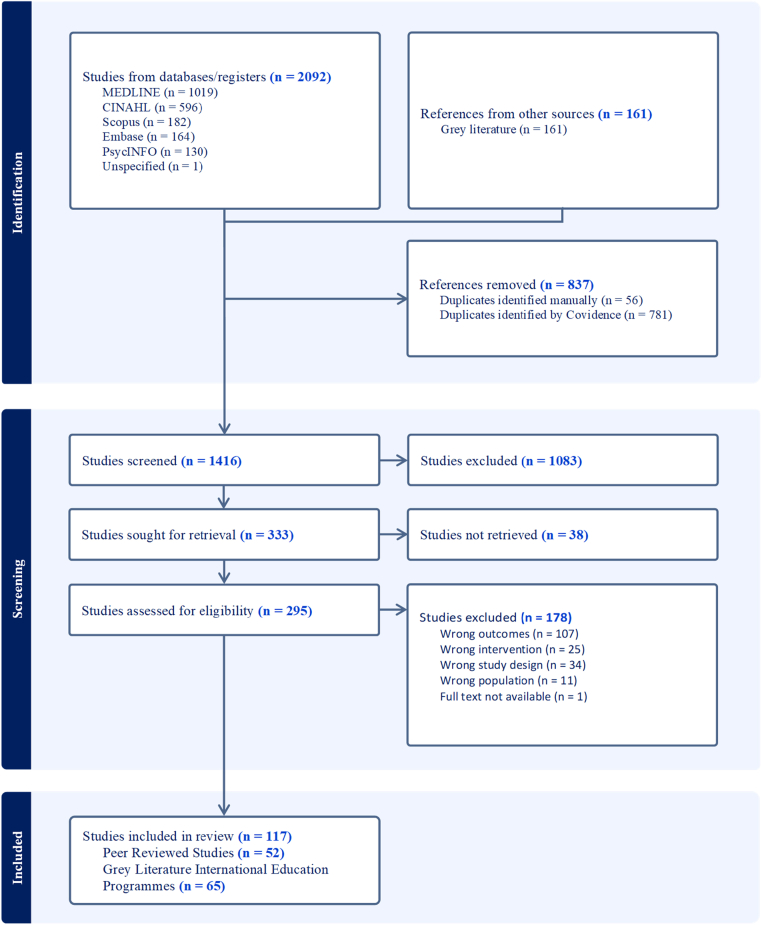
PRISMA flow chart.

In the peer-reviewed publications, initial searches yielded 2092 publications, of which 52 were retained following screening (Fig. 1). Of these, 44 reported results on nursing roles (Appendix 3) or competencies in precision cancer care (Appendix 4), and 23 publications reported on the education and training of nurses (Appendix 5). Most peer-reviewed publications originated from the United States (n = 23), followed by multi-country studies (n = 10). Fewer studies were identified from the United Kingdom (n = 4), Italy (n = 3), and the Netherlands (n = 2), with single studies from Australia, Brazil, China, Ireland, Nigeria, Saudi Arabia, South Africa, Spain, Switzerland, and Türkiye. The peer-reviewed literature comprised a heterogeneous body of evidence, most commonly quantitative studies (n = 14), discussion papers (n = 11), and case studies (n = 8). Smaller numbers of mixed methods studies (n = 5), narrative literature reviews (n = 4), clinical practice guidelines (n = 2), documentary analyses (n = 2), quasi-experimental studies (n = 2), and individual qualitative, quality improvement, scoping, and systematic review papers were also included.

In the grey literature search, 161 programmes were identified in the initial searches, of which 65 were current and relevant to nurses working in precision cancer care (Appendix 6). Education programmes were geographically diverse but unevenly distributed. Most programmes were located in the United States (n = 13) and United Kingdom (n = 9), followed by Nigeria (n = 5), and Brazil, India, and South Africa (n = 4 each), with smaller numbers identified across Europe, Asia, Oceania, the Middle East, and South America. Online delivery was the most common mode of delivery (n = 21), followed by in-person (n = 19) and blended formats (n = 17); mode of delivery was not specified for eight programmes. Across regions, most postgraduate precision medicine and genomics programmes were delivered through medical schools, biomedical science, biotechnology institutes, or faculties of pharmacy and health sciences, with only six delivered in schools of nursing or explicitly designed for nurses. Most programs were delivered at postgraduate level, predominantly as MSc programs (n = 19), graduate certificates (n = 8), postgraduate certificates (n = 6), and postgraduate diplomas (n = 4), with a smaller number comprising short course, continuing professional development, certificate, and modular training formats. Accreditation or professional recognition was not specified for most programmes (n = 56). Among the few that reported accreditation or recognition, the approaches were heterogeneous, including university, professional society, continuing professional development, and jurisdiction-specific approvals, with limited consistency in how standards were described.

Nursing roles, competencies, and education were organised into five overarching themes; Genomic Risk Assessment and Stratification; Genomic Communication and Shared Decision-Making; Precision Cancer Care Pathway Coordination and Clinical Navigation; Interprofessional Collaboration and Genomic Service Integration; and Professional Governance, Quality Assurance, and Advanced Practice Roles. Table 1, Table 2, Table 3, Table 4, Table 5 summarise the competencies generated from the analysis thematically.

**Table 1:** Nurses’ scope of practice and competencies (basic, specialist and advanced) in genomic risk assessment and identification of eligible patients.

Subtheme	Role/scope of practice	Basic competencies (Graduate/entry-level Registered Nurse)	Specialist competencies (Clinical Nurse Specialist/Oncology Nurse)	Advanced competencies (Advanced Practice Nurse/Nurse Practitioner/Advanced Nurse Practitioner)
1.1 Comprehensive risk assessment data capture	Conduct systematic assessment to identify individuals at increased hereditary or molecular cancer risk Integrate genomic considerations into routine nursing assessment	• Collect comprehensive personal health history including cancer diagnoses, age of onset, comorbidities.^16^^,^^28^ • Obtain family history including first- and second-degree relatives.^29^, ^30^, ^31^ • Recognise basic hereditary cancer “red flags” (e.g., early onset, multiple primaries).^32^	• Integrate personal, family, and clinical data to identify patients who may benefit from genetic/genomic testing.^15^^,^^16^^,^^33^^,^^34^ • Conduct risk assessment across modalities (face-to-face, telephone, virtual).^33^^,^^35^^,^^36^ • Prioritise assessment data to support timely referral and testing.^37^	• Perform holistic genomic risk assessment incorporating comorbidities, psychosocial context, and social determinants of health.^13^^,^^38^ • Evaluate appropriateness and timing of genomic testing within prevention, diagnostic, or treatment pathways.^20^^,^^39^
1.2 Pedigree construction, documentation, and longitudinal updating	Generate, document, and maintain pedigree-based risk information to support hereditary cancer risk stratification	• Construct and document a basic three-generation pedigree using standard symbols.^29^^,^^30^^,^^40^ • Record cancer type and age at diagnosis for relatives.^16^	• Analyse pedigree patterns to identify possible inherited cancer syndromes.^41^^,^^42^ • Update pedigrees over time as family history or test results change.^16^^,^^43^ • Address pedigree limitations (small families, adoption, incomplete knowledge).^16^	• Interpret pedigree data alongside tumour testing and molecular findings to guide testing strategies.^42^^,^^43^ • Distinguish mixed hereditary and sporadic cancer patterns to refine risk assessment.^42^
1.3 Risk recognition and eligibility determination	Identify patients eligible for genetic counselling, testing, or enhanced surveillance Activate referral or testing pathways	• Screen patients for potential eligibility based on family and personal history.^28^^,^^29^^,^^44^ • Provide initial information about referral and testing processes.^41^	• Apply guideline-based criteria and structured screening tools to determine eligibility.^33^^,^^39^^,^^45^^,^^46^ • Facilitate referrals to genetics services and communicate risk concerns to MDTs.^4^^,^^31^^,^^32^ • Triage demand to reduce inappropriate referrals.^35^	• Determine test appropriateness, including panel selection and limitations, in collaboration with genetics professionals.^42^^,^^47^ • Lead shared decision-making related to testing and surveillance eligibility.^13^^,^^36^ • Oversee longitudinal risk stratification and surveillance for individuals with actionable variants.^39^
1.4 Testing pathway linkage and risk-management planning	Link identified risk to appropriate testing, counselling, and surveillance pathways Support integration of genomic information into care planning	• Document assessment findings to support continuity of care.^28^ • Facilitate referrals to genetics or oncology services as indicated.^15^^,^^29^	• Coordinate pre-test workflows including consent preparation and logistics.^5^^,^^36^^,^^44^ • Track testing status and ensure results are available to inform care decisions.^48^ • Support informed decision-making related to testing pathways.^11^	• Design, implement, and evaluate genomic screening and testing pathways.^39^^,^^42^ • Integrate genomic findings into personalised risk-management and surveillance plans.^13^^,^^34^ • Address system-level barriers to equitable access to genomic services.^38^^,^^42^

**Table 2:** Nurses’ scope of practice, and competencies (basic, specialist and advanced) in genomic communication and shared decision-making.

Subtheme	Role/scope of practice	**Basic** c**ompetencies (Graduate/Entry-level Registered Nurse)**	**Specialist** c**ompetencies (Clinical Nurse Specialist/Oncology Nurse)**	**Advanced** c**ompetencies (Advanced Practice Nurse/Nurse Practitioner/Advanced Nurse Practitioner)**
2.1 Enabling informed decision-making	Support patients to make informed, voluntary decisions about genetic/genomic testing	• Explain the purpose of genetic/genomic testing in clear, non-technical language.^11^^,^^28^ • Describe potential benefits, risks, and limitations of testing.^38^^,^^42^ • Reinforce that counselling does not obligate testing and support voluntariness.^32^ • Recognise when patient understanding or decisional readiness is limited and escalate appropriately.^29^^,^^47^	• Conduct structured consent discussions for germline testing embedded within oncology pathways.^4^^,^^5^^,^^44^ • Assess patient baseline knowledge, emotional responses, and decisional needs during consent processes.^15^^,^^16^^,^^34^^,^^49^ • Address ethical, legal, psychosocial, and familial implications of testing.^15^^,^^16^ • Escalate to genetics professionals when test complexity or uncertainty exceeds scope.^41^^,^^43^	• Serve as first-contact professional providing genomics-informed counselling and decision support within defined pathways.^13^ • Assure informed consent for complex or multigene testing, including discussion of uncertainty and downstream implications.^50^^,^^51^ • Apply advanced ethical reasoning to genomic decision-making, including balancing patient autonomy and family implications.^13^ • Lead governance-aligned consent processes across services and pathways.^20^
2.2 Translating complex genomic information	Translate genetic and genomic information into patient- and family-understandable formats	• Assess patient and family baseline knowledge and informational needs.^15^^,^^30^ • Distinguish genetic susceptibility from clinical diagnosis.^37^ • Use clear, culturally and linguistically appropriate communication strategies.^47^^,^^52^ • Provide credible, up-to-date educational resources.^15^^,^^29^	• Provide personalised genetic education tailored to health literacy and learning style.^28^^,^^31^^,^^37^^,^^44^^,^^53^ • Prepare patients for uncertain outcomes, including variants of uncertain significance and limitations of multigene panels.^16^^,^^34^^,^^41^ • Clarify practical concerns (e.g., insurance, cost, discrimination fears) related to testing.^53^ • Design and evaluate educational materials for readability and comprehension.^42^	• Communicate complex genomic concepts in shared decision-making about treatment and prevention.^13^ • Provide genomics-informed counselling and decisional support on benefits, risks, and limitations of testing and interpretation.^54^ • Integrate somatic and germline findings into patient-centred discussions.^50^ • Lead education initiatives addressing misinformation and direct-to-consumer testing risks.^13^
2.3 Extending communication beyond the patient	Support family communication, cascade testing implications, and longitudinal understanding of genomic risk	• Encourage discussion of genetic risk and results with family members.^30^^,^^32^ • Provide basic guidance on why family history and communication matter.^28^ • Offer emotional support alongside information provision.^35^ • Reinforce health promotion and prevention messages based on genetic risk.^52^	• Guide patients through family disclosure and cascade testing implications.^34^^,^^42^^,^^50^ • Assess barriers to family communication and provide anticipatory guidance.^32^^,^^42^ • Support patients when results are negative or uninformative, maintain risk awareness.^36^^,^^50^ • Facilitate patient support groups combining education and peer discussion.^4^	• Coordinate complex family-based care and cascade testing pathways.^43^ • Provide longitudinal counselling for risk-reducing decisions (e.g., surveillance, surgery).^50^ • Navigate ethical tensions related to disclosure, privacy, and family dynamics.^13^ • Lead development of innovative or digital tools for family communication and education.^50^

**Table 3:** Nurses’ scope of practice, and competencies (basic, specialist and advanced) in precision cancer care pathway coordination and clinical navigation.

Subtheme	Role/scope of practice	**Basic** c**ompetencies (Graduate/Entry-level Registered Nurse)**	**Specialist** c**ompetencies (Clinical Nurse Specialist/Oncology Nurse)**	**Advanced** c**ompetencies (Advanced Practice Nurse/Nurse Practitioner/Advanced Nurse Practitioner)**
3.1 Pathway logistics for precision diagnostics and research infrastructure	Coordinate genomic and biomarker testing workflows to ensure timely, guideline-concordant diagnostics Track test ordering, turnaround times, results, and tissue adequacy across laboratories Conduct telephone or pre-clinic triage to collect histories, documentation, and assess readiness for genetic evaluation Support consent processes and specimen coordination for biobanking and research infrastructure	• Identify patients who may benefit from genomic or biomarker testing based on assessment and family history.^15^^,^^29^ • Explain purpose and process of testing in clear, patient-appropriate language.^15^ • Document testing status and facilitate referrals to genetics services when indicated.^15^	• Review pathology reports to identify molecular profiles and testing gaps.^48^ • Maintain logs/databases tracking molecular results, tissue availability, and testing timelines.^48^ • Conduct structured triage to prioritise appropriate referrals and manage service demand.^35^^,^^36^ • Coordinate consent timing, specimen collection, and documentation for research and biobanking.^49^	• Determine appropriateness and sequencing of additional or repeat molecular testing.^42^^,^^48^ • Authorise or order testing within scope and governance frameworks.^42^ • Design, evaluate, and oversee diagnostic pathways and precision testing infrastructure.^2^^,^^13^
3.2 Precision-informed care planning and treatment trajectories	Integrate genomic and biomarker results into individualised care planning and treatment coordination Align timing of clinical appointments with result availability to support decision-making Coordinate complex treatment pathways, including advanced therapies such as Chimeric Antigen Receptor T-cell therapy (CAR-T)	• Support care planning informed by genomic findings and patient preferences.^28^ • Educate patients and families about treatment processes and expectations.^37^ • Monitor responses and document outcomes across treatment phases.^28^	• Coordinate multidisciplinary care plans incorporating genomic eligibility criteria and treatment readiness.^4^^,^^55^ • Adapt care plans based on treatment trajectory, toxicity, and patient response.^28^^,^^34^ • Coordinate eligibility assessments and pre-treatment requirements for advanced therapies.^56^	• Lead precision-informed treatment decision-making and longitudinal surveillance.^13^^,^^39^ • Oversee coordination of highly complex therapies (e.g., CAR-T), including escalation pathways and long-term follow-up.^56^^,^^57^
3.3 Family-risk management and uncertainty	Coordinate follow-up for patients and families after germline findings Support cascade testing pathways and referrals for at-risk relatives Manage communication and follow-up related to variants of uncertain significance	• Prepare patients for uncertainty and limitations of testing.^15^^,^^41^ • Provide psychosocial support alongside genomic information.^15^ • Facilitate referrals to genetics professionals when interpretation exceeds competence.^43^^,^^45^	• Coordinate cascade testing logistics, family letters, and educational materials.^4^^,^^16^^,^^34^ • Support patients and families when results are negative or uninformative.^36^^,^^50^ • Monitor follow-up and re-engagement when variant classifications change.^43^	• Interpret and manage complex genomic results, including variants of uncertain significance reclassification, in collaboration with genetics professionals.^43^ • Coordinate multi-service or cross-jurisdictional cascade testing pathways.^43^ • Determine when ongoing management vs. referral is appropriate based on scope and competence.^43^
3.4 Pharmacogenomics and precision medication delivery within navigation	Integrate pharmacogenomic considerations into medication administration and monitoring Educate patients and families about genomics-influenced medication responses	• Obtain family and medication history relevant to pharmacogenomic risk.^29^ • Observe, document, and report adverse drug reactions influenced by genomic variation.^58^	• Educate patients on pharmacogenomic implications for dosing and side-effect monitoring.^58^ • Tailor symptom management and monitoring plans based on genomic risk.^15^	• Integrate pharmacogenomic data into prescribing and treatment decisions within scope.^51^^,^^58^ • Lead precision medication management strategies across services.^13^

**Table 4:** Nurses’ scope of practice, and competencies (basic, specialist and advanced) in interprofessional collaboration and genomic service integration.

Subtheme	Role/scope of practice	**Basic** c**ompetencies (Graduate/Entry-level Registered Nurse)**	**Specialist** c**ompetencies (Clinical Nurse Specialist/Oncology Nurse)**	**Advanced** c**ompetencies (Advanced Practice Nurse/Nurse Practitioner/Advanced Nurse Practitioner)**
4.1 Positioning nurses within interprofessional genomic pathways	Participate as a member of multidisciplinary teams (MDTs) integrating genomic information into oncology care planning.^15^^,^^28^ Act as a communication conduit between patients, oncology teams, and genetics services	• Communicate personal and family history information to MDTs.^28^ • Collaborate with oncology and genetics professionals to support genomic care delivery.^11^^,^^29^^,^^34^ • Recognise when genomic expertise beyond nursing scope is required.^43^	• Act as a central point of contact for genomic information within MDTs.^4^ • Partner with genetic counsellors and oncologists to align care plans with current guidelines.^53^ • Integrate genomic risk information into interdisciplinary care discussions.^42^	• Lead interprofessional collaboration in precision cancer care services.^13^ • Influence MDT decision-making using genomic and molecular profiling data.^13^ • Oversee integration of precision health principles across services and settings.^13^
4.2 Referral decision-making and referral facilitation	Identify patients who may benefit from genetics services and facilitate access through referral pathways	• Recognise hereditary cancer red flags from personal and family history.^29^^,^^32^ • Provide basic information about genetic counselling and testing pathways.^41^ • Facilitate referral to credentialed genetics professionals.^15^^,^^31^	• Apply guideline-based eligibility criteria to identify appropriate referrals.^39^^,^^45^ • Initiate and coordinate referrals to genetics, psychology, surgical, and support services.^4^ • Communicate referral rationale and follow-up requirements across teams.^42^	• Determine appropriateness of genomic testing and referral in collaboration with genetics professionals.^39^^,^^42^ • Lead development and optimisation of referral pathways within oncology services.^13^ • Advocate for equitable access to genetics services at system level.^38^
4.3 Triage, stratification, and pathway governance	Allocate patients to appropriate genomic pathways based on risk, complexity, and service capacity	• Recognise limits of own genomic competence and seek escalation when required.^43^ • Support access to genetics services through appropriate referral routes.^20^	• Undertake telephone or service-based triage to assess eligibility and readiness.^35^^,^^36^ • Collect and synthesise family history and documentation for genetic assessment.^36^ • Participate in tandem or triage referral models with genetics professionals.^41^	• Oversee genomic diagnostic and referral pathways (e.g., Lynch syndrome, hereditary clinics).^2^ • Govern triage processes to balance access, appropriateness, and service sustainability.^13^ • Provide expert consultation and escalation pathways for complex cases.^43^
4.4 Mainstreaming and nurse-led genomic service delivery models	Deliver components of genetic testing pathways within oncology services under mainstreaming models	• Identify patients eligible for mainstreamed genetic testing using established criteria.^15^ • Support coordination of testing and referral within oncology workflows.^30^	• Provide pre-test education and obtain consent for germline testing in defined pathways.^4^^,^^5^ • Deliver selected test results and coordinate onward referral when indicated.^36^^,^^50^	• Lead nurse-led genetic clinics and mainstream testing programmes.^2^^,^^4^ • Assume accountability for pathway governance, data integrity, and guideline adherence.^59^ • Evaluate effectiveness, safety, and sustainability of nurse-led genomic service models.^13^

**Table 5:** Scope of practice, and competencies (basic, specialist and advanced) in professional governance, ethics, quality and capability development.

Subtheme	Role/scope of practice	**Basic** c**ompetencies (Graduate/Entry-level Registered Nurse)**	**Specialist** c**ompetencies (Clinical Nurse Specialist/Oncology Nurse)**	**Advanced** c**ompetencies (Advanced Practice Nurse/Nurse Practitioner/Advanced Nurse Practitioner)**
5.1 Continuing competence and professional development	Maintain and advance genomic competence in response to rapidly evolving evidence and technologies	• Recognise genomics as relevant to all areas of cancer nursing practice and acknowledge the need for ongoing learning.^28^ • Demonstrate basic genomic literacy (terminology, inheritance patterns, testing purposes).^30^^,^^37^ • Access credible, current genomic information resources to support practice.^54^ • Recognise that variant interpretation and recommendations may change over time.^16^	• Maintain up-to-date knowledge of cancers and conditions for which genomic testing is indicated.^20^ • Engage in genomics-focused continuing professional development activities.^53^^,^^60^ • Integrate emerging evidence into specialty oncology practice.^37^^,^^47^	• Maintain formal certification or advanced education in genetics/genomics.^42^^,^^43^ • Monitor regulatory and professional requirements relevant to advanced practice.^42^ • Update clinical guidelines and patient self-management resources as evidence evolves.^13^ • Engage in advanced learning related to AI and emerging precision technologies.^61^
5.2 Evidence-based practice, quality improvement, and evaluation	Apply evidence, evaluate practice, and contribute to quality improvement in precision cancer care services	• Use current evidence and guidelines to inform care.^28^ • Engage in reflective self-assessment of genomic competence.^15^ • Recognise uncertainty and limitations within genomic evidence.^47^	• Evaluate effectiveness of genomic interventions on patient outcomes.^15^ • Participate in audit and quality improvement activities related to genomic care.^28^ • Apply critical thinking in interpretation of genomic information in oncology practice.^47^	• Lead quality improvement and service evaluation initiatives in precision cancer care.^13^^,^^28^^,^^39^ • Integrate research findings into advanced clinical practice.^13^ • Disseminate genomic and precision cancer care knowledge through publications and presentations.^42^
5.3 Ethical-legal stewardship of genomic and artificial intelligence (AI) information	Uphold ethical principles, protect autonomy, and steward responsible use of genomic and AI-enabled information	• Identify ethical, legal, and social issues related to genomics.^29^^,^^30^ • Recognise factors that undermine informed and voluntary decision-making.^15^ • Reflect on personal values and potential biases.^30^^,^^52^	• Apply ethical principles to genomic decision-making in oncology contexts.^37^ • Provide culturally sensitive, non-judgmental care in genetic testing pathways.^28^ • Educate patients on benefits, risks, and limitations of testing.^41^	• Lead ethically responsive implementation of precision cancer care services.^13^ • Advocate for equitable access to genomic care.^38^ • Oversee ethical governance of AI-enabled decision support in oncology.^61^
5.4 Privacy, confidentiality, and information governance	Protect genetic information and ensure compliant data handling	• Maintain privacy when discussing genetic/genomic information.^30^ • Understand basic legal protections and limitations related to genetic data.^30^	• Handle genetic and family information responsibly within oncology services.^47^ • Maintain confidentiality for patients and families across care transitions.^37^	• Ensure ethical and lawful use of genomic and AI-derived data in clinical decision-making.^13^^,^^61^
5.5 Scope of practice, accountability, and role negotiation	Practise within scope, articulate boundaries, and ensure appropriate escalation and referral	• Articulate own scope and boundaries in genomics.^37^ • Use information technology to access credible genomic resources.^37^	• Recognise limits of expertise and refer appropriately to genetics professionals.^47^ • Apply genomics within defined oncology specialty scope.^62^	• Negotiate role responsibilities with interprofessional stakeholders.^42^ • Provide consultation and document genomic communication.^42^ • Assume leadership in professional practice, governance, and policy influence.^28^^,^^62^

#### Genomic risk assessment and stratification

Genomic risk identification and patient stratification are consistently described as foundational nursing contributions within precision cancer care, positioning nurses as early identifiers of patients and families who may benefit from genetic or genomic testing (Table 1). Across studies, nurses operate at the interface between routine clinical assessment and specialist genetics services, applying systematic approaches to recognise hereditary and genomically mediated risk factors in everyday oncology encounters.^16^^,^^28^^,^^32^

Role descriptions and competency sources align most closely with comprehensive risk assessment data. Nurses expand routine assessment to include personal and surgical history, reproductive and social factors, lifestyle and environmental exposures, and family cancer history to identify indicators of hereditary cancer syndromes, emphasising assessment beyond the presenting tumour site.^16^^,^^28^^,^^31^^,^^32^ Longitudinal relationships are described as enabling disclosure of sensitive family information and recognition of risk among populations historically under-referred for testing.^32^ These expectations map to basic and specialist competencies in comprehensive assessment, risk synthesis, and identification of candidates for genomic services.^15^^,^^37^

Pedigree construction is one of the most consistently reported nursing activities. It is reinforced in competency frameworks as spanning basic construction to more advanced interpretation, integrating tumour testing and differentiating hereditary from sporadic patterns.^16^^,^^29^^,^^31^^,^^41^^,^^42^^,^^47^ Several studies highlight the need for ongoing updates as diagnoses, test results, or variant reclassifications emerge, while acknowledging limitations of incomplete family histories.^16^^,^^43^ However, as roles extend into eligibility determination and pathway governance, alignment between roles and competencies becomes more variable; nurses flag risk factors and apply guideline-based criteria, and specialist/advanced roles include structured screening tools, triage, and multidisciplinary deliberation to manage ambiguity and demand.^28^^,^^32^^,^^35^^,^^39^^,^^45^ In mainstreamed models, nurses may independently assess eligibility and initiate testing with escalation for complex cases.^3^^,^^36^^,^^44^ However, multiple reports caution that multigene panels and variants of uncertain significance introduce uncertainty that requires advanced competencies not consistently evident in role descriptions.^3^^,^^34^^,^^36^^,^^41^^,^^43^

Education addressing this theme prioritises foundational genomic knowledge (e.g., genetics/genomics, inheritance, chromosomal variation, mechanisms of disease), which supports baseline competence but is often presented as pre-requisite knowledge, rather than explicitly as preparation for nursing-led risk assessment and stratification.^30^^,^^40^^,^^54^^,^^60^^,^^63^ Peer-reviewed education sources more clearly align with specialist competencies by operationalising family history and pedigree skills, and by applying eligibility determination for tumour or germline testing in oncology-focused and mainstreaming initiatives.^3^, ^4^, ^5^^,^^30^^,^^38^^,^^64^^,^^65^ Nonetheless, longitudinal pedigree updating and explicit preparation for nurses’ accountability in initiating and managing testing pathways remain under-specified, indicating that preparation for advanced practice expectations in these areas warrants further development (Appendix 6).

#### Genomic communication and shared decision-making

Communication of genomic and decisional support emerged as core relational nursing contributions within precision cancer care, centred on enabling understanding, voluntariness, and sustained engagement with genomic information across the patient-family unit (Table 2). Across studies, nurses are positioned as educators and advocates who translate technical genomic content into meaningful information for patients, reinforce patient autonomy, and sustain communication over time as testing decisions and results become available.^16^^,^^28^^,^^31^^,^^32^

Consent is consistently framed as an iterative process embedded within therapeutic relationships rather than a discrete event. Nurses provide time, structured information, and emotional support to facilitate decisions aligned with patient values and preferences.^5^^,^^16^^,^^28^^,^^34^ Nurses play a key role in ensuring informed consent and voluntariness, which are ethical cornerstones of decisional support.^32^ In mainstreaming pathways, specialist nurses and clinical nurse specialists increasingly deliver pre-test counselling and obtain consent for germline testing, particularly in BReast CAncer gene (BRCA)-related pathways.^4^^,^^5^^,^^50^ Nursing responsibilities also extend to research-facing consent (e.g., tissue procurement), including explanation of downstream use and patient rights.^49^ These activities align with competency expectations related to explaining purpose, benefits, risks, limitations, and escalation to specialist care when complexity exceeds scope.^11^^,^^15^^,^^41^^,^^43^^,^^47^

A defining competency described across the literature is translating complex genomic concepts into understandable formats and supporting interpretation for patients and families. Nurses help patients process information, prepare questions for other members of the healthcare team involved in their care, and clarify distinctions between counselling and testing and between somatic and germline findings.^30^, ^32^^,^^44^^,^^53^ Communication demands intensify with multigene testing, where nurses support anticipatory framing around uncertainty, including variants of uncertain significance, and reinforce that management may remain guided by personal and family history.^41^^,^^43^ Competency frameworks reinforce culturally responsive communication, use of credible resources, and explanation of risk and uncertainty.^15^^,^^37^^,^^52^

Communication roles consistently extend beyond the individual patient. Nurses encourage disclosure to relatives, support cascade implications, and reinforce ongoing risk management even when results are negative or uninformative.^34^^,^^36^^,^^42^^,^^50^ They are also described as longitudinal points of contact that provide informational and psycho-emotional support, including in genetics clinic settings.^35^ These functions map to competencies in family communication support and follow-up, with advanced roles encompassing complex family pathways and ethical issues.^13^^,^^34^^,^^43^

The education evidence partly aligns with these competency demands. Training described in peer-reviewed literature is most consistently aligned with specialist nursing roles; consent and counselling are explicitly operationalised in mainstreaming and hereditary cancer pathways, including structured mentoring and in-service training that support nurses in obtaining consent, counselling, and communicating results to patients.^4^^,^^5^ Practical training also addresses how to approach patients, explain questions, and evaluate understanding in cancer interactions.^37^^,^^45^ Advanced interpretive competencies are clearest in oncology genomics education, where modules focus on interpreting actionable findings, contextualising uncertainty, and recognising when escalation to specialist services or tumour boards is indicated.^3^^,^^65^ Psychosocial and family-centred decisional support is also explicitly included in peer-reviewed curricula, emphasising emotional consequences and communication with at-risk relatives.^3^^,^^64^^,^^66^

In contrast, education programmes identified in grey literature searches more often list communication, counselling, or genetic testing in programme descriptors; however, they do not clearly define nursing accountabilities, decision boundaries, or the applied competencies required for specialist and advanced roles (Appendix 6). This creates an implementation gap where roles and competency frameworks describe nurses as active agents in consent, results communication, and family-risk dialogue. At the same time, many grey literature programmes under-specify how learners are prepared to operationalise these skills in routine oncology encounters, particularly in mainstreamed models that require structured escalation and governance.

#### Precision cancer care pathway coordination and clinical navigation

Nursing roles within precision cancer care pathways extend beyond supportive care to encompass coordination of diagnostics, therapeutics, and longitudinal follow-up (Table 3). Nurses initiate and track molecular and biomarker testing, coordinate specimens, liaise with laboratories, and monitor tissue adequacy, repeat sampling, and turnaround times to prevent treatment delays.^36^^,^^48^ Triage models describe nurses synthesising documentation and coordinating access to genetics services.^35^^,^^36^ Research-facing roles, including biobanking coordination, further extend this pathway oversight, aligning nursing roles with specialist competencies in structured triage, consent processes, specimen collection, pathology report review, and service coordination.^35^^,^^48^^,^^49^

Supportive and navigational functions converge after genomic testing is initiated or results are returned. At a foundational level, graduate nurses reinforce understanding of testing status and implications, prepare patients for uncertainty and limitations, including uninformative findings, document outcomes, facilitate referral when interpretation exceeds competence, and provide psychosocial support alongside genomic information.^15^^,^^41^^,^^43^^,^^45^ They educate patients and families about treatment expectations, monitor responses across treatment phases, and observe and report adverse drug reactions influenced by genomic variation, thereby linking genomic insight to ongoing supportive care.^28^^,^^37^^,^^58^ At specialist level, this supportive role extends into genomics-informed care planning and advanced therapy pathways. Nurses tailor symptom management and monitoring plans based on genomic risk, coordinate appointments to align with result availability, support patients and families when findings are negative or uninformative, and oversee cascade testing logistics and family communication.^4^^,^^15^^,^^16^^,^^34^^,^^36^^,^^50^ They also monitor re-engagement when variant classifications change and integrate pharmacogenomic considerations into medication management and education, reinforcing continuity and safe escalation within defined competency boundaries.^15^^,^^29^^,^^43^^,^^58^

Education programmes, however, only partially align with these operational competencies. Peer-reviewed initiatives provide substantial coverage of targeted therapies, gene-drug interactions, and genomics-informed dosing,^30^^,^^38^^,^^63^ alongside detailed instruction in sequencing technologies, molecular pathology, and bioinformatics.^37^^,^^60^^,^^67^ These components support interpretive awareness and pharmacogenomic literacy but are seldom framed as coordination or navigation competencies. Specimen logistics and laboratory processes are addressed in some curricula,^60^ aligning with nursing involvement in sample handling, yet are not consistently linked to pathway governance. International programmes identified via the grey literature search similarly emphasise advanced diagnostics and therapeutics but rarely position nurses as pathway coordinators or leaders (Appendix 6). While technical knowledge is well represented across both evidence sources, explicit preparation for nurse-led workflow management, triage, turnaround monitoring, and longitudinal follow-up remains limited. Consequently, education appears better aligned with scientific understanding than with the complex coordination and service-delivery competencies required of specialist and advanced precision cancer care nurses.

#### Interprofessional collaboration and genomic service integration

Nurses’ interprofessional role is described as a core enabling mechanism for delivering genomic and precision cancer care, with nurses operating at the interface between oncology teams, genetics services, pathology/laboratory systems, and multidisciplinary decision-making (Table 4). Role descriptions emphasise coordination, bidirectional information transfer, and structured escalation to specialist genetics expertise, particularly as testing becomes embedded in routine oncology workflows.^4^^,^^28^ Within these models, nurses contribute to team-based synthesis of personal and family history information and support the incorporation of genomic considerations into care planning, indicating role expectations that extend beyond communication to active pathway integration.^15^^,^^28^^,^^34^

Competency sources broadly reinforce this positioning, specifying collaboration with interprofessional teams and use of organisational resources to deliver genomics-informed care.^11^^,^^29^^,^^37^ Evidence also demonstrates that interprofessional integration is not limited to oncology; in outpatient genetics management, oncology nurse practitioners collaborate with genetic counsellors to align risk management recommendations with screening guidance, reflecting specialist competence in shared clinical decision support across professional boundaries.^53^ However, the depth of operational accountability varies; while baseline expectations focus on collaboration and communication, specialist and advanced roles are more explicitly associated with referral facilitation, triage participation, and pathway oversight.^20^^,^^43^

Referral to specialist genetic services is described as central interprofessional functions. Nurses identify hereditary cancer risk indicators, provide preliminary information, and facilitate access to credentialed genetics professionals, often within structured pathways.^15^^,^^31^^,^^32^^,^^41^ Specialist roles extend to applying eligibility criteria and coordinating referrals to multiple services, including genetics and psychosocial supports.^4^^,^^45^ Triage models formalise this work through telephone-based triage and synthesis of pedigree and assessment documentation to manage demand and allocate genetics resources, reinforcing competencies in risk stratification, documentation, and escalation thresholds.^35^^,^^36^ Importantly, competency expectations frame escalation as a safety mechanism, recognising limits of competence and referring appropriately.^20^^,^^43^

Mainstreaming models further extend interprofessional expectations by locating components of genetic testing pathways within oncology services, with nurses providing pre-test education, consent, selected result communication, and onward referral for complex needs.^4^^,^^5^^,^^50^ Advanced practice roles are described as encompassing pathway governance and implementation of diagnostic pathways, including leadership of nurse-led clinics, aligning with higher-level competencies in service development and accountability.^2^ Nevertheless, concerns regarding counselling adequacy and result interpretation when genomics is decentralised underscore the need for clear delineation, supervision, and escalation in interprofessional models.^41^^,^^43^

Educational preparation does not consistently match these role and competency expectations. Peer-reviewed training more often includes explicit attention to genetics service structures, referral processes, and referral mechanisms, particularly where nurses engage directly with clinical genetics services.^4^^,^^30^^,^^66^ By contrast, education programmes identified in the grey literature search (Appendix 6) more commonly reference collaboration or referral at a descriptive level, with limited specification of interprofessional accountability, triage functions, or pathway governance for specialist and advanced nursing practice, creating a recurrent gap between service-model expectations and educational articulation.^38^^,^^66^

#### Professional governance, quality assurance, and advanced practice roles

Professional governance and capability development function as cross-cutting prerequisites for safe nursing practice in precision cancer care, underpinning nurses’ work in risk identification, genomic communication, pathway navigation, and interprofessional coordination (Table 5). This domain applies to all nurses, while specialist and advanced roles carry greater responsibility for stewardship, onward referral, and service oversight.^16^^,^^28^ Competency expectations consistently emphasise genomics as a rapidly evolving field, requiring ongoing updating of knowledge and skills to avoid inappropriate testing, misinterpretation, and preventable downstream uncertainty and cost.^43^ Baseline competence includes genomic literacy and the ability to access and use credible, current information sources to support safe practice.^30^^,^^37^^,^^54^ At specialist and advanced levels, the literature extends these expectations to maintaining current understanding of testing indications and clinical utility, and integrating new evidence into oncology care pathways and professional decision-making.^20^^,^^47^^,^^53^^,^^60^

Quality improvement and evaluation are repeatedly positioned as mechanisms through which nurses maintain safe and effective precision cancer care services. Nurses participate in audit and evaluation activities, while advanced practitioners are described as leading pathway evaluation and translating evidence into practice standards and service improvements.^13^^,^^15^^,^^28^^,^^39^^,^^58^ Equity considerations appear as quality-relevant risks, including distress associated with uncertainty and unequal access to genomic services, reinforcing that evaluation extends beyond technical performance to impacts across patient groups.^16^^,^^38^ Ethical stewardship is foregrounded throughout, including safeguarding voluntariness and informed decision-making, and recognising ethical, legal, and social implications such as privacy, confidentiality, cultural sensitivity, and responsible handling of familial information across care transitions.^15^^,^^28^, ^29^, ^30^^,^^37^^,^^47^^,^^52^ Emerging responsibilities include ethical governance of AI-enabled decision support and large datasets, indicating that specialist and advanced competence increasingly entails systems-level oversight.^13^^,^^61^ Scope clarity are repeatedly framed as governance competencies, with referral to genetics professionals positioned as a safety action when complexity exceeds competence or local resources.^34^^,^^42^^,^^43^^,^^62^

Education aligns most consistently with governance-related competencies, particularly ethical and legal stewardship. Peer-reviewed programmes commonly include consent, privacy, confidentiality, and cultural considerations, while advanced curricula more explicitly address leadership, professional role development, and the integration of genomics into practice, education, and research.^30^^,^^37^^,^^64^^,^^68^, ^69^, ^70^ Education addressing evidence-based practice and genomics-informed quality improvement appears in a smaller subset, including audit, hypothesis generation, and research translation.^38^^,^^60^ Among the education programmes identified via the grey literature search (Appendix 6), governance content is frequently present but often described generically (e.g., ethics, professional development) without specifying specialist or advanced accountability, evaluative responsibilities, or role negotiation. By contrast, peer-reviewed education more often articulates advanced stewardship functions, including leadership, evaluation, and research translation, suggesting that many grey literature programmes support baseline ethical literacy yet may under-prepare nurses for senior governance and quality roles central to specialist and advanced practice.

## Discussion

This scoping review demonstrates the breadth of nursing roles in precision cancer care across basic, specialist, and advanced levels of practice. Nurses are positioned as central actors in genomic risk identification, decisional support, pathway coordination, interprofessional collaboration, and professional governance, underscoring their integral role in implementing precision cancer care. However, while foundational genomic competencies are consistently supported within current education and training provision, preparation becomes less explicit and less robust as roles extend into areas that require greater autonomy, interpretive expertise, and leadership in pathway governance.

A prominent finding is the predominance of nursing roles as system-enablers within precision cancer care pathways. Nurses coordinate testing workflows, undertake triage and documentation, facilitate referrals, and provide longitudinal follow-up across diagnostic and treatment trajectories. These responsibilities align with mainstreaming models in which genomic testing is increasingly embedded within oncology services, rather than confined to specialist genetics teams.^2^, ^3^, ^4^, ^5^ At a systems level, such functions address recognised implementation challenges in precision medicine, including fragmented testing pathways, interpretive complexity, and inequities in access and reimbursement.^1^ Many of these system-enabling activities sit within baseline or early specialist competencies and can be supported through continuing professional development and workplace-based training. However, opportunities for role expansion are constrained by variability in how specialist and advanced nursing roles are defined and regulated internationally, as well as by uneven access to structured postgraduate preparation pathways.^1^^,^^6^^,^^50^

Many of the competencies identified in this review align with recognised components of the genetic counselling process, including genomic risk assessment, facilitation of informed decision-making, communication of genetic and genomic information, psychosocial support, and coordination of family-risk management pathways.^7^^,^^16^^,^^41^^,^^43^ In contemporary precision cancer care, these activities are increasingly distributed across multidisciplinary oncology teams through mainstreaming models that embed selected genomic practices within routine cancer services rather than limiting them to specialist genetics settings.^2^, ^3^, ^4^, ^5^ Within these models, nurses may undertake defined counselling-related activities, including structured risk assessment, pre-test education, consent processes, selected results communication, family-risk support, and referral facilitation, particularly where supported by protocols, additional education, and access to specialist escalation pathways.^4^^,^^36^^,^^50^^,^^53^ Importantly, within this review, the extent of nursing responsibility for activities such as informed consent and communication of genomic results varies internationally according to local regulation, workforce models, and scope-of-practice frameworks. The complexity associated with multigene testing, variants of uncertain significance, familial implications, and evolving interpretation frameworks reinforces the continuing importance of specialist genetics expertise and clearly defined boundaries of practice.^20^^,^^41^^,^^43^ These findings therefore support competency-based integration of genomics into nursing practice while emphasising the need for governance structures, interprofessional collaboration, and escalation pathways that support safe and coordinated precision cancer care delivery.^9^, ^10^, ^11^^,^^15^

**Fig. 2:**
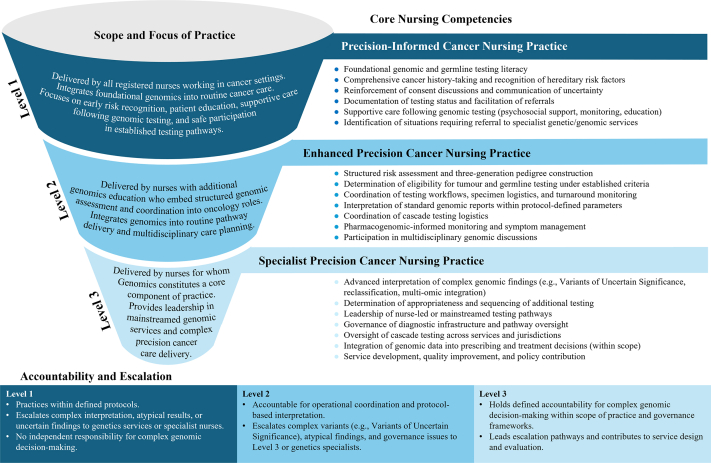
Proposed levels of precision cancer nursing practice.

To support clearer operationalisation of the competencies proposed in this review, a stratified model of precision cancer nursing practice may be helpful. The model distinguishes levels of nursing practice according to increasing competence, autonomy, interpretive complexity, and governance responsibility, rather than implying a uniform or linear career pathway.^17^^,^^18^ This tiered approach differentiates foundational genomic capabilities required of all cancer nurses from the specialist and advanced competencies required for complex precision cancer care delivery. We propose a three-level model of precision cancer nursing practice: 1) precision-informed cancer nursing practice, which is generalist-level practice delivered by all cancer nurses and focused on foundational genomic literacy, risk recognition, supportive care, and safe escalation; 2) enhanced precision cancer nursing practice, undertaken by specialist nurses with additional genomics education, who coordinate testing pathways, conduct structured assessment, interpret standard reports within protocol, and integrate genomics into care planning; and 3) specialist precision cancer nursing practice, undertaken by advanced practice nurses, in which genomics constitutes a core component of practice and includes responsibility for complex interpretation, pathway governance, cascade testing oversight, and service development (Fig. 2). Specialist precision cancer nursing practice as conceptualised in this review does not imply equivalence to the profession of genetic counsellor, which remains a distinct profession with separate educational, regulatory, and credentialing pathways in many jurisdictions.^7^^,^^62^ Rather, the model describes progressively advanced nursing contributions within multidisciplinary precision cancer care systems. It recognises variability in workforce configurations across health systems while preserving patient safety through clear scope, accountability, interprofessional collaboration, and escalation pathways. Progression across the model reflects increasing responsibility for independent assessment, interpretation of genomic information, management of uncertainty, coordination of testing and referral pathways, interprofessional decision-making, and service governance. Levels are not intended as hierarchical career mandates, but as functional distinctions in scope, accountability, and complexity within precision cancer care delivery.

Educational provision largely emphasises foundational competence. Programmes commonly prioritise genetics and genomics fundamentals, inheritance, ethical and legal principles, consent processes, and communication skills, aligning with established baseline genomic nursing competencies.^38^^,^^42^^,^^71^ Yet evidence indicates that clinicians’ confidence and competence in applied genomics remain limited, particularly in interpreting results, determining actionability, and communicating germline implications arising from tumour profiling.^20^ While education builds literacy, it does not consistently operationalise the competencies required for specialist and advanced roles, including nurse-led initiation of testing pathways, management of variants of uncertain significance, and oversight of downstream risk management and follow-up identified within this review. This gap becomes more pronounced as precision cancer care evolves into a multidisciplinary, data-intensive ecosystem. Advances in multi-omics, companion diagnostics, pharmacogenomics, and artificial intelligence are reshaping cancer care and require workforce upskilling across the health system.^13^^,^^21^ Contemporary conceptualisations of precision health nursing extend beyond testing-related counselling to encompass research leadership, data stewardship, system redesign, and leadership of care pathways that require risk-adapted surveillance, tailored education, behavioural support, and programme development.^13^^,^^14^

A distinction also emerges between peer-reviewed education initiatives and academic education programmes identified in the grey literature. Education described within peer-reviewed oncology mainstreaming contexts more frequently links curriculum content to defined nursing roles and applied competencies relevant to clinical pathways.^4^^,^^5^^,^^45^ In contrast, many academic programmes identified in this review are situated within biotechnology, biomedical science, or medical faculties and emphasise sequencing technologies, molecular diagnostics, data analytics, and pharmacogenomics. This distribution reflects broader mapping of precision medicine education, which demonstrates the prominence of science- and technology-oriented departments in delivering MSc-level programmes.^21^ While these scientific foundations are essential, their dominance suggests that postgraduate provision may be driven more by technological innovation than by clearly articulated nursing scope and accountability.

Relatively few programmes explicitly describe specialist nursing pathways in precision health, and only a small subset focus specifically on cancer care. Interdisciplinary education is increasingly emphasised and appropriately reflects the collaborative nature of precision cancer care.^4^^,^^41^^,^^53^ However, many interprofessional education models emphasise the development of shared competencies and collaborative literacy, with comparatively less explicit attention to structured preparation for discipline-specific accountability, escalation thresholds, and leadership within service delivery.^72^ Without explicit integration of applied decision-making and pathway governance into curricula, there is a risk that programmes privilege technological competence over clinical and operational competence. Postgraduate nursing education must therefore support interprofessional collaboration, the development of interpretive expertise, participation in molecular tumour boards, engagement in data stewardship, and leadership in implementation and evaluation within precision cancer care.^13^^,^^73^

Overall, while precision cancer care nursing roles are expanding in scope and complexity, alignment between role expectations, competency frameworks, and educational preparation remains incomplete. The tiered model of precision cancer nursing practice, supported by postgraduate education pathways, may provide a pragmatic mechanism to align education, regulation, and service delivery, enabling specialist and advanced nursing roles to progress from system-enabling participation to system-leading stewardship within precision cancer care.

Key limitations apply to both peer-reviewed and grey literature. The search strategy did not include specific genetic counselling search terms; this may have limited the retrieval of studies describing nursing engagement in counselling-related activities such as risk assessment, informed consent, results communication, and family-risk support. Peer-reviewed searches were restricted to English-language publications, published since 2015, and to title/abstract fields, which may have missed relevant studies and contributed to selection and reporting bias. The grey literature search used a structured, generative AI-assisted approach, with independent human verification against institutional webpages; however, recently updated, poorly indexed programmes, or those not publicly described, may have been missed; therefore, the findings represent a time-bound snapshot of education offerings for nurses in precision cancer care.

The convergent integrated approach adopted in this review enabled synthesis of qualitative, quantitative, mixed methods, and grey literature sources; however, integrating heterogeneous evidence presented methodological challenges given variations in design, reporting depth, and contextual specificity. Quantitative findings were transformed into qualitative descriptions to support integrated thematic analysis, which may have reduced methodological distinctiveness between evidence types. Consistent with scoping review methodology, the synthesis was intended to map and organise the existing evidence base rather than determine effectiveness or establish definitive role boundaries. The resulting themes should therefore be interpreted as an integrative conceptual synthesis of the current literature.

This review demonstrates that nurses contribute across the precision cancer care pathway, spanning genomic risk identification, patient-family communication, supportive care, pathway coordination, interprofessional integration, and professional governance. However, alignment between role expectations, competency frameworks, and educational preparation is uneven. Education and training most consistently support foundational genomic literacy, ethics, and communication, whereas preparation is less explicit for roles requiring professional autonomy, interpretive judgement, and governance of testing pathways. As genomic testing is increasingly mainstreamed within oncology services, this gap risks constraining nursing contributions to system-enabling functions rather than enabling safe expansion into system-leading roles.

To support operationalisation of the competencies proposed, we advance a stratified model of precision cancer nursing practice comprising three functional levels; 1) precision-informed cancer nursing practice; 2) enhanced precision cancer nursing practice; and 3) specialist precision cancer nursing practice. This tiered approach differentiates foundational capabilities required of all cancer nurses from the advanced competencies needed for complex interpretation, pathway oversight, and service development, while preserving patient safety through defined escalation pathways, scope, and accountability. Future research should evaluate whether tiered education models improve competency attainment and patient outcomes and examine how regulatory and service contexts enable or constrain nurses’ scope and accountability within mainstreamed genomic care.

## Contributors

AD was responsible for the concept and design of this study, with support from MS, FH, PF, MT, KRH, SCL, MD. AD supervised the researchers involved in this study (AC, SS). AD conducted the literature searches. AD and AC undertook data extraction. AD, SS, AC and MD accessed and verified the data. AD wrote the data analysis plan and analysed the data, with support from SS and MD. AD, SS, MS, FH, PF, MT, KRH, SCL, MD engaged in the interpretation of the data. AD prepared the draft manuscript. All authors (AD, SS, AC, MS, FH, PF, MT, KRH, SCL, MD) critically reviewed and approved the paper.

## Data sharing statement

The datasets generated and analysed during the current study are not publicly available. Excerpts of data are available from the corresponding author upon reasonable request.

## Declaration of interests

SS is in receipt of a Research Ireland Government of Ireland Postgraduate Scholarship.
