# Supplementary material for: Nursing roles, competencies, and education in precision oncology: a scoping review
Source: eClinicalMedicine. 2026 Jul 21;98:104080. doi: 10.1016/j.eclinm.2026.104080 (PMC13396616; doi:10.1016/j.eclinm.2026.104080)
Supplement: Appendix 1 [file mmc1.docx]

**Appendix 1: Search Strategy**

***MEDLINE***

| S# | Query | Results | Search Date |
| --- | --- | --- | --- |
| S7 | ((MH "Precision Medicine" OR MH "Genomics" OR MH "Genetic Testing") OR (XB "precision medicine" OR "precision oncology" OR "precision cancer care" OR "personalised medicine" OR "personalized medicine" OR "personalised cancer" OR "personalized cancer" OR "genomics" OR "cancer genomics" OR "precision cancer medicine" OR "precision health" OR "omics" OR "genetics")) AND ((MH "Nurses" OR MH "Nursing") OR (XB "nurse" OR "nurses" OR "nursing")) | 2069 | 18-Jul-25 |
| S6 | (MH "Nurses" OR MH "Nursing") OR (XB "nurse" OR "nurses" OR "nursing") | 588324 | 18-Jul-25 |
| S5 | (MH "Precision Medicine" OR MH "Genomics" OR MH "Genetic Testing") OR (XB "precision medicine" OR "precision oncology" OR "precision cancer care" OR "personalised medicine" OR "personalized medicine" OR "personalised cancer" OR "personalized cancer" OR "genomics" OR "cancer genomics" OR "precision cancer medicine" OR "precision health" OR "omics" OR "genetics") | 370922 | 18-Jul-25 |
| S4 | XB "nurse" OR "nurses" OR "nursing" | 549427 | 18-Jul-25 |
| S3 | MH "Nurses" OR MH "Nursing" | 97846 | 18-Jul-25 |
| S2 | XB "precision medicine" OR "precision oncology" OR "precision cancer care" OR "personalised medicine" OR "personalized medicine" OR "personalised cancer" OR "personalized cancer" OR "genomics" OR "cancer genomics" OR "precision cancer medicine" OR "precision health" OR "omics" OR "genetics" | 269165 | 18-Jul-25 |
| S1 | MH "Precision Medicine" OR MH "Genomics" OR MH "Genetic Testing" | 150686 | 18-Jul-25 |

***CINAHL***

| S# | Query | Results | Search Date |
| --- | --- | --- | --- |
| S7 | ((MH "Precision Medicine" OR MH "Genomics" OR MH "Genetic Testing") OR (XB "precision medicine" OR "precision oncology" OR "precision cancer care" OR "personalised medicine" OR "personalized medicine" OR "personalised cancer" OR "personalized cancer" OR "genomics" OR "cancer genomics" OR "precision cancer medicine" OR "precision health" OR "omics" OR "genetics")) AND ((XB "nurse" OR "nurses" OR "nursing") OR (MH "Nurses" OR MH "Nursing")) | 1488 | 18-Jul-25 |
| S6 | (XB "nurse" OR "nurses" OR "nursing") OR (MH "Nurses" OR MH "Nursing") | 653908 | 18-Jul-25 |
| S5 | (MH "Precision Medicine" OR MH "Genomics" OR MH "Genetic Testing") OR (XB "precision medicine" OR "precision oncology" OR "precision cancer care" OR "personalised medicine" OR "personalized medicine" OR "personalised cancer" OR "personalized cancer" OR "genomics" OR "cancer genomics" OR "precision cancer medicine" OR "precision health" OR "omics" OR "genetics") | 38148 | 18-Jul-25 |
| S4 | XB "nurse" OR "nurses" OR "nursing" | 624349 | 18-Jul-25 |
| S3 | MH "Nurses" OR MH "Nursing" | 76121 | 18-Jul-25 |
| S2 | XB "precision medicine" OR "precision oncology" OR "precision cancer care" OR "personalised medicine" OR "personalized medicine" OR "personalised cancer" OR "personalized cancer" OR "genomics" OR "cancer genomics" OR "precision cancer medicine" OR "precision health" OR "omics" OR "genetics" | 30420 | 18-Jul-25 |
| S1 | MH "Precision Medicine" OR MH "Genomics" OR MH "Genetic Testing" | 11229 | 18-Jul-25 |

***PsycINFO***

| S# | Query | Results (count) | Search Date |
| --- | --- | --- | --- |
| S7 | ((DE "Precision Medicine" OR DE "Genomics") OR (XB "precision medicine" OR "precision oncology" OR "precision cancer care" OR "personalised medicine" OR "personalized medicine" OR "personalised cancer" OR "personalized cancer" OR "genomics" OR "cancer genomics" OR "precision cancer medicine" OR "precision health" OR "omics" OR "genetics")) AND ((DE "Nurses" OR DE "Nursing") OR (XB "nurse" OR "nurses" OR "nursing")) | 349 | 18-Jul-25 |
| S6 | (DE "Nurses" OR DE "Nursing") OR (XB "nurse" OR "nurses" OR "nursing") | 123585 | 18-Jul-25 |
| S5 | (DE "Precision Medicine" OR DE "Genomics") OR (XB "precision medicine" OR "precision oncology" OR "precision cancer care" OR "personalised medicine" OR "personalized medicine" OR "personalised cancer" OR "personalized cancer" OR "genomics" OR "cancer genomics" OR "precision cancer medicine" OR "precision health" OR "omics" OR "genetics") | 25057 | 18-Jul-25 |
| S4 | XB "nurse" OR "nurses" OR "nursing" | 120074 | 18-Jul-25 |
| S3 | DE "Nurses" OR DE "Nursing" | 57831 | 18-Jul-25 |
| S2 | XB "precision medicine" OR "precision oncology" OR "precision cancer care" OR "personalised medicine" OR "personalized medicine" OR "personalised cancer" OR "personalized cancer" OR "genomics" OR "cancer genomics" OR "precision cancer medicine" OR "precision health" OR "omics" OR "genetics" | 23658 | 18-Jul-25 |
| S1 | DE "Precision Medicine" OR DE "Genomics" | 3313 | 18-Jul-25 |

***EMBASE***

| No. | Query | Results | Date |
| --- | --- | --- | --- |
| #12 | ('precision medicine'/exp OR 'genomics'/exp OR 'genetics'/exp) AND ('precision medicine':ti,ab OR 'precision oncology':ti,ab OR 'precision cancer care':ti,ab OR 'personalised medicine':ti,ab OR 'personalized medicine':ti,ab OR 'personalised cancer':ti,ab OR 'personalized cancer':ti,ab OR 'genomics':ti,ab OR 'cancer genomics':ti,ab OR 'precision cancer medicine':ti,ab OR 'precision health':ti,ab OR 'omics':ti,ab OR 'genetics':ti,ab) | 199680 | 18-Jul-25 |
| #11 | ('precision medicine':ti OR 'precision oncology':ti OR 'precision cancer care':ti OR 'personalised medicine':ti OR 'personalized medicine':ti OR 'personalised cancer':ti OR 'personalized cancer':ti OR 'genomics':ti OR 'cancer genomics':ti OR 'precision cancer medicine':ti OR 'precision health':ti OR 'omics':ti OR 'genetics':ti) AND ('nurse':ti OR 'nurses':ti OR 'nursing':ti) | 440 | 18-Jul-25 |
| #10 | 'nurse':ti OR 'nurses':ti OR 'nursing':ti | 331296 | 18-Jul-25 |
| #9 | 'precision medicine':ti OR 'precision oncology':ti OR 'precision cancer care':ti OR 'personalised medicine':ti OR 'personalized medicine':ti OR 'personalised cancer':ti OR 'personalized cancer':ti OR 'genomics':ti OR 'cancer genomics':ti OR 'precision cancer medicine':ti OR 'precision health':ti OR 'omics':ti OR 'genetics':ti | 108385 | 18-Jul-25 |
| #8 | ('precision medicine':ti,ab OR 'precision oncology':ti,ab OR 'precision cancer care':ti,ab OR 'personalised medicine':ti,ab OR 'personalized medicine':ti,ab OR 'personalised cancer':ti,ab OR 'personalized cancer':ti,ab OR 'genomics':ti,ab OR 'cancer genomics':ti,ab OR 'precision cancer medicine':ti,ab OR 'precision health':ti,ab OR 'omics':ti,ab OR 'genetics':ti,ab) OR ('nurse':ti,ab OR 'nurses':ti,ab OR 'nursing':ti,ab) | 1050474 | 18-Jul-25 |
| #7 | 3 AND 6 | 24862852 | 18-Jul-25 |
| #6 | 4 OR 5 | 38581696 | 18-Jul-25 |
| #5 | 'nurse':ti,ab OR 'nurses':ti,ab OR 'nursing':ti,ab | 691396 | 18-Jul-25 |
| #4 | 'nurse'/exp OR 'nursing'/exp | 637929 | 18-Jul-25 |
| #3 | 1 OR 2 | 47895990 | 18-Jul-25 |
| #2 | 'precision medicine':ti,ab OR 'precision oncology':ti,ab OR 'precision cancer care':ti,ab OR 'personalised medicine':ti,ab OR 'personalized medicine':ti,ab OR 'personalised cancer':ti,ab OR 'personalized cancer':ti,ab OR 'genomics':ti,ab OR 'cancer genomics':ti,ab OR 'precision cancer medicine':ti,ab OR 'precision health':ti,ab OR 'omics':ti,ab OR 'genetics':ti,ab | 361248 | 18-Jul-25 |
| #1 | 'precision medicine'/exp OR 'genomics'/exp OR 'genetics'/exp | 1590196 | 18-Jul-25 |

***Scopus***

| No. | Query | Results | Date |
| --- | --- | --- | --- |
| #6 | ( TITLE ( "precision medicine" OR "precision oncology" OR "precision cancer care" OR "personalised medicine" OR "personalized medicine" OR "personalised cancer" OR "personalized cancer" OR "genomics" OR "cancer genomics" OR "precision cancer medicine" OR "precision health" OR "omics" OR "genetics" ) ) AND ( TITLE ( "nurse" OR "nurses" OR "nursing" ) ) | 475 | 18-Jul-25 |
| #5 | TITLE ( "nurse" OR "nurses" OR "nursing" ) | 373,318 | 18-Jul-25 |
| #4 | TITLE ( "precision medicine" OR "precision oncology" OR "precision cancer care" OR "personalised medicine" OR "personalized medicine" OR "personalised cancer" OR "personalized cancer" OR "genomics" OR "cancer genomics" OR "precision cancer medicine" OR "precision health" OR "omics" OR "genetics" ) | 121,107 | 18-Jul-25 |
| #3 | ( TITLE-ABS-KEY ( "precision medicine" OR "precision oncology" OR "precision cancer care" OR "personalised medicine" OR "personalized medicine" OR "personalised cancer" OR "personalized cancer" OR "genomics" OR "cancer genomics" OR "precision cancer medicine" OR "precision health" OR "omics" OR "genetics" ) ) AND ( TITLE-ABS-KEY ( "nurse" OR "nurses" OR "nursing" ) ) | 7,187 | 18-Jul-25 |
| #2 | TITLE-ABS-KEY ( "nurse" OR "nurses" OR "nursing" ) | 1,022,244 | 18-Jul-25 |
| #1 | TITLE-ABS-KEY ( "precision medicine" OR "precision oncology" OR "precision cancer care" OR "personalised medicine" OR "personalized medicine" OR "personalised cancer" OR "personalized cancer" OR "genomics" OR "cancer genomics" OR "precision cancer medicine" OR "precision health" OR "omics" OR "genetics" ) | 3,162,585 | 18-Jul-25 |
