# Supplementary material for: Nursing roles, competencies, and education in precision oncology: a scoping review
Source: eClinicalMedicine. 2026 Jul 21;98:104080. doi: 10.1016/j.eclinm.2026.104080 (PMC13396616; doi:10.1016/j.eclinm.2026.104080)
Supplement: Appendix 2 [file mmc2.docx]

**Appendix 2: Generative AI Search Strategy**

***Generative AI Search Strategy Summary***

To map international education provision relevant to nursing preparation for precision oncology and genomics, we conducted a structured grey literature search using a custom GPT operating in Deep Research mode (i.e., with real-time web browsing enabled). The LLM was used as an assisted web-retrieval and synthesis interface, not as a standalone knowledge source. Searches targeted university and third-level institution programme pages, academic programme portals, and other recognised education providers.

A standardised prompt protocol governed all searches. The objective was to identify accredited postgraduate programmes (MSc, PGDip, PGCert) and university-administered/credited CPD options (including certificates, microcredentials, and short courses) that were accessible to registered nurses or broader healthcare professional audiences inclusive of nurses, and that explicitly included content in precision medicine/personalised cancer care, genomics/genetics/omics, molecular diagnostics, or genetic counselling. Undergraduate programmes, physician-only or laboratory-scientist-only programmes, non-healthcare programmes, and non-accredited workshops/conferences were excluded.

Search execution occurred sequentially by world region (Europe; Africa; Asia; North America; South America; Oceania). Within each region, the model was instructed to search country-by-country, in English or the country’s official language, using predefined Boolean query structures (e.g., (“precision medicine” OR “precision oncology” OR “cancer genomics” OR “omics” OR “genetics”) AND (“nurse” OR “nursing”) AND [Country Name]). The protocol further instructed the model to check major universities in each country and to include international online programmes or multi-country initiatives where accessible to nurses across jurisdictions.

For each eligible programme, the model produced a structured output table with programme title, country, institution, qualification level, and notes (including international/online access where stated). Where no eligible programme was identified for a country, this was recorded as a null finding.

Validation was conducted as a second-stage process: the compiled programme list from the regional searches was resubmitted to the model, with instructions to cross-check for omissions and propose additional eligible programmes. All candidate programmes identified via the searches were screened by the research team via direct inspection of source webpages. Final inclusion and programme-level data extraction were based on manual verification, with programme de-identification undertaken for publication where required.

***Custom Large Language Model Configuration – ChatGPT v4.2***

*Description*

This chatbot is designed to locate postgraduate education programmes for nurses in the areas of precision medicine, precision oncology, genomics, and related fields with a specialist focus on cancer.

*Instructions*

Objective: Identify postgraduate-level programmes (e.g., MSc, PGDip, PGCert), Continuing Professional Development, microcredentials, or short courses relevant to precision medicine, personalised cancer care, genomics, or omics that are accessible to registered nurses, and return a structured table per country.

1. Search Scope: Geographic target: One country at a time (supplied by the user); Language: Include results in English or the country’s official language; Institution types: Universities, hospitals, CPD providers; Programme levels to include: 1) MSc, PGDip, PGCert, 2) Certificate (postgraduate or continuing professional development), 3) Short CPD courses, 4) Microcredentials.

2. Inclusion Criteria: Only include programmes that: 1) Explicitly mention nurses or healthcare professionals as a target audience, 2) Offer content in: Precision medicine / Personalised medicine; Genomics / Genetics / Omics (proteomics, transcriptomics, etc.); Molecular diagnostics/genetics/genetic counselling, and 3) are administered and/or accredited by a university or third level institution. Programmes may have a general precision medicine focus, or may be a Cancer care / Oncology / haematology programme which offers modules or content related to precision medicine.

3. Exclusion Criteria: 1) Undergraduate or Bachelor-level nursing programmes, 2) Programmes exclusively for physicians or lab-based scientists, 3) Programmes unrelated to genomics or cancer, 4) Courses with no healthcare relevance, and 5) conferences, workshops and eLearning programmes that are not accredited by a university or third-level institution.

4. Output Format: For each relevant programme, return the following columns in a structured table: Programme Title Country Institution Level of Qualification Notes Where a country offers a programme that is open to international applicants, please make this clear in the notes

5. Search Strategy and Tools: Use reliable sources: official university or third level institution websites, academic programme portals, international health organisations

Use advanced search queries such as: ("precision medicine" OR "precision oncology" OR "precision cancer care" OR "personalised medicine" OR "personalized medicine" OR "personalised cancer" OR "personalized cancer" OR "genomics" OR "cancer genomics" OR "precision cancer medicine" OR "precision health" OR "omics" OR "genetics") AND ("nurse" OR "nurses" OR "nursing") AND [Country Name]

When searching, search all major universities in the country of interest.

Please search for 1) MSc, PGDip, PGCert, 2) Certificate (postgraduate or continuing professional development), 3) Short CPD courses, 4) Microcredentials.

Be prepared to search for online universities, international universities.

6. If No Results Are Found: Return a single-row table with empty cells except for the country name, e.g.: Programme Title Country Institution Level of Qualification - Notes — Latvia — —

*Recommended Model*

No Recommended Model - Users will use any model they prefer

*Capabilities*

Web Search

***CustomGPT Chat Prompt Strategy***

"I am trying to identify all potential postgraduate education available for nurses working in cancer care that focuses on precision cancer care. Could you do a global search for relevant education programmes, using the following protocol: Objective: Identify postgraduate-level programmes (e.g., MSc, PGDip, PGCert), Continuing Professional Development, microcredentials, or short courses relevant to precision medicine, personalised cancer care, genomics, or omics that are accessible to registered nurses, and return a structured table per country.

1. Search Scope: Geographic target: One continent at a time (country by country); Language: Include results in English or the country’s official language; Institution types: Universities, hospitals, CPD providers; Programme levels to include: 1) MSc, PGDip, PGCert, 2) Certificate (postgraduate or continuing professional development), 3) Short CPD courses, 4) Microcredentials.

2. Inclusion Criteria: Only include programmes that: 1) Explicitly mention nurses or healthcare professionals as a target audience, 2) Offer content in: Precision medicine / Personalised medicine; Genomics / Genetics / Omics (proteomics, transcriptomics, etc.); Molecular diagnostics/genetics/genetic counselling, and 3) are administered and/or accredited by a university or third level institution. Programmes may have a general precision medicine focus, or may be a Cancer care / Oncology / haematology programme which offers modules or content related to precision medicine.

3. Exclusion Criteria: 1) Undergraduate or Bachelor-level nursing programmes, 2) Programmes exclusively for physicians or lab-based scientists, 3) Programmes unrelated to genomics or cancer, 4) Courses with no healthcare relevance, and 5) conferences, workshops and eLearning programmes that are not accredited by a university or third-level institution.

4. Output Format: For each relevant programme, return the following columns in a structured table: Programme Title Country Institution Level of Qualification Notes

Where a country offers a programme that is open to international applicants, please make this clear in the notes

5. Search Strategy and Tools: Use reliable sources: official university or third level institution websites, academic programme portals, international health organisations. Use advanced search queries such as: (""precision medicine"" OR ""precision oncology"" OR ""precision cancer care"" OR ""personalised medicine"" OR ""personalized medicine"" OR ""personalised cancer"" OR ""personalized cancer"" OR ""genomics"" OR ""cancer genomics"" OR ""precision cancer medicine"" OR ""precision health"" OR ""omics"" OR ""genetics"") AND (""nurse"" OR ""nurses"" OR ""nursing"") AND [Country Name].

When searching, search all major universities in the country of interest.

Please search for 1) MSc, PGDip, PGCert, 2) Certificate (postgraduate or continuing professional development), 3) Short CPD courses, 4) Microcredentials.

Be prepared to search for online universities, international universities.

6. If No Results Are Found: Return a single-row table with empty cells except for the country name, e.g.: Programme Title Country Institution Level of Qualification - Notes

could you search all countries on the continent of [XXXXXX]?

ok, you don't seem to have searched the following countries:

ok, here are programmes I identified:

"Could you re-run a comprehensive search of all countries, incorporating your newly identified programmes and generate a table summarising all programmes, using the headings Programme Title Country Institution Level of Qualification Notes

Don't allow overlap or repetition of programmes. Please ensure that general genomics/precision medicine courses but open to nurses (even if not specifically designed for nurses) are included. Please include fully accredited programmes, please include online international programmes and multi-country initiatives in the table, make it clear they are an international programme or multi country initiative and make it clear which countries they serve."

***Generative AI Search Strategies:***

Search strategies used within this search are available upon request from the corresponding author.
