# Supplementary material for: Nursing roles, competencies, and education in precision oncology: a scoping review
Source: eClinicalMedicine. 2026 Jul 21;98:104080. doi: 10.1016/j.eclinm.2026.104080 (PMC13396616; doi:10.1016/j.eclinm.2026.104080)
Supplement: Appendix 3 [file mmc3.docx]

**Appendix 3: Source Document Characteristics: Nurses’ Roles in Precision Cancer Care**

| **Study Author, year** | **Key Characteristics** | **Nursing role(s) described** |
| --- | --- | --- |
| Adejumo et al., 2021, Nigeria^46^ | Quantitative pilot feasibility study. Aim: To assess feasibility and acceptability of integrating trained nurses into cancer genetic counselling and testing to support cancer risk assessment and patient experience in a Nigerian oncology setting. Service delivery model: Nurse-led outpatient genetic counselling and testing embedded within oncology clinics. | Provide genetic counselling in outpatient oncology clinics for patients with breast, ovarian, and endometrial cancer who are new to genetic counselling/testing Educate patients on hereditary cancer genetics and the benefits/risks of genetic counselling and testing Document personal and family history, including drawing pedigrees Assess mutation risk and explain genetic testing methods and the meaning of results |
| Ajibade & Madu, 2025, USA^61^ | Discussion paper.  Aim: To explore how integration of artificial intelligence into precision medicine for neuro-oncology reshapes clinical practice, ethics, and nursing roles, with a focus on immunotherapy care and patient-centred implications. | Advocate for patient rights, autonomy, and compassionate care in AI-supported clinical decision-making  Educate patients about the use, benefits, risks, and limitations of AI in diagnosis and treatment planning  Translate AI-generated diagnostic and treatment outputs into patient-understandable information  Facilitate informed consent and support shared decision-making around AI-based care  Monitor, evaluate, and provide clinical oversight of AI systems to ensure safety, ethical use, and alignment with patient needs  Safeguard patient privacy and data security in AI-integrated care  Maintain and update AI-related knowledge to support responsible implementation and ongoing service improvement |
| Al-Kaiyat, 2018, Saudi Arabia^48^ | Case Study. Aim: To describe the evolving role of the nurse coordinator in personalised medicine, using a lung cancer clinic case to illustrate precision coordination in oncology. Service delivery model: outpatient lung cancer clinic with nurse-led coordination of molecular testing and care pathways. | Review all scheduled cases in advance to ensure completion of diagnostic workup  Interpret pathology reports to identify and track molecular testing status and results  Maintain and update a central screening log to monitor molecular profiles and tissue availability  Identify patients newly eligible for additional or repeat molecular testing as disease progresses  Coordinate molecular test ordering with pathologists and treating physicians  Arrange liquid biopsies when tissue is insufficient to avoid delays in precision treatment |
| Barbato et al., 2019^60^ | Discussion Paper. Aim: to propose a structured curriculum for integrating genetics and genomics into PhD nursing education and prepare nurse scientists for genomic research. | Conduct genomics- and omics-informed nursing research to advance bio-behavioural science  Perform genetic risk assessment to support health promotion and disease prevention  Communicate genetic risk information clearly and sensitively to patients and families  Address psychosocial, ethical, and self-management needs related to genetic conditions  Build genomics capacity through education, training, and integration into nursing practice |
| Blondeaux et al., 2023, Italy^36^ | Quantitative study Aim: To evaluate patient experience and retention of information following nurse-driven, telephone-based disclosure of uninformative BRCA test results. Service delivery model: Nurse-driven genetic counselling with telehealth (telephone disclosure) | Conduct telephone triage to assess expectations, eligibility, and collect personal/family history with three-generation pedigree  Provide pretest genetic counselling, including BRCA risk education, shared decision-making, and informed consent  Participate in multidisciplinary triage and results meetings with clinical geneticists  Disclose uninformative BRCA test results via telephone using standardized counselling messages  Coordinate follow-up support and referrals (e.g., psychology) based on clinical and psychosocial needs |
| Bokkers et al., 2023^59^ | Quantitative, prospective observational study. Aim: to evaluate patient experiences of pre-test genetic counselling delivered by non-genetic healthcare professionals (surgeons or nurses) and compare them with usual genetic care. Service delivery model: Telehealth/genetic counselling clinic; mainstreamed genetic testing pathways. | Provide pre-test genetic counselling after training and independently order breast cancer susceptibility gene tests  Screen eligibility and post-test counselling needs using standardized checklists and obtain informed consent  Refer patients to genetics specialists when criteria are unmet, results are complex (PV/variants of uncertain significance), or additional family-based risk assessment is required |
| Bokkers et al., 2022^3^ | Quasi-experimental observational study. Aim: to assess healthcare professionals’ attitudes, knowledge, self-efficacy, and feasibility of integrating mainstream germline genetic testing for women with ovarian cancer | Discuss germline genetic testing options and familial implications with eligible women  Provide written information and obtain informed consent for genetic testing  Complete guideline-based checklists to assess need for additional genetic counselling  Refer patients to genetics services based on checklist findings and test results |
| Bracci et al., 2020^45^ | Quantitative prospective observational study. Aim: to evaluate whether a nurse-led, guideline-based semi-structured interview improves identification of cancer patients eligible for genetic counselling and assesses patient interest in counselling. Service delivery model: Nurse-led, in-person screening using a guideline-based interview within routine oncology care. | Conduct semi-structured, guideline-based interviews to identify patients eligible for genetic counselling  Educate patients on the purpose and process of genetic counselling and testing  Administer and support completion of genetic risk questionnaires without influencing responses  Clarify hereditary cancer concepts (e.g., Lynch syndrome, HBOC) to support accurate data collection |
| Brennan-Doran et al., 2024^50^ | Narrative literature review. Aim: to summarize the potential roles of oncology nurses in the genetic testing pathway, with a focus on germline BRCA1/BRCA2 testing. | Provide pre-test genetic counselling, obtain informed consent, and collect samples for gBRCA testing  Deliver genetic test results and provide post-test education or counselling within oncology clinics  Triage results to identify patients requiring referral to genetic counsellors or clinical geneticists  Support patients in understanding results (including variants of uncertain significance) and managing ongoing cancer risk  Facilitate family communication, cascade testing awareness, and longer-term psychosocial support |
| Chair et al., 2019^44^ | Narrative literature review. Aim: to identify issues and challenges in genomics education in nursing in Hong Kong, Taiwan, and Mainland China. | Support physician-led genetic counselling services in centralized and private settings  Provide genetic/genomic testing support following short-term or ad hoc specialist training  Assist with interpretation and explanation of genetic test results, including patient-paid tests  Conduct genetic screening and contribute to pre- and post-test counselling where services are limited  Coordinate patient follow-up and ongoing clinical care in genetics-related pathways |
| Chiu et al., 2024^62^ | Policy analysis. Aim: to learn from genomics-informed oncology nursing policy in the US and UK to inform policy development for genomics-informed oncology nursing practice and education in Canada. | Assess and integrate genomic information into patient care across settings  Plan, implement, and evaluate genomics-informed interventions and outcomes  Advocate for ethical, equitable, and evidence-based genomic practice through collaboration, education, and leadership |
| Colomer-Lahiguera et al., 2024^13^ | Discussion paper Aim: to explore the opportunities and challenges for advanced practice nurses (APNs) within the precision health paradigm in oncology | Conduct holistic, genomics-informed clinical assessments incorporating biological, environmental, and social determinants  Interpret molecular/genomic data and stratify patients to personalize treatments, prevention, and symptom management  Translate complex data into patient-centred education, coaching, and shared decision-making  Coordinate and lead interprofessional, precision-health pathways across clinical, organizational, and system levels  Integrate EHR, patient-generated data, and AI-enabled tools to guide decision support, quality improvement, and ethical care |
| Dewell et al., 2024^38^ | Discussion paper. Aim: to map the 2021 AACN Essentials to the ANA Essentials of Genomic Nursing and provide exemplar learning outcomes, content, and clinical vignettes to support integration of genomics into undergraduate nursing curricula. | Advocate for patients and families, educate on genomics, and support informed decision-making  Integrate genomic information into coordinated, interprofessional care using a holistic approach  Apply genomic knowledge ethically to implement and evaluate precision healthcare, addressing equity and social determinants |
| Ellard et al., 2022^56^ | Position / clinical practice guideline. Aim: to provide a standardised nursing practice framework for recognition, monitoring, grading, and management of CAR-T therapy-associated toxicities. | Screen psychosocial risk (anxiety/isolation) and coordinate referrals (psychology/counselling, palliative care/ACP)  Educate patient/family on CAR-T eligibility workup, procedures, prophylaxis, line care, and complication prevention  Coordinate pre-CAR-T readiness (infection screening/prophylaxis, bridging therapy considerations, central access planning)  Monitor and manage apheresis care (vein assessment, vitals, detect/treat citrate toxicity, coordinate timing/logistics)  Deliver CAR-T infusion safely (verify identity/consent/prescription, complete pre-checks, administer premeds, observe reactions, document; alert ICU/neuro teams)  Provide intensive post-infusion surveillance (frequent vitals/ECG/labs, fluid balance/weights, daily handwriting/ICE screening)  Detect/escalate CAR-T toxicities (CRS/ICANS/HLH-MAS), support symptom care, and communicate changes rapidly to MDT/ICU  Provide discharge education and long-term follow-up support (symptom triggers/urgent return, proximity advice, QoL assessment, health promotion, appointment coordination/reporting) |
| Flynn et al., 2019^30^ | Case study. Aim: to describe how a large academic clinical research hospital integrates genomics into oncology nursing practice using adapted genomic competencies and the MINC toolkit. Service delivery model: Institution-wide nursing education and competency-based implementation across inpatient and outpatient oncology settings. | Help patients interpret and process genetic/genomic information  Help patients formulate questions for the medical team  Translate genetic/genomic information for family members |
| Forman & Schwartz, 2019^16^ | Discussion paper. Aim: to build oncology nursing skills in cancer risk assessment, including identification of hereditary cancer risk, use of risk models, and delivery of risk education and informed consent. | Conduct comprehensive cancer risk assessment using personal, medical, and family history  Construct and document multigenerational cancer pedigrees to identify hereditary risk  Educate patients on genetic testing implications and obtain informed consent  Assess psychosocial readiness and provide emotional support throughout testing  Coordinate referrals, communicate results, and support ongoing risk-based follow-up and surveillance |
| Godino et al., 2025^35^ | Qualitative phenomenological study. Aim: to understand the perceived roles and activities of nurses employed in genetic clinics. | Provide holistic genetic nursing care across physical, psychosocial, ethical, and spiritual domains for patients and families  Conduct telephone triage to clarify needs, filter inappropriate requests, and prioritise referrals  Collect and document family history and clinical data to support genetic risk assessment  Support the multiprofessional pre-test and post-test pathway, including coordinating steps and timelines for testing  Communicate low-risk/straightforward post-test results when appropriate and act as an ongoing emotional support point-of-contact |
| Hayden et al., 2022^57^ | Practice guideline. Aim: best-practice recommendations to support healthcare professionals in delivering consistent, high-quality care for adults and children receiving CAR-T cell therapy. | Administer CAR-T promptly post-thaw using aseptic non-touch technique and correct giving set  Record vital signs pre/during/post infusion; monitor and treat infusion reactions symptomatically; avoid steroids unless critically unwell  Dispose of CAR-T materials as GMO biohazard per local/national regulations  Screen for and help prevent/manage tumour lysis syndrome using local protocols  Detect infection early in neutropenia; trigger cultures/imaging/viral testing and start empiric antimicrobials per protocol  Monitor for CRS and differentiate from sepsis; support CRS management (fluids/antipyretics, escalate for tocilizumab/steroids)  Monitor for CRS/MAS overlap and support intensified lab surveillance and escalation for anakinra/steroids  Perform structured neurotoxicity surveillance (ICE/CAPD, handwriting checks) and escalate suspected ICANS; support seizure management and ICU transfer when indicated  Support cardiovascular surveillance (baseline “dry weight”, daily weights/overload signs) and escalate for cardiac review/testing  Coordinate medium-term follow-up labs/virology/immune monitoring and prophylaxis adherence  Monitor/manage hypogammaglobulinemia and B-cell aplasia (arrange immunoglobulin replacement when indicated)  Support vaccination planning and immune-response monitoring where feasible  Monitor delayed cytopenia and coordinate investigations and supportive therapies (e.g., G-CSF timing)  Participate in long-term follow-up/registry reporting and liaise with referral centers to sustain shared-care protocols |
| Hines-Dowell et al., 2024^31^ | Mixed methods study. Aim: to identify perceived and actual barriers to genomic education, clarify nurses’ roles in genomics use, and determine preferred methods for genomics education in paediatric oncology. | Educate patients and families about genomics  Take/record family history  Request referrals to genetic services  Explain different genetic testing options  Explain genetic test results |
| Hoopes et al., 2022^53^ | Case study. Aim: to review an example of the oncology nurse role in a clinic designed to manage hereditary cancer risk and to highlight nursing implications. Service delivery model: clinic-based hereditary cancer risk management with annual follow-up and direct access to an oncology nurse navigator as needed. | Navigate patients with pathogenic variants through personalized cancer surveillance and risk management pathways  Collaborate with genetic counsellors to apply up-to-date NCCN screening and prevention guidelines  Update clinical and family history and reassess variant-specific screening needs annually  Educate patients on hereditary cancer risk, enhanced screening, and risk-reducing options using shared decision-making  Coordinate referrals for imaging, specialist care, and prophylactic surgery when indicated  Support risk-reducing lifestyle change through motivational interviewing and targeted referrals  Serve as a consistent point of contact to ensure follow-through, address concerns, and reduce care fragmentation |
| Kerber & Ledbetter, 2017b^28^ | Discussion paper with illustrative case studies. Aim: to describe evolving cancer nursing roles with the implementation of genomics and genetics standards in oncology practice, with a focus on the graduate nurse. | Assess personal and family history to identify genetic risk  Educate patients and families on genetics, testing, and implications  Identify high-risk individuals and initiate referrals to genetics professionals  Provide psychosocial support and reinforce risk-reducing behaviours  Personalize surveillance, prevention, and management using genetic guidelines  Coordinate care, protect genetic information, and support informed decision-making  Evaluate outcomes of testing and treatments and adjust care plans  Maintain genetic/genomic competence through ongoing education and evidence-based practice |
| Kerber & Ledbetter, 2017a^42^ | Discussion paper with illustrative case studies. Aim: to describe how the advanced practice nurse role evolves with the implementation of genomics and genetics standards in oncology practice. Service delivery model: APN-led genetic counselling services integrated into oncology care | Identify patients with increased genetic risk and evaluate personal and family history Collect and analyse pedigrees to assess inherited cancer predisposition Provide genetic information, counselling, and anticipatory guidance to patients and families Initiate, recommend, and interpret genetic and genomic tests within scope of practice Personalize surveillance, treatment, and care plans using genomic evidence and guidelines Support informed decision-making and address psychosocial impacts on patients and families Refer and collaborate with genetic counsellors, physicians, and interdisciplinary teams Protect genetic information and uphold ethical, legal, and privacy standards Mentor colleagues, contribute to education, and maintain genomics competence through continuing professional development |
| Loughrey et al., 2025^2^ | Quantitative, retrospective observational study. Aim: to evaluate the feasibility and effectiveness of an advanced nurse practitioner (ANP)-led. mainstreaming service for diagnosing Lynch syndrome in patients with colorectal cancer. Service delivery model: Nurse-led outpatient service with integration into multidisciplinary team (MDT) meetings. | Lead a familial colorectal clinic embedded in surgical services to manage hereditary CRC risk  Participate in multidisciplinary meetings to guide Lynch syndrome pathways and care decisions  Facilitate and coordinate mainstream genetic testing for newly diagnosed CRC patients  Oversee diagnostic workflows (IHC, reflex testing, germline and somatic analysis) to distinguish hereditary from sporadic disease  Identify, consent, and refer eligible patients for constitutional genetic testing and follow-up  Track, document, and evaluate genetic testing outcomes and pathway timelines |
| Mahon & Yackzan, 2022^43^ | Case study. Aim: To illustrate scope-of-practice and competency considerations for oncology nurse practitioners ordering and managing germline genetic testing. Setting / service delivery model: Oncology practice context involving germline genetic testing and referral to genetic counselling services. | Order appropriate genetic tests and accurately interpret results, or refer to genetics professionals when outside scope  Manage patients and families based on personal and family history, including variants of uncertain significance  Stay current with evolving genetic evidence, variant reclassification, and testing guidelines  Coordinate cascade testing for at-risk relatives to support prevention and early detection  Integrate genetic results into individualized patient and family management plans |
| McAllister & Schmitt, 2015^55^ | Quantitative, retrospective observational study. Aim: to define best practices for identifying appropriate breast cancer patients for genomic testing and to improve the timeliness of test ordering and result reporting. Service delivery model: Inpatient oncology service with integrated nurse navigation. | Screen and identify patients eligible for genetic testing using a tracking tool  Coordinate and schedule oncology appointments aligned with test result availability  Educate patients on genetic testing and implications of results  Order genetic tests once definitive pathology is available (APN role)  Monitor turnaround times and provider adherence to NCCN guidelines and report outcomes |
| McReynolds & Connors, 2019^32^ | Case study. Aim: to review the current state of genomics and genetic testing in prostate cancer and highlight implications for nursing practice. | Elicit detailed personal and family cancer histories across repeated patient encounters  Identify hereditary cancer “red flags” and stratify patients for genetic referral  Encourage family communication about inherited cancer risk  Facilitate referrals to genetics specialists for appropriate evaluation  Educate patients (including men) on the implications and optional nature of genetic counselling and testing |
| Miller & Rosenzweig, 2021^49^ | Discussion paper. Aim: to describe how oncology nurses contribute to biobanking and support precision medicine through patient consent, specimen collection, and care coordination. | Identify eligible patients and procedures where biospecimens can be collected  Obtain informed consent, ensuring understanding of tissue use, rights, and alternatives  Advocate for biobanking and research participation to patients, families, and the public  Coordinate and support specimen collection in line with consent and protocols  Ensure accurate documentation and compliance throughout the biobanking process |
| Nembaware et al., 2019^37^ | Case study. Aim: to describe the establishment of the African Genomic Medicine Training Initiative (AGMT). | Recruit participants and engage communities in genetics and clinical research  Obtain informed consent and manage culturally appropriate study documentation  Collect, process, and manage biological samples per protocols  Provide genetic, reproductive, and health counselling across clinical and community settings  Identify high-risk individuals, coordinate referrals, and collaborate with multidisciplinary teams |
| Percival et al., 2016^5^ | Quantitative service evaluation. Aim: To report on the establishment and evaluation of an extended clinical nurse specialist role in consenting women for BRCA testing using a mainstreaming model. Service delivery model: Mainstreamed genetic testing embedded in routine oncology clinics. | Identify eligible patients, provide written information, discuss BRCA testing, and obtain informed consent  Order BRCA tests, coordinate blood sampling, document in records, and track results  Communicate negative results, refer positive/complex cases to genetics, and act as key worker throughout the pathway |
| Pierle & Mahon, 2019^41^ | Narrative literature review. Aim: to identify barriers to accessing genetic care, review service delivery models that enhance access to genetic counselling and describe how oncology nurses support and facilitate the genetic counselling process. | Identify patients and families at hereditary cancer risk through family history assessment  Educate patients on genetic risk assessment, panel testing, and potential outcomes (including variants of uncertain significance)  Obtain informed consent and prepare patients for pre- and post-test counselling  Coordinate and triage referrals with genetics professionals using tandem or triage models  Reinforce care recommendations, provide psychosocial support, and prompt re-engagement as guidelines evolve |
| Rahman et al., 2022^20^ | Scoping review. Aim: to identify the genetic and genomic learning needs of oncologists and oncology nurses in the context of cancer and precision medicine. | Facilitate patient access to germline genetic testing across the cancer care continuum  Deliver nurse-led BRCA testing services in breast and ovarian cancer settings  Support patients through diagnosis, treatment, recovery, and palliation with integrated genetic care  Apply genetics and genomics knowledge to guide testing pathways and patient support |
| Scott et al., 2020^4^ | Quantitative, retrospective observational study. Aim: To develop and implement a nurse-led, in-house mainstreaming cancer genetics (MCG). programme for BRCA testing and reduce waiting times. Study design: Retrospective observational service data review (before-after comparison). Setting: Specialist breast cancer centre within an NHS hospital trust. Service delivery model: Nurse-led, in-house mainstreaming cancer genetics service embedded in oncology clinics (outpatient). Sample: 290 Patients undergoing diagnostic BRCA1/2 testing for breast cancer; breast clinical nurse specialists delivering the service. Nursing role: Breast Clinical Nurse Specialists (CNS), NHS Band 7, specialists in breast family history. Nurse training/preparation: Structured genetics training package including online modules, face-to-face genetics education, mentorship, competency assessment, and ongoing CPD with clinical genetics services. | Deliver pre-test counselling, obtain informed consent, order tests, and disclose BRCA results within oncology clinics  Identify eligible patients using clinical, pathology, and family-history criteria; construct and interpret pedigrees  Act as central care coordinators within the MDT, integrating genetic results into surgical and treatment decision-making  Refer and navigate patients and relatives to genetics, psychology, surgery, and risk-reduction services  Lead service development, mentor nurses, maintain genetic databases, and support audit and research activities |
| Shevach et al., 2023^33^ | Quantitative, retrospective observational study. Aim: to evaluate the impact of an on-site nurse-led cancer genetics service on germline genetic testing completion, with a focus on racial equity. Service delivery model: Embedded on-site nurse-led cancer genetics service compared with centralized telegenetics service. | Identify veterans eligible for genetic evaluation using diagnosis, age, and family history  Deliver on-site and tele-genetic consultations, including history intake and values-based counselling  Order guideline-based genetic tests and coordinate long-term follow-up and surveillance |
| So-Shan Mak & Leong-Tat Chan, 2024^58^ | Discussion paper. Aim: to clarify and conceptualize the emerging roles of oncology nurses in genomics medicine and precision health. | Assess genetic/omics risk by collecting family history, ancestry, and drug‐response indicators  Identify patients for genetic, genomic, or pharmacogenomic testing and genetic counselling  Educate patients and families on genomics, testing purpose, and clinical implications  Support precision medication administration using pharmacogenomic principles  Monitor, document, and evaluate genomic‐related drug responses and adverse reactions  Collaborate with multidisciplinary teams to develop personalized, genomics‐informed care plans  Promote illness prevention, public health genomics, and health equity through advocacy |
| Thompson et al., 2022^39^ | Quantitative, retrospective observational study. Aim: to evaluate the effectiveness outcomes of a nurse practitioner-led cancer genetics program. Service delivery model: Nurse practitioner-led genetic screening and surveillance service with genetic counsellor collaboration. | Screen patients for hereditary cancer risk using family history and guideline-based tools  Provide genetics services using shared decision-making and person-centered counselling  Review, interpret, and explain genetic test results and syndromic cancer risks  Coordinate referrals, prophylactic surgeries, and high-risk surveillance pathways  Order, review, and manage surveillance imaging, procedures, labs, and follow-up care  Counsel on modifiable risk factors and initiate chemoprevention when indicated |
