# Supplementary material for: Nursing roles, competencies, and education in precision oncology: a scoping review
Source: eClinicalMedicine. 2026 Jul 21;98:104080. doi: 10.1016/j.eclinm.2026.104080 (PMC13396616; doi:10.1016/j.eclinm.2026.104080)
Supplement: Appendix 4 [file mmc4.docx]

**Appendix 4: Source Document Characteristics: Nurses' Competencies in Precision Cancer Care**

| **Study Author, year** | **Key Characteristics** | **Knowledge and competences described** |
| --- | --- | --- |
| Aiello, 2017^29^ | Narrative literature review. Aim: to provide an overview of the integration of genetics and genomics into nursing education and practice and to identify persistent knowledge gaps and barriers to implementation among nurses. | Elicit comprehensive family health history and construct pedigrees  Conduct holistic health and physical assessments incorporating genomic factors  Analyse assessment findings and evaluate patient genomic knowledge  Identify genetic/genomic risk factors and screen appropriate patients  Develop care plans integrating genetic and genomic information  Address ethical, legal, and societal implications of genomic care  Facilitate referrals to genetic and genomic services  Provide accurate, current genomic education and decision support  Integrate genomic risk into health promotion and disease prevention  Implement and evaluate genomic-based interventions to improve outcomes  Collaborate with interdisciplinary teams and payers  Recognize personal values, maintain competency, and advocate for patient rights |
| Ajibade & Madu, 2025^61^ | Discussion paper.  Aim: To explore how integration of artificial intelligence into precision medicine for neuro-oncology reshapes clinical practice, ethics, and nursing roles, with a focus on immunotherapy care and patient-centred implications. | Use AI technologies to support patient evaluation, care planning, and outcome monitoring  Understand technical operation of AI tools used in neuro-/precision cancer care practice  Apply ethical principles to AI use, protecting patient rights and autonomy  Translate AI-generated insights into patient-cantered, compassionate care  Advocate for human clinical judgment alongside AI-supported decision-making  Monitor and evaluate the safety, effectiveness, and appropriate adoption of AI systems |
| Barbato et al., 2019^60^ | Discussion Paper. Aim: to propose a structured curriculum for integrating genetics and genomics into PhD nursing education and prepare nurse scientists for genomic research. | Assess genetic risk to support cancer prevention and health promotion  Communicate genomic risk, uncertainty, and implications clearly to patients and families  Address psychosocial and ethical impacts of genomic information in care  Support symptom management and patient self-management using genomic insights  Build and maintain genomic literacy across nursing roles and career stages  Integrate genomics into nursing education, practice, and workforce development  Apply and translate genomic research into precision cancer care practice  Identify nursing-sensitive genomic interventions and outcomes  Interprofessional collaboration in genomic research, implementation, and dissemination  Explain core genetic mechanisms (DNA, RNA, protein, inheritance, variation) relevant to cancer  Interpret genetic tests and sequencing results to inform clinical decision-making  Understand laboratory and “omics” methods underpinning precision cancer care  Apply genomic knowledge safely, ethically, and evidence-based in-patient care |
| Calzone et al., 2024^11^ | Literature review and Delphi study. Aim: to update and achieve consensus on essential genomic nursing competencies and outcome indicators applicable to all registered nurses. | Integrate genomics across assessment, prevention, screening, diagnosis, prognosis, treatment selection, and outcome monitoring  Collect and interpret personal, family, and environmental histories using genomic risk information  Identify patients who benefit from genomic information, testing, or specialist referral  Critically analyse clinical data for genomic, ethical, cultural, legal, and social implications  Develop and implement genomics-informed care plans with interdisciplinary teams  Provide tailored genomic education, support informed decision-making, and advocate for patient autonomy  Apply genomics to health promotion, disease prevention, and targeted interventions  Collaborate in delivering genomic care and evaluate the effectiveness of genomic interventions |
| Carpenter-Clawson et al., 2023^51^ | Mixed methods study. Aim: to identify genomic competencies needed by nurses and midwives for genomic mainstreaming in the NHS and to inform a tailored education model. | Understand core genetics and genomics concepts, including inheritance and germline versus somatic variation  Conduct comprehensive family histories and identify patients at high inherited cancer risk  Apply knowledge of genomic services, referral pathways, and clinical genetics team roles  Interpret genomic data and its implications for prevention, diagnosis, prognosis, and patient management  Understand targeted therapies, their mechanisms of action, and clinical application  Integrate legal, ethical, and social considerations into genomic testing and care  Perform genetic consent using appropriate counselling tools |
| Ceylan et al., 2025^54^ | Quasi-experimental study. Aim: to evaluate the impact of a WhatsApp-based educational intervention on nurses’ genetic knowledge and awareness. | Collect comprehensive family histories including first-, second-, and third-degree relatives  Integrate family history as a core component of nursing assessment and decision-making  Recognize clinical relevance of genetic risk across cancers and common chronic diseases  Counsel patients on genetic risk based on family history  Determine when family history warrants genetic counselling or testing referral  Explain benefits, risks, and limitations of genetic testing for common diseases  Adjust screening recommendations based on familial and genetic risk  Access and apply reliable, up-to-date genetics information  Facilitate referrals to genetic services for at-risk patients |
| Chiu et al., 2024^62^ | Policy analysis. Aim: to learn from genomics-informed oncology nursing policy in the US and UK to inform policy development for genomics-informed oncology nursing practice and education in Canada. | Apply up-to-date genomic science to cancer disease processes and treatment options  Perform comprehensive genomic-informed assessments and deliver patient-centred, tailored care  Communicate genomic information clearly, support informed consent, and educate patients and families  Address ethical, legal, social, privacy, and confidentiality issues in genomic care  Recognize personal biases and advocate for equitable access to genomic services  Initiate referrals, support continuity of care, and collaborate across professions  Maintain continuing genomic competence through education, research, and quality improvement  Demonstrate leadership, policy awareness, and professional accountability in precision cancer care |
| Colomer-Lahiguera et al., 2024^13^ | Discussion paper Aim: to explore the opportunities and challenges for advanced practice nurses (APNs) within the precision health paradigm in oncology | Deliver advanced, patient-centred precision cancer care using holistic clinical, biological, environmental, and social data  Integrate genomics, omics, and biomarker information into diagnosis, treatment selection, prevention, and surveillance  Guide and coach patients and families; support shared decision-making and quality of life  Communicate complex genomic and precision medicine information clearly and ethically  Diagnose, prescribe, and treat (NP role) or develop, implement, and translate evidence-based practice (CNS role)  Coordinate multidisciplinary care and lead system-level precision health initiatives  Identify high-risk individuals and implement primary, secondary, and tertiary prevention strategies  Apply ethical principles; protect autonomy, confidentiality, and responsible use of genetic data  Use clinical technologies, decision-support tools, and data interpretation to stratify risk and tailor interventions  Maintain advanced competence through genomics education, research engagement, and lifelong learning  Lead interprofessional collaboration, policy development, and innovation in precision cancer care  Develop foundational competence in artificial intelligence and digital health tools relevant to precision care |
| Dewell et al., 2024^38^ | Discussion paper. Aim: to map the 2021 AACN Essentials to the ANA Essentials of Genomic Nursing and provide exemplar learning outcomes, content, and clinical vignettes to support integration of genomics into undergraduate nursing curricula. | Apply foundational genomic knowledge to personalize nursing care  Integrate genomics into nursing assessment of personal and family history  Identify patients and families who may benefit from genomic information or services  Use appropriate genomic resources while addressing ethical, legal, and social implications  Facilitate autonomous, informed genomic-related decision-making  Refer patients and families to specialized genomic services as needed  Collaborate with interdisciplinary teams to deliver genomics-informed care  Provide tailored education, health promotion, and disease prevention using genomic insights  Evaluate the impact and effectiveness of genomics-based nursing interventions |
| Dickman et al., 2025^15^ | Document type: Discussion paper. Aim: to describe genomic foundations and crosswalk existing genomic nursing competencies to an oncology nursing context to support genomics-informed cancer care. | Integrate genomics into comprehensive assessment (family history, pedigree, biomarkers, environmental and behavioural risk)  Identify candidates for somatic and germline testing and select appropriate tests across the care pathway  Provide pre-test and post-test education, counselling, and emotional support, including interpretation of variants of uncertain significance  Apply ethical, cultural, legal, and equity principles to support autonomous, informed decision-making  Coordinate and refer to genetic specialists; collaborate across interprofessional teams  Tailor prevention, screening, treatment, and risk-management plans using genomic information  Educate patients and families on hereditary risk, targeted therapies, and implications for relatives  Implement and monitor genomics-informed interventions and targeted treatments  Evaluate outcomes of genomic interventions and adjust care accordingly  Engage in reflective practice and continuous professional development in cancer genomics |
| Flynn et al., 2019^30^ | Case study. Aim: to describe how a large academic clinical research hospital integrates genomics into oncology nursing practice using adapted genomic competencies and the MINC toolkit. | Define core genetics and genomics terminology relevant to oncology care  Reflect on personal values and attitudes toward genomics and their impact on care  Apply genomic concepts to prevention, screening, diagnosis, and treatment selection  Collect a three-generation family history and construct a standardized pedigree  Protect privacy and confidentiality of genetic and genomic information  Apply legal and ethical frameworks (e.g., GINA, ADA, state laws) to genomic care  Identify and use institutional genomic resources for nursing practice  Assess patient and family baseline knowledge and perceptions of genomics  Facilitate referrals for genetic and genomic evaluation and services  Address incidental genomic findings appropriately  Provide tailored genomic information and resources to patients and families |
| Forman & Schwartz, 2019^16^ | Discussion paper. Aim: to build oncology nursing skills in cancer risk assessment, including identification of hereditary cancer risk, use of risk models, and delivery of risk education and informed consent. | Collect and document a minimum adequate cancer risk pedigree across first- and second-degree relatives  Assess ethnicity, ancestry, and both maternal and paternal lineage  Record age at diagnosis, cancer type, and prior genetic test results in family members  Expand pedigrees to include third-degree relatives when indicated  Capture detailed cancer characteristics, treatments, and exposure histories  Document current age, age at death, and cause of death for relatives  Identify benign conditions and clinical features suggestive of hereditary cancer risk  Record history of preventive or risk-reducing surgeries  Update pedigrees longitudinally as family history evolves  Recognize limitations and modifiers of pedigree interpretation (e.g., small families, adoption, consanguinity, ancestry-specific risks) |
| Godino et al., 2025^35^ | Qualitative phenomenological study. Aim: to understand the perceived roles and activities of nurses employed in genetic clinics in Italy. | Demonstrate advanced, specialist genetics expertise appropriate for clinical genetics services  Apply postgraduate-level education and research competence (e.g., master’s-level genetics training, clinical genetics projects)  Challenge misconceptions by evidencing that genetic services require high-level clinical and professional capability |
| Hoopes et al., 2022^53^ | Case study. Aim: to review an example of the oncology nurse role in a clinic designed to manage hereditary cancer risk and to highlight nursing implications. | Pursue continuing education in cancer genetics and genomics  Maintain up-to-date knowledge to support precision cancer care practice  Develop collaborative partnerships with genetic counsellors  Use interprofessional resources to address patient questions accurately |
| Hoxhaj et al., 2022^47^ | Systematic literature review and Delphi study. Aim: to identify and achieve expert consensus on core cancer genomics competencies for non-genetic healthcare professionals, including nurses. Context: cancer genomics education and clinical practice (cross-setting; not service-specific). | Recognize cancers and clinical patterns suggestive of inherited genetic risk  Understand genetic contributions to cancer development, prevention, and management  Apply inheritance patterns and evidence-based risk thresholds to guide care  Collect three-generation family histories and construct standardized pedigrees  Distinguish genetic susceptibility from disease manifestation and risk uncertainty  Integrate genetic information into health promotion, screening, and treatment decisions  Communicate genetic information sensitively, ethically, and in a culturally tailored manner  Support autonomous, informed patient and family decision-making  Coordinate care, facilitate referrals, and collaborate within multidisciplinary genetics teams  Maintain critical thinking, update genomic knowledge, and advocate equitable access to services |
| Kerber & Ledbetter, 2017b^28^ | Discussion paper with illustrative case studies. Aim: to describe evolving cancer nursing roles with the implementation of genomics and genetics standards in oncology practice, with a focus on the graduate nurse. | Assess and analyse patient data, including genetic/genomic factors  Formulate diagnoses and individualized outcomes  Plan and implement evidence-based, genomics-informed care  Coordinate care and promote health and prevention  Evaluate outcomes and quality of genomic nursing practice  Practice ethically and protect patient rights  Educate patients and maintain genomic competence  Integrate research and evidence into practice  Communicate effectively and collaborate interprofessional  Demonstrate leadership, self-evaluation, and responsible resource use  Incorporate environmental health considerations into care |
| Kerber & Ledbetter, 2017a^42^ | Discussion paper with illustrative case studies. Aim: to describe how the advanced practice nurse role evolves with the implementation of genomics and genetics standards in oncology practice. | Assess genetic risk using family history, pedigrees, and clinical context  Interpret genetic findings to support differential diagnosis and risk stratification  Plan evidence-based, cost-effective, patient-centred precision care  Implement and coordinate genomic testing, referrals, and treatments collaboratively  Educate patients on genomic risk, testing options, benefits, limits, and implications  Apply prescriptive authority and precision therapies within regulatory frameworks  Evaluate outcomes of genomic-informed interventions and revise care plans  Uphold ethics, autonomy, informed consent, and equity in genomic care  Integrate genomics evidence, research, and quality improvement into practice  Communicate genomic information clearly within interprofessional teams  Lead, mentor, and collaborate to advance precision cancer care practice  Use resources responsibly and consider environmental, social, and economic genomic influences |
| Lopes-Júnior et al., 2022^52^ | Quantitative cross-sectional survey of nursing education programs. Aim: to investigate how genetics and genomics are taught in undergraduate nursing programs in Brazil and assess alignment with essential nursing genomics competencies. | Integrate genetics and genomics technologies and information into nursing practice  Critically analyse history and physical findings for genetic, genomic, and environmental risk factors  Identify individuals who may benefit from genetic or genomic information and services  Facilitate timely referrals to specialized genetic and genomic services  Apply genetics-informed health promotion and disease prevention strategies  Recognize how personal values and attitudes toward genomics influence care  Advocate for equitable access to genetic/genomic services and resources  Tailor genetic and genomic information to patients’ culture, literacy, language, and preferences |
| Mahon & Yackzan, 2022^43^ | Case study. Aim: To illustrate scope-of-practice and competency considerations for oncology nurse practitioners ordering and managing germline genetic testing. | Collect comprehensive maternal, paternal, and personal histories for genomic assessment  Construct and interpret a three-generation pedigree  Review prior germline and tumour testing results within the family  Identify hereditary cancer patterns across multiple cancer types and syndromes  Select appropriate, comprehensive gene panels based on family history  Recognize limitations and consequences of incomplete genetic testing  Anticipate clinical, familial, and financial implications of testing decisions |
| Miller & Rosenzweig, 2021^49^ | Discussion paper. Aim: to describe how oncology nurses contribute to biobanking and support precision medicine through patient consent, specimen collection, and care coordination. | Integrate clinical care with research and biobanking requirements  Facilitate and support informed consent for specimen collection  Ensure tissue specimens are collected according to patient consent  Coordinate effectively with research coordinators and clinical teams  Manage and document consent, specimen collection, and research processes  Adapt workflows to accommodate consent discussions and specimen handling timing |
| Nembaware et al., 2019^37^ | Case study. Aim: to describe the establishment of the African Genomic Medicine Training Initiative (AGMT). | Reflect on personal values and professional boundaries in genetics/genomics practice  Distinguish genetic predisposition from clinical disease expression  Integrate genetic, genomic, environmental, and family history into patient assessment  Collaborate with interdisciplinary teams to plan genetic/genomic care and referrals  Apply ethical principles to consent, privacy, disclosure, and use of genomic data  Communicate genetic information using developmentally appropriate counselling skills  Develop and deliver genomics-informed health promotion and disease prevention strategies  Incorporate evidence, clinical judgement, and patient preferences into personalized care planning  Maintain up-to-date genomic knowledge and relevant clinical resources  Use information technology and research protocols to support genomic practice and quality assurance |
| Park et al., 2024^34^ | Systematic review. Aim: to identify and appraise healthcare professionals’ learning needs and perspectives on essential information in genetic cancer care to inform targeted education and training strategies. | Support shared decision-making by assessing decisional needs, psychosocial distress, coping strategies, and patient values  Assess and manage hereditary cancer risk, including interpreting genetic test results and variants of uncertain significance  Apply cancer risk-reduction and surveillance strategies informed by genetic testing  Use and coordinate material and human resources to support families with hereditary cancer  Guide family communication, disclosure of results, family planning, and management of familial distress  Clarify and enact the nursing role within interprofessional hereditary cancer care teams |
| Percival et al., 2016^5^ | Quantitative service evaluation. Aim: To report on the establishment and evaluation of an extended clinical nurse specialist role in consenting women for BRCA testing using a mainstreaming model. | Complete formal training and certification in germline BRCA testing  Identify patients eligible for BRCA testing using defined protocols  Explain the purpose, relevance, and limitations of BRCA testing  Obtain and document informed consent for genetic testing  Interpret and explain normal, pathogenic, and uncertain BRCA results  Communicate familial implications of positive genetic findings  Address patient questions and concerns related to genetic testing  Work under supervision and seek multidisciplinary support when needed |
| Pierle & Mahon, 2019^41^ | Narrative literature review. Aim: to identify barriers to accessing genetic care, review service delivery models that enhance access to genetic counselling and describe how oncology nurses support and facilitate the genetic counselling process. | Identify risk factors for hereditary cancer syndromes  Collect and document a three-generation family pedigree  Determine and facilitate appropriate referrals to genetic risk assessment and counselling  Understand genetic testing, risk assessment, and counselling processes, including limitations  Educate patients about strengths and limits of clinical and direct-to-consumer genetic testing  Support patients and families in informed decision-making and result interpretation |
| Rahman et al., 2022^20^ | Scoping review. Aim: to identify the genetic and genomic learning needs of oncologists and oncology nurses in the context of cancer and precision medicine. | Record informed consent for genomic testing in line with governance requirements  Maintain up-to-date clinical knowledge of conditions eligible for genomic testing  Assess appropriateness and timing of genomic testing within the care pathway  Explain the purpose, process, and implications of genomic tests to patients  Apply core clinical communication skills during genomic test discussions  Address patient questions about clinical and research aspects of testing  Recognize ongoing responsibilities in post-test care and follow-up  Seek specialist support and escalate appropriately within scope of practice |
| Regan et al., 2019^75^ | Position paper. Aim: to establish the knowledge required to integrate multiple branches of omics into nursing research and support the Genomic Nursing Science Blueprint. | Knowledge and competences described  Understand core molecular biology underpinning genomics, transcriptomics, proteomics, metabolomics, epigenetics, and microbiomics  Apply the central dogma of molecular biology to interpret disease mechanisms and therapy response  Integrate omics data with environmental, behavioural, social, and microbiome influences on health  Demonstrate foundational knowledge in cellular biology, systems physiology, and microbiology  Interpret genome structure, variation, gene expression, and epigenetic regulation  Understand protein structure, function, interactions, and post-translational modifications  Apply principles of metabolomics to link genomic variation with metabolic pathways  Understand systems physiology across major organ systems in relation to omics data  Analyse host-microbe interactions and their role in health, disease, and treatment response  Apply translational bioinformatics and big data analytics to genomic and clinical datasets  Interpret sequencing technologies, gene panels, and variant results, including incidental findings  Evaluate functional effects of genetic variants using pathway and network analysis tools  Understand genomics study designs (e.g. GWAS, sequencing studies, epidemiologic designs)  Recognize methodological and ethical limitations of genomics and data-driven research  Adapt depth of omics knowledge to role, expertise level, and research or clinical responsibility  Integrate omics knowledge to support precision health and individualized treatment decisions |
| Scott et al., 2020^4^ | Quantitative, retrospective observational study. Aim: To develop and implement a nurse-led, in-house mainstreaming cancer genetics (MCG). programme for BRCA testing and reduce waiting times. | Obtain informed consent for BRCA genetic testing  Provide pre- and post-test genetic counselling to patients  Collect, construct, and interpret a three-generation family history pedigree  Interpret and communicate genetic test results accurately  Answer patient questions before and after genetic testing  Identify indications for referral to specialist clinical genetics services  Apply standardized mainstreaming cancer genetics (MCG) protocols in practice  Collaborate with clinical genetics teams in service delivery  Mentor and train other nurses in genetic testing competencies  Engage in self-directed and mentored genetics education  Maintain competency through assessment, supervision, and case documentation  Remain current with evolving genetic evidence, guidelines, and testing protocols |
| Shevach et al., 2023^33^ | Quantitative, retrospective observational study. Aim: to evaluate the impact of an on-site nurse-led cancer genetics service on germline genetic testing completion, with a focus on racial equity. Setting: Veterans Affairs Medical Center oncology clinic. | Perform genetic risk assessment  Provide genetic counselling  Facilitate genetic testing  Interpret genetic test results  Manage clinical care based on genetic findings  Address ethical, legal, and social implications of genetics in care |
