# Supplementary material for: Nursing roles, competencies, and education in precision oncology: a scoping review
Source: eClinicalMedicine. 2026 Jul 21;98:104080. doi: 10.1016/j.eclinm.2026.104080 (PMC13396616; doi:10.1016/j.eclinm.2026.104080)
Supplement: Appendix 5 [file mmc5.docx]

**Appendix 5: Source Document Characteristics: Peer-Reviewed Education and Training Programmes for Nurses in Precision Cancer Care**

| **Study Author, year** | **Key Characteristics** | **Education Programme Content** |
| --- | --- | --- |
| Barbato et al., 2019^60^ | Discussion Paper. Aim: to propose a structured curriculum for integrating genetics and genomics into PhD nursing education and prepare nurse scientists for genomic research. | Genomic Risk Assessment and Stratification: DNA variation and Mendelian inheritance; Chromosomal abnormalities and nondisjunction; Copy number variants and human disease; Karyotyping interpretation; Sanger sequencing interpretation; Whole-exome sequencing interpretation; Whole-genome sequencing interpretation  Genomic Communication and Shared Decision-Making: Foundational genomic concepts for interpretation of results; Understanding genetic and genomic research reports.  Precision Cancer Care Pathway Coordination and Clinical Navigation: Sample collection, handling, and storage; DNA and RNA extraction and purification; Spectrophotometric analysis; Polymerase chain reaction (quantitative and endpoint); Gel electrophoresis and blotting; Proteomic measurement (ELISA).  Interprofessional Collaboration and Genomic Service Integration: Integration of genetics and genomics into nursing research design; Interpretation of genomic laboratory outputs.  Professional Governance, Quality Assurance, and Advanced Practice Roles: Cell biology foundations (cell structure, function, cell cycle); Central dogma (DNA, RNA, protein structure and function); Molecular genetics and cytogenetics foundations. |
| Bokkers et al., 2022^3^ | Quasi-experimental observational study. Aim: to assess healthcare professionals’ attitudes, knowledge, self-efficacy, and feasibility of integrating mainstream germline genetic testing for women with ovarian cancer | Genomic Risk Assessment and Stratification: hereditary vs environmental cancer mechanisms; germline vs somatic variants; blood vs tumour testing; diagnostic vs predictive testing; BRCA eligibility criteria; variant prevalence by histologic subtype; inheritance patterns; cancer risk profiles; family screening recommendations; future gene panel expansion.  Genomic Communication and Shared Decision-Making: timing of genetic testing discussions; core discussion elements; possible test outcomes and implications; family risk implications; emotional and psychosocial impacts; patient and family support needs.  Precision Cancer Care Pathway Coordination and Clinical Navigation: mainstreaming genetic testing workflows; role of genetic testing in treatment decision-making; PARP inhibitor relevance; testing procedures and result pathways; integration of tumour testing into care pathways.  Interprofessional Collaboration and Genomic Service Integration: roles of clinical geneticists, gynaecologic oncologists, and social workers; multidisciplinary consultation models; collaboration across oncology and genetics services.  Professional Governance, Quality Assurance, and Advanced Practice Roles: foundational cancer genetics knowledge; rationale for mainstreaming workflows; informed discussion standards; awareness of evolving testing practices and service models. |
| Bracci et al., 2020^45^ | Quantitative prospective observational study. Aim: to evaluate whether a nurse-led, guideline-based semi-structured interview improves identification of cancer patients eligible for genetic counselling and assesses patient interest in counselling. | Genomic Risk Assessment and Stratification: Lynch syndrome (HNPCC); hereditary breast and ovarian cancer (HBOC) syndrome; hereditary cancer risk features.  Genomic Communication and Shared Decision-Making: patient approach in oncology; explanation of genetic risk questions; patient-facing communication strategies.  Precision Cancer Care Pathway Coordination and Clinical Navigation: structured cancer risk assessment interviews; evaluation of patient-reported information.  Interprofessional Collaboration and Genomic Service Integration: genetic counsellor-led training; nurse-genetic counsellor role alignment.  Professional Governance, Quality Assurance, and Advanced Practice Roles: nurse training in guideline-informed hereditary cancer assessment; competency development in patient evaluation. |
| Carpenter-Clawson et al., 2023^51^ | Mixed methods study. Aim: to identify genomic competencies needed by nurses and midwives for genomic mainstreaming in the NHS and to inform a tailored education model. | Genomic Risk Assessment and Stratification: fundamentals of genetics and genomics; core genomic concepts; practice-relevant genetic conditions.  Genomic Communication and Shared Decision-Making: tailored genomic communication; informed decision-making; individual autonomy and voluntary action; communication with patients and families.  Precision Cancer Care Pathway Coordination and Clinical Navigation: application of genomics in clinical practice; translation of genomic knowledge to care contexts.  Interprofessional Collaboration and Genomic Service Integration: genomic communication with peers; shared genomic understanding across professional roles.  Professional Governance, Quality Assurance, and Advanced Practice Roles: genomic literacy; rights-based approaches to genomic care; progressive competency development in genetics and genomics. |
| Ceylan et al., 2025^54^ | Quasi-experimental study. Aim: to evaluate the impact of a WhatsApp-based educational intervention on nurses’ genetic knowledge and awareness. | Genomic Risk Assessment and Stratification: DNA, chromosomes, genes; heredity concepts; common chromosomal disorders; pedigree analysis.  Genomic Communication and Shared Decision-Making: genetic counselling principles; prenatal diagnosis context.  Precision Cancer Care Pathway Coordination and Clinical Navigation: genetic diseases overview relevant to testing pathways.  Interprofessional Collaboration and Genomic Service Integration: roles within genetic counselling services.  Professional Governance, Quality Assurance, and Advanced Practice Roles: foundational genomic literacy. |
| Clary-Muronda & Smith, 2024^40^ | Case study. Aim: to discuss the planning, delivery, and evaluation of an undergraduate nursing genetics course delivered by an interprofessional faculty team. | Genomic Risk Assessment and Stratification: genetic disorders and cancer; genetic testing; genomes, chromosomes, and inheritance; pedigree analysis; DNA replication, mutations, structure, and function.  Genomic Communication and Shared Decision-Making: pedigree interpretation for familial risk.  Precision Cancer Care Pathway Coordination and Clinical Navigation: newborn screening in human developmental genetics.  Interprofessional Collaboration and Genomic Service Integration: —  Professional Governance, Quality Assurance, and Advanced Practice Roles: — |
| Dewell et al., 2024^38^ | Discussion paper. Aim: to map the 2021 AACN Essentials to the ANA Essentials of Genomic Nursing and provide exemplar learning outcomes, content, and clinical vignettes to support integration of genomics into undergraduate nursing curricula. | Genomic Risk Assessment and Stratification: comprehensive family history; pedigree construction; genomic red flags; genetic-environmental contributions to complex conditions.  Genomic Communication and Shared Decision-Making: patient education on genomic risk and testing implications.  Precision Cancer Care Pathway Coordination and Clinical Navigation: genomics-informed care planning; pharmacogenomic dosing guidelines (e.g. CPIC).  Interprofessional Collaboration and Genomic Service Integration: precision health concepts linking genomics, environment, and treatment decisions.  Professional Governance, Quality Assurance, and Advanced Practice Roles: foundational genomics; epigenomics; pharmacogenomics; genomics-informed medication safety. |
| Diez de Los Rios de la Serna et al., 2022^66^ | Delphi consensus study Aim: to reach multidisciplinary consensus on core topics and competencies required for cancer nurse education in genetics, health behaviours, and cancer prevention. | Genomic Risk Assessment and Stratification: cancer determinants; common cancer-related mutations and syndromes; genetic processes; risk estimation instruments; comprehensive personal-familial-environmental history; identification of hereditary cancer predisposition; surveillance risk factors.  Genomic Communication and Shared Decision-Making: effective risk communication strategies; tailored consultation approaches; family risk communication; emotional and psychological support; beliefs and values in risk perception; family planning and fertility considerations.  Precision Cancer Care Pathway Coordination and Clinical Navigation: genetics in cancer treatment decision-making; follow-up and long-term support roles; surveillance challenges; prevention-oriented care pathways.  Interprofessional Collaboration and Genomic Service Integration: health promotion and education roles; community and family-focused support; coordination of follow-up for high-risk individuals and families.  Professional Governance, Quality Assurance, and Advanced Practice Roles: modifiable cancer risk factors; social and behavioural determinants of genetic susceptibility; cancer risk reduction recommendations; health belief and behaviour change theories; psychosocial impacts of cancer on patients and families. |
| Dodson, 2018^63^ | Quantitative; evaluation of an education programme. Aim: to assess the efficacy of an interactive online continuing education module in improving oncology nurses’ knowledge of pharmacogenomics in clinical practice. Education programme details: 45-minute online asynchronous interactive online module. | Genomic Risk Assessment and Stratification: Foundational genomics and pharmacogenomics concepts; biomarker-based patient stratification (e.g. HER2).  Genomic communication and decisional support: interpretation of gene-drug interactions relevant to treatment decisions.  Precision Cancer Care Pathway Coordination and Clinical Navigation: pharmacogenetic testing informing drug selection, dosing, and targeted cancer therapy.  Interprofessional Collaboration and Genomic Service Integration: alignment of pharmacogenomic results with oncology treatment planning.  Professional Governance, Quality Assurance, and Advanced Practice Roles: clinical application of validated pharmacogenetic examples to support safe, evidence-informed practice. |
| Edelman et al., 2019^65^ | Quantitative; evaluation of an education programme. Aim: to evaluate engagement with and effectiveness of interactive, case-based online education modules on somatic tumour testing in precision cancer care. Education programme details: Two learning modules. | Genomic Risk Assessment and Stratification: candidate selection for somatic panel testing; genomic variant detection; tumour heterogeneity; tumour genomic evolution; incidental germline variant identification.  Genomic Communication and Shared Decision-Making: interpretation of actionable versus uncertain variants; variants of uncertain significance; contextualized result meaning for individual patients.  Precision Cancer Care Pathway Coordination and Clinical Navigation: application of somatic panel results to treatment selection; targeted therapy options beyond standard of care; clinical trial eligibility; off-label therapy considerations; longitudinal testing and re-biopsy needs.  Interprofessional Collaboration and Genomic Service Integration: molecular tumour boards; laboratory comparison and test selection; integration of tumour testing into oncology workflows.  Professional Governance, Quality Assurance, and Advanced Practice Roles: limitations of clinical actionability evidence; methodological differences in somatic testing platforms; insurance coverage constraints; quality appraisal of test reports and laboratories. |
| Flynn et al., 2019^30^ | Case study. Aim: to describe how a large academic clinical research hospital integrates genomics into oncology nursing practice using adapted genomic competencies. | Genomic Risk Assessment and Stratification: inheritance patterns; genetic risk assessment; pedigree construction; mutations and disease association; epigenetics; incidental findings.  Genomic Communication and Shared Decision-Making: pedigree-based family risk context; genetic testing concepts; interpretation of inherited conditions.  Precision Cancer Care Pathway Coordination and Clinical Navigation: pharmacogenomics; gene therapy; molecular mechanisms of chemotherapy, biotherapy, and immunotherapy; cellular therapies (CAR-T).  Interprofessional Collaboration and Genomic Service Integration: integration of genomics into nursing practice; genetics and genomics resources; genomics in current clinical practice.  Professional Governance, Quality Assurance, and Advanced Practice Roles: foundational genomics knowledge; research protocol application; evolving genomic technologies; professional capability in genomics and precision cancer care. |
| Kronk et al., 2023^69^ | Case study. Aim: to describe and evaluate an NHGRI-funded, self-paced online professional development course designed to improve genetics and genomics literacy among doctoral-level nurses. Education programme: Online asynchronous professional development course. | Genomic Risk Assessment and Stratification: risk assessment and interpretation; genetic basis of disease; health risk assessment; epigenetics; genetic primer/basics of genetics and genomics.  Genomic Communication and Shared Decision-Making: genetic education; genetic counselling; genetic testing; results interpretation.  Precision Cancer Care Pathway Coordination and Clinical Navigation: personalized health care; clinical management.  Interprofessional Collaboration and Genomic Service Integration: genomics literacy for doctoral-level nurses and faculty.  Professional Governance, Quality Assurance, and Advanced Practice Roles: ethical implications of genetic and genomic advancements. |
| Nembaware et al., 2019^37^ | Case study. Aim: to describe the establishment of the African Genomic Medicine Training Initiative (AGMT). | Genomic Risk Assessment and Stratification: patterns of genetic inheritance; monogenic disorders; genes, genome structure and function; molecular pathology of cancer; cancer diagnosis and screening.  Genomic Communication and Shared Decision-Making: genetic counselling; basic genetic counselling skills.  Precision Cancer Care Pathway Coordination and Clinical Navigation: molecular pathology applications in treatment; pharmacogenetics and pharmacogenomics in cancer care.  Interprofessional Collaboration and Genomic Service Integration: molecular diagnostics; bioinformatics techniques.  Professional Governance, Quality Assurance, and Advanced Practice Roles: foundational genomics knowledge for nursing practice; genomics capacity building in low-resource settings. |
| Nightingale et al., 2025^67^ | Mixed methods evaluation of an education programme Aim: to evaluate whether the Master’s in Genomic Medicine achieves its purpose of developing health care professionals’ genomic knowledge and ability to embed genomics in professional practice, and to identify facilitators and barriers to impact. | Genomic Risk Assessment and Stratification: fundamentals of human genetics/genomics; genomics of common and rare inherited diseases.  Genomic Communication and Shared Decision-Making: counselling skills for genomic medicine.  Precision Cancer Care Pathway Coordination and Clinical Navigation: molecular pathology of cancer; genomics applications in cancer diagnosis, treatment, monitoring; pharmacogenomics and stratified health care.  Interprofessional Collaboration and Genomic Service Integration: counselling skills within genomic medicine services.  Professional Governance, Quality Assurance, and Advanced Practice Roles: omics techniques/technologies; bioinformatics; interpretation; data quality assurance. |
| Percival et al., 2016^5^ | Quantitative service evaluation. Aim: To report on the establishment and evaluation of an extended clinical nurse specialist role in consenting women for BRCA testing using a mainstreaming model. | Genomic Risk Assessment and Stratification: patient identification protocols; relevance of BRCA testing; interpretation of normal, pathogenic, and variant-of-uncertain-significance results.  Genomic Communication and Shared Decision-Making: implications of BRCA results for patients; familial risk implications; frequently asked questions.  Precision Cancer Care Pathway Coordination and Clinical Navigation: BRCA testing pathways; result follow-up processes.  Interprofessional Collaboration and Genomic Service Integration: genetics team-led education resources.  Professional Governance, Quality Assurance, and Advanced Practice Roles: standardized training materials; competency-focused learning resources. |
| Scott et al., 2020^4^ | Quantitative, retrospective observational study. Aim: To develop and implement a nurse-led, in-house mainstreaming cancer genetics (MCG). programme for BRCA testing and reduce waiting times. Nurse training/preparation: Structured genetics training package including online modules, face-to-face genetics education, mentorship, competency assessment, and ongoing CPD with clinical genetics services. | Genomic Risk Assessment and Stratification: BRCA1/BRCA2 genetics; three-generation family history pedigree; hereditary breast cancer risk features; recognition of other cancer syndromes.  Genomic Communication and Shared Decision-Making: genetic consent processes; pre- and post-test counselling content; patient question handling; disclosure of genetic test results.  Precision Cancer Care Pathway Coordination and Clinical Navigation: BRCA testing workflow; patient observations in practice; referral pathways to clinical genetics services; management of complex or atypical results.  Interprofessional Collaboration and Genomic Service Integration: mentorship by lead breast clinical nurse specialists; training by clinical genetics specialists; nurse-genetics service collaboration.  Professional Governance, Quality Assurance, and Advanced Practice Roles: foundational genetics education; formal consent training; competency-based learning packages; supervised practice and specialist training sessions. |
| Silva et al., 2025^64^ | Documentary curriculum analysis. Aim: to explore how genomic knowledge is taught in Portuguese undergraduate nursing education at a national level and assess alignment with established genomic topics in the literature. | Genomic Risk Assessment and Stratification: Mendelian inheritance patterns; chromosomes, genes, DNA relationships; causes of genetic disease; inherited disorders vs genetic susceptibility; family history; risk assessment.  Genomic Communication and Shared Decision-Making: communication of genetic information to families; psychosocial impact of genetic conditions on families.  Precision Cancer Care Pathway Coordination and Clinical Navigation: genetic testing types and implications; preventive actions; treatment implications.  Interprofessional Collaboration and Genomic Service Integration: family-based genetic risk contexts.  Professional Governance, Quality Assurance, and Advanced Practice Roles: foundational molecular biology concepts; DNA transcription to protein structure. |
| Smania et al., 2022^68^ | Quality improvement initiative. Aim: to increase genetics/genomics content in an undergraduate nursing curriculum by improving faculty knowledge and confidence in genetic/genomic competencies. Programme delivery: Faculty-focused, face-to-face professional development workshops integrated into curriculum review and revision | Genomic Risk Assessment and Stratification: genetic assessment; basic genetics; genetic conditions across the lifespan; genomics in breast cancer; family testing.  Genomic Communication and Shared Decision-Making: family testing and treatment considerations.  Precision Cancer Care Pathway Coordination and Clinical Navigation: genetic testing options; pharmacogenomics; nutrigenomics.  Interprofessional Collaboration and Genomic Service Integration: strategies for integrating genetics into curricula.  Professional Governance, Quality Assurance, and Advanced Practice Roles: foundational genetics knowledge; genetics in children and adults; genomics literacy across practice contexts. |
