# Supplementary material for: Nursing roles, competencies, and education in precision oncology: a scoping review
Source: eClinicalMedicine. 2026 Jul 21;98:104080. doi: 10.1016/j.eclinm.2026.104080 (PMC13396616; doi:10.1016/j.eclinm.2026.104080)
Supplement: Appendix 6 [file mmc6.docx]

**Appendix 6: Source Document Characteristics: Grey Literature Education and Training Programmes for Nurses in Precision Cancer Care**

| Reference # | Programme Overview & Institutional Context | Target Audience & Entry Requirements | Qualification, Award & Academic Weight | Learning Outcomes |
| --- | --- | --- | --- | --- |
| 1 | Continent: Africa Country: Ghana Type of Institution: University Level of Qualification: 5‑day CPD Certificate Programme Title / Subject: Allied Health Genomics (short course) Accreditation / Professional Recognition: Not stated Mode of Delivery: Not stated | Target Audience: Allied health professionals and other healthcare personnel including physicians, nurses, midwives, pharmacists, and health scientists. Entry Requirements: No specific entry requirements stated. | Award on Completion: Certificate in Allied Health Genomics Credits: Not stated Programme Length: 5 days Academic vs CPD: Short course Stackable / Pathway: Not stated | Genetic literacy, genomic testing, large‑scale genomics projects, personalised medicine policy, ethical‑legal‑social issues in genomics, and genomics applications across allied health specialties. |
| 2 | Continent: Africa Country: Ghana Type of Institution: University Level of Qualification: MSc Programme Title / Subject: MSc. in Genetic Counselling  Accreditation / Professional Recognition: Accredited by the Ghana Tertiary Education Commission Mode of Delivery: In person | Target Audience: Not stated Entry Requirements: No specific entry requirements stated. | Award on Completion: MSc Credits: 60 credits Programme Length: 2 years (plus one-year mandatory internship under the supervision of the Allied Health Professions Council) Academic vs CPD: Academic programme Stackable / Pathway: Leads to eligibility for registration as a genetic counsellor following a 1‑year internship | Upon completion participants should have the ability to evaluate clients’ eligibility for genetic testing, make referrals for testing, interpret genetic reports, expertly communicate genetic information. Provide expert genetic counselling suitable to each individual’s needs. |
| 3 | Continent: Africa Country: Nigeria Type of Institution: University Level of Qualification: MSc Programme Title / Subject: Cell Biology & Genetics (Environmental Biology) Accreditation / Professional Recognition: Not stated Mode of Delivery: Not stated | Target Audience: Not stated Entry Requirements: Applicants hold a minimum Second-Class Lower Bachelor’s degree or equivalent in biological or health‑related science fields; or a medical degree; or a relevant postgraduate diploma with minimum CGPA of 3.00 and an honours degree in a related science discipline. | Award on Completion: MSc Credits: Not stated Programme Length: Not stated Academic vs CPD: Academic programme Stackable / Pathway: Not stated | Not stated |
| 4 | Continent: Africa Country: Nigeria Type of Institution: University Level of Qualification: MSc Programme Title / Subject: Cell Biology & Genetics (Genetics) Accreditation / Professional Recognition: Not stated Mode of Delivery: Not stated | Target Audience: Not stated Entry Requirements: Applicants hold a Bachelor’s degree with at least Second Class Lower Division in biological science fields; or a degree in Medicine, Anatomy, or Physiology; or a relevant postgraduate diploma with minimum CGPA of 3.00 and an honours degree in a related science; and may be required to complete a written and/or oral selection interview or examination. | Award on Completion: MSc Credits: Not stated Programme Length: Twelve calendar months Academic vs CPD: Academic programme Stackable / Pathway: Not stated | Not stated |
| 5 | Continent: Africa Country: Nigeria Type of Institution: University Level of Qualification: Post Graduate Diploma Programme Title / Subject: Cell Biology & Genetics (Forensic Biology) Accreditation / Professional Recognition: Not stated Mode of Delivery: Not stated | Target Audience: Individuals with biological, medical, biomedical, paramedical, or education‑related backgrounds who engage in activities requiring knowledge of forensic biology or genetic counselling; examples include police personnel, clergy, medical and legal practitioners, security personnel, and other related roles. Entry Requirements: Applicants hold a Bachelor’s degree or equivalent with at least a third class in biological, medical, biomedical, paramedical, or education‑related fields; candidates may require forensic biology or genetic counselling knowledge; applicants who plan to progress to an MSc must achieve a minimum CGPA of 3.00 in the PGD; a selection process may also be required. | Award on Completion: Post Graduate Diploma Credits: Not stated Programme Length: Not stated Academic vs CPD: Academic programme Stackable / Pathway: Progression to MSc possible with a minimum CGPA of 3.00 | Not stated |
| 6 | Continent: Africa Country: Nigeria Type of Institution: University Level of Qualification: Post Graduate Diploma Programme Title / Subject: Cell Biology & Genetics (Genetic Counselling) Accreditation / Professional Recognition: Not stated Mode of Delivery: Not stated | Target Audience: Individuals with biological, medical, biomedical, paramedical, or education‑related backgrounds who require knowledge of genetic counselling or forensic biology, including police personnel, clergy, medical practitioners, legal practitioners, security personnel, and other related personnel. Entry Requirements: Applicants hold a Bachelor’s degree or equivalent with at least a third class in biological, medical, biomedical, paramedical, or education‑related disciplines; candidates seeking progression to an MSc require a minimum CGPA of 3.00 in the PGD; applicants may also be required to complete a selection process. | Award on Completion: Post Graduate Diploma Credits: Not stated Programme Length: One year (two semesters, full time) Academic vs CPD: Academic programme Stackable / Pathway: Progression to MSc possible with minimum 3.00 CGPA | Not stated |
| 7 | Continent: Africa Country: Nigeria Type of Institution: University Level of Qualification: MSc Programme Title / Subject: Molecular Biology & Genomics Accreditation / Professional Recognition: Programme is accredited by AQAS according to AQAS criteria; accreditation valid until 30 September 2025. Mode of Delivery: In person | Target Audience: Not stated Entry Requirements: Applicants meet Nigerian O‑Level requirements including English, Mathematics, and Biology; hold a bachelor’s degree in microbiology, biochemistry, molecular biology and genetics, medicine, veterinary medicine, or other life science disciplines with minimum second‑class lower; Higher National Diploma or Postgraduate Diploma with CGPA 3.0/5.0 may also be considered. | Award on Completion: MSc Credits: Not stated Programme Length: 4 semesters Academic vs CPD: Academic programme Stackable / Pathway: Not stated | Programme aims to impart knowledge of genetic tools to address infectious disease challenges; curriculum includes research methodology, statistical methods, management and entrepreneurship, advanced parasitology, advanced bacteriology, antimicrobial chemotherapy, and applied genomics through a 12‑week internship. |
| 8 | Continent: Africa Country: South Africa Type of Institution: University Level of Qualification: MSc (Medicine) Programme Title / Subject: Genomic Medicine Accreditation / Professional Recognition: Not stated Mode of Delivery: In person | Target Audience: Not stated Entry Requirements: A final‑year grade average of 65% in an appropriate science degree. | Award on Completion: MSc Credits: Not stated Programme Length: 1 year Academic vs CPD: Academic programme Stackable / Pathway: Not stated | Foundational knowledge in genomic data interpretation; fundamentals of human genetics and genomics; omics techniques and applications; genomics in medicine; research experience through a genomics‑related project. |
| 9 | Continent: Africa Country: South Africa Type of Institution: University Level of Qualification: MSc (Medicine) Programme Title / Subject: Genetic Counselling Accreditation / Professional Recognition: Not stated Mode of Delivery: In person | Target Audience: Not stated Entry Requirements: Preferable BSc (Honours) in a life sciences field with minimum 65%; other Honours degrees may be considered; entry exam may be required for those without a Genetics major; applicants must complete a pre‑application process; questionnaire, face‑to‑face selection, and referee reports required for shortlisted candidates. | Award on Completion: MSc Credits: Not stated Programme Length: 1 year (Plus mandatory 2‑year internship in an accredited training institute) Academic vs CPD: Academic programme Stackable / Pathway: Leads to eligibility for registration as a genetic counsellor following a 2‑year internship. | Foundational concepts in medical genetics; theories and practices of genetic counselling; structured clinical exposure; supervised counselling practice; research project; preparation for professional genetic counselling practice and internship. |
| 10 | Continent: Africa Country: South Africa Type of Institution: University Level of Qualification: MSc (Medicine) Programme Title / Subject: Genetic Counselling Accreditation / Professional Recognition: Not stated Mode of Delivery: In person | Target Audience: Health‑care professionals seeking stand‑alone NQF level 9 courses in Medical Genetics and Genetic Counselling; individuals interested in a career as a genetic counsellor. Entry Requirements: NQF level 8 qualification acceptable to the Health Science Faculty in fields such as biological science, nursing, psychology or social work; knowledge of genetics essential; entrance examination may be required; selection process includes questionnaire, interview, and referee reports; candidates must complete a pre‑application process. | Award on Completion: MSc Credits: 24 credits Programme Length: 2 years Academic vs CPD: Academic programme Stackable / Pathway: Stand‑alone NQF9 courses may contribute to the full‑time degree within 4 years; degree leads to eligibility for HPCSA internship (not a programme requirement itself) | Epidemiology, aetiology, phenotypes, medical management and investigation of genetic disorders; application of scientific and clinical genetic knowledge; interpretation of genetic investigations; family and individual risk assessment; sourcing genetic data; understanding developing genetic technologies; theoretical and applied learning in Medical Genetics and Genetic Counselling. |
| 11 | Continent: Africa Country: South Africa Type of Institution: Government‑sponsored body Level of Qualification: Short Course (10 days) Programme Title / Subject: African Genomics Accreditation / Professional Recognition: Not stated Mode of Delivery: In person | Target Audience: Researchers and clinical professionals engaged in genomics, bioinformatics or related fields, including advanced PhD students, postdoctoral researchers, clinicians, genetic counsellors and laboratory scientists. Entry Requirements: Applicant is based in Africa, actively involved in genomics or bioinformatics research or its clinical application, at late‑stage PhD or early‑mid career level, holds AfSHG membership, and demonstrates motivation, leadership potential, and engagement in capacity building. | Award on Completion: Not stated Credits: Not stated Programme Length: 10 days Academic vs CPD: Short course Stackable / Pathway: Not stated | Foundational genomics and bioinformatics concepts; practical genomic data analysis; ethical and equitable genomics research considerations; evaluation of genomics technology potential and limitations; leadership, collaboration and reflective professional practice supporting African genomics capacity. |
| 12 | Continent: Africa Country: Uganda Type of Institution: University Level of Qualification: MSc Programme Title / Subject: Bioinformatics Accreditation / Professional Recognition: Not stated Mode of Delivery: Not stated | Target Audience: Not stated Entry Requirements: Bachelor’s degree in life sciences or physical sciences, or Postgraduate Diploma in Bioinformatics; must also meet general admission requirements of the university. | Award on Completion: MSc Credits: Not stated Programme Length: 2 years Academic vs CPD: Academic programme Stackable / Pathway: Not stated | Not stated |
| 13 | Continent: Africa Country: Zimbabwe Type of Institution: University Level of Qualification: MSc Programme Title / Subject: Bioinformatics & Genomics Accreditation / Professional Recognition: Not stated Mode of Delivery: Not stated | Target Audience: Not stated Entry Requirements: O‑Level passes in English and Mathematics; two A‑Level passes in Biology, Physics, Chemistry, Mathematics, Computer Science, or Geography; one A‑Level must be an experimental science; Honours‑level relevant undergraduate degree. | Award on Completion: MSc Credits: Not stated Programme Length: 2 years (full time) Academic vs CPD: Academic programme Stackable / Pathway: Not stated | Application of informatics and mathematics to acquisition, processing, analysis, storage, and archiving of genomics, transcriptomics, metabolomics, metagenomics, and proteomics datasets; relevance of datasets to cancer genomics, human health, pharmacogenomics, crop and animal improvement, environmental and industrial microbial genomics, population genomics, and computer‑aided drug design. |
| 14 | Continent: Africa Country: Zimbabwe Type of Institution: University Level of Qualification: MSc Programme Title / Subject: Genomics & Precision Medicine Accreditation / Professional Recognition: Not stated Mode of Delivery: On campus | Target Audience: Not stated Entry Requirements: Programme uses three categories—normal, mature, and special entry—but no descriptions are provided. | Award on Completion: MSc Credits: Not stated Programme Length: 2 years Academic vs CPD: Academic programme Stackable / Pathway: Not stated | Advanced knowledge and skills in genomic and precision medicine technologies; capability to design and communicate genomic solutions; ethical and safety considerations in genomic data use; development of innovative diagnostic and therapeutic applications; preparation to influence genomics policy and strategy; participation in national and international research collaboration. |
| 15 | Continent: Africa (Pan‑Africa: 11 countries) Country: Not stated Type of Institution: Voluntary organisation Level of Qualification: Blended CPD Certificate Programme Title / Subject: African Genomic Medicine Training Accreditation / Professional Recognition: Not stated Mode of Delivery: Blended | Target Audience: Nurses, medical doctors, pathologists, medical laboratory scientists, medical laboratory technicians, and other healthcare professionals in Africa with no prior genomic medicine experience. Entry Requirements: Not stated | Award on Completion: Not stated Credits: Not stated Programme Length: Not stated Academic vs CPD: Short course Stackable / Pathway: Not stated | Genomic and genetics education for health professionals; development of competencies in genomic medicine; application of genomic medicine content to diverse clinical roles through problem‑based learning; delivery of basic genetic counselling; ethical genomics research; interpretation of genomic tests and understanding analytic and clinical validity; genomic test quality control; strengths and weaknesses of omics approaches. |
| 16 | Continent: Europe Country: UK Type of Institution: University Level of Qualification: MSc Programme Title / Subject: Genomic Medicine (Distance/Online) Accreditation / Professional Recognition: Not stated Mode of Delivery: Online | Target Audience: Healthcare professionals Entry Requirements: A 2:2 or above at undergraduate level in a relevant subject. | Award on Completion: MSc Credits: Not stated Programme Length: 2 years part-time Academic vs CPD: Academic programme Stackable / Pathway: Available as PGDip and PGCert | Fundamentals of human genomics, omics techniques and application to genomic medicine, pharmacogenomics, bioinformatics. Application of genomic medicine to cancer diagnosis and infectious disease. |
| 17 | Continent: Europe Country: UK Type of Institution: University Level of Qualification: Master’s / PGDip / PGCert Programme Title / Subject: Genomic Medicine (Online) Accreditation / Professional Recognition: Developed by Health Education England (HEE); no explicit accreditation stated Mode of Delivery: Online | Target Audience: Clinical practitioners, diagnostic service providers, scientists, researchers, and individuals seeking specialisation in genomic medicine pathways. Entry Requirements: Normally a minimum 2:2 Honours degree (or equivalent) in a relevant discipline; relevant clinical or professional experience may be considered; personal statement required. | Award on Completion: MSc / Postgraduate Diploma / Postgraduate Certificate Credits: MSc = 200 credits; PGDip = 120 credits; PGCert = 60 credits Programme Length: MSc: 1 year full‑time / 2 or 3 years part‑time; PGDip: 2 years part‑time; PGCert: 1 year part‑time Academic vs CPD: Academic programme Stackable / Pathway: Modules may be combined across MSc, PGDip, PGCert; stand‑alone modules available | Core Learning Outcomes: Understanding of human genetics, genomic variation, and mechanisms of disease; Application of omics technologies across rare diseases, cancer, and infectious disease; Bioinformatics, sequence analysis, variant annotation, and data quality assurance; Pharmacogenomics and stratified healthcare; ethical, legal and social issue in applied genomics; counselling skills for genomics; development of research skills through data-based or literature-based dissertation. |
| 18 | Continent: Europe Country: UK Type of Institution: University Level of Qualification: MSc / PGDip / PGCert Programme Title/Subject: Genomic Medicine Accreditation / Professional Recognition: Not stated Mode of Delivery: In person | Target Audience: Healthcare professionals including nurses, doctors, pharmacists, midwives; biomedical scientists; biotechnology graduates; researchers; NHS staff; individuals retraining into genomics; biomedical or healthcare professionals seeking flexible online study. Entry Requirements: Minimum 2:2 bioscience degree with sufficient genetics content; healthcare graduates require a pass; alternative qualifications or experience considered; medical students with 360 credits including 120 at Level 6 eligible; international qualifications accepted. | Award on Completion: MSc or PGDip or PGCert Credits: MSc 180; PGDip 120; PGCert 60 Programme Length: MSc full‑time 1 year or part‑time 2–3 years; PGDip full‑time 1 year or part‑time 2 years; PGCert part‑time 1 year; Online PGCert part‑time 1 year Academic vs CPD: Academic programme with modules available as CPD Stackable / Pathway: Stackable from PGCert to PGDip to MSc | Learners understand human genetics and variation, apply genomic and omics technologies, interpret genomic data, use bioinformatics for data analysis, examine genomics across cancer, rare diseases, infectious diseases, cardiovascular and neurological disorders, evaluate ethical and legal issues, and apply communication and counselling principles in genomic contexts. |
| 19 | Continent: Europe Country: UK Type of Institution: University Level of Qualification: MSc / Postgraduate Diploma Programme Title/Subject: Genomic Medicine & Healthcare (Online) Accreditation / Professional Recognition: Not stated Mode of Delivery: Online | Target Audience: Doctors, nurses, pharmacists, allied health professionals, genetic counsellors, medical specialists, primary and secondary care professionals, and applicants with relevant healthcare degrees or experience. Entry Requirements: First degree or equivalent in a relevant healthcare field; registered healthcare professionals without formal qualifications considered individually; may require submission of assessed work; English language requirement (IELTS 6.0 or equivalent for non‑native speakers); basic IT skills required; CV, degree certificate, reference, personal statement, and proof of English competency required. | Award on Completion: MSc or Postgraduate Diploma Credits: MSc 180 credits; PGDip 120 credits; alternative international MSc route 120 credits Programme Length: MSc 24 months part time; PGDip 12 months part time; international MSc structured over one calendar year Academic vs CPD: Academic programme Stackable / Pathway: PGDip can convert to MSc by completing the second year | Understanding of medical genomics and multi-omics; evaluation of genomic evidence; application of genomic principles to personalised care; appraisal of ethical, legal, societal and cultural aspects; integration of genomic data into clinical decision‑making; development of research and critical appraisal capabilities; leadership and management within genomic healthcare contexts. |
| 20 | Continent: Europe Country: UK Type of Institution: University Level of Qualification: MSc Programme Title/Subject: Genomic Medicine with Data Science Accreditation / Professional Recognition: Not stated Mode of Delivery: Online | Target Audience: Professionals and graduates in biological sciences, genetics or medicine; applicants from data science backgrounds with prior genetics or molecular biology knowledge. Entry Requirements: 2:1 bachelor degree in a relevant scientific discipline; or 2:2 with minimum two years’ relevant work experience; or 2:2 in any subject with minimum three years’ relevant work experience; individually assessed applications; IELTS 6.5 overall with no component below 6.0. | Award on Completion: MSc Credits: Not stated Programme Length: 24 months part time Academic vs CPD: Academic programme Stackable / Pathway: Not specified | Understanding of precision diagnostics and anti‑cancer drug development; use of high‑throughput technologies; analysis of genomic, proteomic and metabolic datasets; application of data science to complex and rare disease research; use of computational tools in genomic data science; application of analytical skills in precision medicine; understanding of genetic epidemiology, clinical trials and statistical learning. |
| 21 | Continent: Europe Country: UK Type of Institution: University Level of Qualification: MSc Programme Title/Subject: Bioinformatics & Computational Genomics Accreditation / Professional Recognition: Not stated Mode of Delivery: In person | Target Audience: Individuals with backgrounds in natural sciences, mathematics, computer science, medical or life sciences, including those holding medical or dental degrees; applicants with prior study in genetics, molecular biology, biomedical science, chemistry, mathematics, statistics, computing or informatics. Entry Requirements: Minimum 2.2 honours degree in a relevant subject; completion of a module in specified subject areas; online aptitude test required; medical and dental degrees accepted. | Award on Completion: MSc (with PG Diploma and PG Certificate exit awards available) Credits: 180 CATS (120 taught + 60 dissertation) Programme Length: 1-year full time Academic vs CPD: Academic programme Stackable / Pathway: PGCert → PGDip → MSc pathway available | Competence in statistical programming and scientific computing; ability to analyse gene expression and multi‑omics data; understanding of genomic mechanisms in human disease; application of bioinformatics tools to complex datasets; knowledge of systems medicine, machine learning and network biology; capability in health and biomedical informatics; skills in research design, analysis and scientific communication. |
| 22 | Continent: Oceania Country: Australia Type of Institution: University Level of Qualification: MSc / PGDip / PGCert Programme Title/Subject: Diagnostic Genomics Accreditation / Professional Recognition: Eligibility to apply for Human Genetics Society of Australasia (HGSA) professional accreditation as diagnostics genomic scientist (exam component satisfied)  Mode of Delivery: Online | Target Audience: Scientists, managers, nursing professionals, medical professionals, allied health professionals, and other professionals seeking genomic literacy; individuals seeking diagnostic genomics expertise. Entry Requirements: Recognised bachelor degree completed within the last 10 years in specified biological, medical, or related disciplines; applicants with older qualifications require relevant professional work experience; English language proficiency required (IELTS 6.5 or equivalent). | Award on Completion: MSc / PGDip / PGCert Credits: 96 credit points Programme Length: 1.5 years full time; 3 years part time Academic vs CPD: Academic programme Stackable / Pathway: Articulates from Graduate Certificate and Graduate Diploma; provides pathway to MSc | Advanced knowledge of human genetics, diagnostic genetics, genetic technologies, molecular genomics, cytogenomics, cancer genomics, genome analysis and variant curation, laboratory management and quality control, ethical–legal–social aspects of genomics, computational genomics, diagnostic genomics research and dissertation skills. |
| 23 | Continent: North America Country: USA Type of Institution: University Level of Qualification: Certificate of Achievement Programme Title/Subject: Genomic Competencies for Nurses (Online) Accreditation / Professional Recognition: Accredited provider of nursing continuing professional development by the ANCC Commission on Accreditation Mode of Delivery: Online | Target Audience: Doctoral‑level nursing students, clinicians, and faculty seeking genomic training. Entry Requirements: Must be a doctoral‑level nurse (e.g., DNP, PhD, EdD). | Award on Completion: Certificate of Achievement; nursing contact hours available Credits: Not stated (contact hours: up to 24) Programme Length: Self‑paced; recommended 6–12 weeks Academic vs CPD: CPD short course Stackable / Pathway: Not specified | Genomic literacy; genetic basis of disease and health risk assessment; epigenetics and personalised healthcare; ethical and social implications of genomics; application of genomic medicine in research, teaching and clinical practice; professional leadership in genomics. |
| 24 | Continent: North America Country: USA Type of Institution: University Level of Qualification: Graduate Certificate Programme Title/Subject: Health Care Genetics & Genomics Accreditation / Professional Recognition: Not stated Mode of Delivery: Online | Target Audience: Nurses and other healthcare professionals; working HCPs seeking genomic knowledge. Entry Requirements: Baccalaureate degree in nursing or a health‑related field from a regionally accredited institution. | Award on Completion: Graduate Certificate / Certificate of Achievement Credits: 12 credits Programme Length: Not stated (online certificate; four 3‑credit courses) Academic vs CPD: Academic programme Stackable / Pathway: Not specified | Foundational and advanced human genetics and genomics; genomics–environment interactions in inherited disorders; epigenetics and pharmacogenomics; genetic testing and counselling; application of genomics in clinical practice; practicum‑based genomic skill development. |
| 25 | Continent: North America Country: USA Type of Institution: University Level of Qualification: Not stated Programme Title/Subject: Genomic Medicine in Clinical Practice Accreditation / Professional Recognition: Not stated Mode of Delivery: Online | Target Audience: Healthcare professionals, researchers, and medical students. Entry Requirements: None required. | Award on Completion: Certificate of Completion Credits: Not stated Programme Length: Self‑paced, always available Academic vs CPD: Short course Stackable / Pathway: Not stated | Advances in genomic medicine; family history and pedigree interpretation; genomic variation and phenotypic expression; laboratory analysis considerations; clinical genetic testing; basic genetic counselling workflow; preventive genomic screening; ethical, legal and social issues in genomic medicine. |
| 26 | Continent: North America Country: USA Type of Institution: University Level of Qualification: Graduate Certificate Programme Title/Subject: Precision Medicine Accreditation / Professional Recognition: Not stated Mode of Delivery: Online | Target Audience: Current and future healthcare professionals including doctors, nurses, PAs, pharmacists, pre‑med students, and genetic counsellors. Entry Requirements: Bachelor’s degree; proficiency in English; healthcare experience preferred; no transfer credits accepted. | Award on Completion: Graduate Certificate Credits: 9 credits Programme Length: Not stated Academic vs CPD: Short Course Stackable / Pathway: Not stated | Principles of genetics and genomics; clinical pharmacogenomics; ethical, legal and social implications of precision medicine; genomic biomarkers in cancer care; gene therapy; digital health foundations. |
| 27 | Continent: North America Country: USA Type of Institution: Medical school Level of Qualification: CNE/CME Certificate Programme Title/Subject: Advancing Care Through Genomics: Essentials for Nursing Practice Accreditation / Professional Recognition: Jointly accredited by ACCME, ACPE, and ANCC; awards AMA PRA Category 1 Credits™, ANCC contact hours Mode of Delivery: Online | Target Audience: Registered nurses; also relevant to advanced practice nurses, licensed practical nurses, pharmacists, physicians, physician assistants, students, and other healthcare team members Entry Requirements: Not stated | Award on Completion: CNE/CME Certificate Credits: 10.5 credits Programme Length: 1 day (10:36:59 content hours) Academic vs CPD: CPD Stackable / Pathway: Not stated | Fundamental genetic and genomic concepts; family medical history assessment; genomic testing methodologies; implications of genomic test results; genomics in patient‑focused health teaching and promotion; ethical, legal and social issues including privacy and confidentiality |
| 28 | Continent: Asia Country: China Type of Institution: University Level of Qualification: MSc (MSc.) Programme Title/Subject: Genetic Counselling and Nursing Accreditation / Professional Recognition: Not stated Mode of Delivery: Not stated | Target Audience: Nurses working in China Entry Requirements: Prerequisites in histology and embryology, physiology, and cell biology | Award on Completion: MSc Credits: Not stated Programme Length: Three to five years full‑time Academic vs CPD: Academic programme Stackable / Pathway: Not specified | Human genetics; ethics; advanced communication and management in nursing; nursing in genetic counselling research; thesis development and supervision |
| 29 | Continent: Asia Country: India Type of Institution: University Level of Qualification: MSc Programme Title/Subject: Clinical Genomics Accreditation / Professional Recognition: Not stated Mode of Delivery: In person | Target Audience: Students from bioscience backgrounds. Entry Requirements: BSc graduates in molecular biology, medical biotechnology, medical microbiology, microbiology, biomedical sciences, biotechnology, botany, zoology, medical genetics, biochemistry, bio‑ or health informatics, clinical research, food science and nutrition, environmental science, environmental health sciences, applied biology, applied psychology, nursing, allied health sciences, agriculture, horticulture, sericulture, forestry, or related bioscience courses with at least 60% marks. | Award on Completion: One‑year Postgraduate Diploma or full two‑year M.Sc. Credits: Total credits not stated (coursework totals provided, but full programme credits not explicitly stated). Programme Length: Two years for M.Sc.; one‑year exit option for PG Diploma. Academic vs CPD: Academic programme Stackable / Pathway: Stackable pathway with multiple exit points (PG Diploma → M.Sc.). | Foundational and advanced molecular biology; molecular diagnostics; statistical methods in diagnostics; ethics in diagnostics; cell culture and animal lab skills; stem cell applications; bioinformatics and structure‑based drug design; flow cytometry; genetics principles and analysis; genomic technologies; data analysis; pharmacogenomics and personalised medicine; molecular basis of disease; AI in diagnostics; molecular pathology of infectious disease; research dissertation. |
| 30 | Continent: Asia Country: India Type of Institution: University Level of Qualification: Graduate-level course (module within MSc) Programme Title/Subject: Precision and Translational Medicine Accreditation / Professional Recognition: Not stated Mode of Delivery: In person | Target Audience: Not stated Entry Requirements: Not stated | Award on Completion: Module credit: 3 credits Programme Length: Not stated Academic vs CPD: Academic Stackable / Pathway: Module within MSc | Core Learning Outcomes: Understands concepts and principles of precision medicine and translational research; applies molecular diagnostic techniques in precision medicine; examines personalised and targeted therapeutic approaches; valuates biomarker discovery and validation strategies; analyses implementation challenges in precision medicine; critically reviews case studies in precision medicine applications; develops problem‑solving and critical thinking in translational medicine contexts. |
| 31 | Continent: Asia Country: India Type of Institution: University Level of Qualification: Postgraduate Diploma Programme Title/Subject: Personalized Genomic Medicine Accreditation / Professional Recognition: Not stated Mode of Delivery: In person | Target Audience: Individuals with a background in biological sciences and a basic understanding of genetics Entry Requirements: Postgraduate degree in medical sciences, basic sciences in life science fields, engineering sciences in biotechnology, or pharmaceutical sciences, plus written entrance test | Award on Completion: Postgraduate Diploma Credits: 40 credits Programme Length: 12 months Academic vs CPD: Academic Stackable / Pathway: Not stated | Core Learning Outcomes: Applies theoretical and practical knowledge in genomic precision medicine; develop skills in next-generation sequencing and clinical genomics; conduct research on clinically relevant subjects in genomic medicine. |
| 32 | Continent: Asia Country: India Type of Institution: Organisation Level of Qualification: Certificate (3‑month distance) Programme Title/Subject: Medical Informatics  Accreditation / Professional Recognition: Not stated Mode of Delivery: Online | Target Audience: Practitioners in medicine, nursing, physiotherapy, paramedical fields, and individuals with interest in medical informatics Entry Requirements: Degree or diploma in listed health professions or undergraduate/graduate/postgraduate qualification with interest in medical informatics | Award on Completion: Certificate in Medical Informatics Credits: Not stated Programme Length: Three months Academic vs CPD: Short course Stackable / Pathway: Not stated | Core Learning Outcomes: Develops understanding of ICT in medicine; builds competence in digital tools in healthcare; enhances capability in computer applications; strengthens knowledge of tele-medicine concepts, applies medical informatics principles in practice. |
| 33 | Continent: Europe Country: UK Type of Institution: University Level of Qualification: Postgraduate Certificate Programme Title/Subject: Clinical Genetics and Genetic Counselling Accreditation / Professional Recognition: Not stated Mode of Delivery: Blended learning | Target Audience: Senior clinicians and academics Entry Requirements: Not stated | Award on Completion: Postgraduate Certificate Credits: 60 credits (4 modules × 15 credits each) Programme Length: Not stated Academic vs CPD: Academic Stackable / Pathway: Not stated | Core Learning Outcomes: Understands genomic science, genome structure, variation and disease mechanisms; applies genetic risk assessment methods and interprets genomic test results; communicates genomic information and supports patient and family decision‑making; assesses psychosocial and ethical implications of genomic testing; integrates genomic testing and diagnosis into clinical practice |
| 34 | Continent: Asia Country: Japan Type of Institution: University Level of Qualification: MSc Programme Title/Subject: Genetic/Genomic Nursing Accreditation / Professional Recognition: Accredited as a 38‑credit certified nurse specialist program by the Japan Association of Nursing Programs in Universities Mode of Delivery: In person | Target Audience: Nursing students seeking advanced practice preparation Entry Requirements: Not stated | Award on Completion: M.H.M; Certified Nurse Specialist qualification (Genetics) Credits: 38 credits for CNS component (full program credits not stated) Programme Length: Not stated Academic vs CPD: Academic Stackable / Pathway: Integrated five‑year bachelor‑to‑master pathway and CNS specialization | Core Learning Outcomes: Develops expertise in genetic risk assessment and genomic information use across the lifespan; applies genomic knowledge to diagnosis, prevention, and treatment decision support; addresses ethical, legal, and social issues in genomic medical care; implements nursing roles in genomic medicine and supports patients with hereditary conditions; contributes to system development for equitable genetic and genomic healthcare |
| 35 | Continent: Europe Country: UK Type of Institution: University Level of Qualification: MSc  Programme Title/Subject: Genomic Medicine Accreditation / Professional Recognition: Not stated Mode of Delivery: In person (via blended learning including online components) | Target Audience: Medical programme students and applicants from medical, biomedical, or healthcare backgrounds Entry Requirements: Minimum 2:1 degree in medical, biomedical, or healthcare subject; applicants with lower grades considered with significant relevant experience | Award on Completion: MSc Credits: Not stated Programme Length: One year full‑time or two years part‑time Academic vs CPD: Academic Stackable / Pathway: Not stated | Core Learning Outcomes: Understands genomic science foundations including human genetics and omics technologies; applies genomic analysis methods for common, rare, and inherited diseases; interprets genomic sequencing data and employs bioinformatics approaches; integrates cancer genomics into diagnosis, screening, and treatment; evaluates ethical, legal, and social considerations in applied genomics; develops research capability in medical genomics through project work |
| 36 | Continent: Asia Country: Lebanon Type of Institution: University Level of Qualification: Online Certificate Programme Title/Subject: Pharmacogenomics Accreditation / Professional Recognition: Not stated Mode of Delivery: Online | Target Audience: Fresh graduates, early‑career healthcare professionals, pharmacists, lab technologists, biomedical researchers, healthcare professionals broadly, medical centers, hospitals, laboratories, healthcare systems, pharmaceutical companies, academic institutions Entry Requirements: Bachelor's degree in a healthcare or biomedical field and meeting English proficiency requirements | Award on Completion: Online Certificate Credits: 3 credits Programme Length: 7 weeks Academic vs CPD: CPD Stackable / Pathway: Not stated | Students explain gene‑variant impacts on drug response, identify genetic sources of variability, locate pharmacogenomic information, interpret levels of evidence, evaluate clinical guidelines, assess ethnicity considerations, differentiate genotyping methods, and interpret pharmacogenomic test reports |
| 37 | Continent: Asia Country: Singapore Type of Institution: University Level of Qualification: Executive Certificate (Postgraduate CPD) Programme Title/Subject: Human Genomics in Precision Medicine Accreditation / Professional Recognition: Not stated Mode of Delivery: Blended | Target Audience: Individuals working in healthcare, research or diagnostic laboratories, biotechnology, pharmacology, academic medicine, or education Entry Requirements: Bachelor’s degree with possible requirement for relevant work experience; applicants with other qualifications may be considered case by case | Award on Completion: Executive Certificate in Human Genomics in Precision Medicine and Certificate of Completion for PHM5001 Credits: Not stated Programme Length: 13 weeks Academic vs CPD: Academic programme (postgraduate CPD) Stackable / Pathway: Stacks toward a Graduate Certificate in Fundamentals of Precision Medicine | Learners understand genetic and genomic bases of disease, apply omics and analytic tools for biomarker identification and diagnosis, use genomic strategies for prevention and treatment, and apply genomic tools and signatures for disease prediction and prognosis |
| 38 | Continent: Asia Country: Singapore Type of Institution: University Level of Qualification: Graduate Certificate Programme Title/Subject: Fundamentals of Precision Medicine Accreditation / Professional Recognition: Not stated Mode of Delivery: Blended | Target Audience: Individuals working in healthcare, research, diagnostic laboratories, biotechnology, pharmacology, academic medicine, or education Entry Requirements: Bachelor’s degree with possible relevant work experience; applicants with other qualifications considered on a case‑by‑case basis | Award on Completion: Graduate Certificate in Fundamentals of Precision Medicine Credits: Not stated Programme Length: Not stated Academic vs CPD: Academic programme Stackable / Pathway: Comprises two executive certificates forming a stackable pathway | Understanding genetic mechanisms of disease; applying OMICs and analytic tools for biomarker identification and disease prediction; using genomic strategies for treatment and mitigation; understanding proteomic and metabolomic technologies; recognising trends in molecular systems biology; understanding technological and clinical implementation challenges |
| 39 | Continent: Asia Country: Singapore Type of Institution: University Level of Qualification: Executive Certificate (Short Course Series) Programme Title/Subject: Clinical Genomics Accreditation / Professional Recognition: Not stated Mode of Delivery: In-person | Target Audience: Not stated Entry Requirements: Not stated | Award on Completion: Executive Certificate in Clinical Genomics Credits: 4 academic units Programme Length: Two weeks Academic vs CPD: Academic programme Stackable / Pathway: Not stated | Understanding fundamentals of genetics and genetic testing; understanding applications of clinical genetics; recognising the role of genetic counselling and related issues; applying genetic counselling in practical contexts including variant curation |
| 40 | Continent: Asia Country: Singapore & Asia-wide Type of Institution: University Level of Qualification: MSc (Modular) Programme Title/Subject: Precision Health and Medicine Accreditation / Professional Recognition: Not stated Mode of Delivery: In person | Target Audience: Students from medical and STEM backgrounds; healthcare professionals, policy makers, communicators, educators, researchers, scientists, and industry professionals supporting precision health implementation Entry Requirements: M.B.B.S. or relevant bachelor’s degree in science, technology, engineering, mathematics or medicine; quantitative aptitude desirable for non‑quantitative majors; examples include participation in quantitative competitions or completion of basic programming or statistics | Award on Completion: MSc in Precision Health and Medicine Credits: 40 units Programme Length: One year full‑time or two years part‑time Academic vs CPD: Academic programme Stackable / Pathway: Stackable pathway option available | Foundational genomic principles; proteomics and metabolomics; statistical and computational methods; high‑performance computing; AI and machine learning; ethics and regulatory frameworks; managerial economics; clinical integration of precision medicine; biomarker identification; diagnostics; therapeutics; drug discovery and pharmacogenomics; causal inference; technological innovation; cardiometabolic precision approaches; population‑level precision health |
| 41 | Continent: Asia Country: United Arab Emirates Type of Institution: University Level of Qualification: MSc Programme Title/Subject: Genomic Medicine Accreditation / Professional Recognition: Not stated Mode of Delivery: In person | Target Audience: Healthcare workforce from multiple disciplines including medicine, nursing, scientists, and biotechnologists Entry Requirements: Bachelor’s degree in medicine, biomedical sciences, or related disciplines; minimum GPA 3.0/4.0; IELTS 6.0 or equivalent; formal interview; test score less than two years old | Award on Completion: MSc in Genomic Medicine Credits: 33 credit hours Programme Length: Minimum three and maximum six semesters Academic vs CPD: Academic programme Stackable / Pathway: Not stated | Understanding advanced molecular biology and human genetics; analysing molecular mechanisms of human disease; applying bioinformatics and genomics; conducting genomic diagnostics; understanding OMICS in medicine; applying biostatistics; engaging in translational medicine; understanding pharmacogenomics and stratified medicine; understanding principles of genetic counselling |
| 42 | Continent: North America Country: USA Type of Institution: University Level of Qualification: Certificate (6‑month training program) Programme Title/Subject: Clinical Genomic Medicine and Genetic Counselling Accreditation / Professional Recognition: Not stated Mode of Delivery: In person | Target Audience: Clinicians and clinical researchers who manage patients with genetic conditions Entry Requirements: Not stated | Award on Completion: Certificate (training program) Credits: Not stated Programme Length: 6 months Academic vs CPD: Academic program Stackable / Pathway: Not stated | Understanding foundational genomics; understanding monogenic and common genetic diseases; applying genetic counselling and screening; applying genomic therapeutics; understanding ethics and regulatory aspects; understanding genomic economics; applying AI‑enhanced genomic medicine |
| 43 | Continent: North America Country: Canada Type of Institution: University Level of Qualification: Graduate Certificate / Graduate Diploma / MSc Programme Title/Subject: Precision Health with Precision Medicine Specialization Accreditation / Professional Recognition: Not stated Mode of Delivery: Online | Target Audience: Broad healthcare professionals including medicine, veterinary medicine, nursing, pharmacy, psychology, optometry, dentistry, and other health‑related fields Entry Requirements: Four‑year baccalaureate degree in a health‑related discipline; Graduate Certificate requires GPA 3.3; Diploma requires completion of the Certificate with GPA 3.30; Master’s requires completion of the Diploma with GPA 3.30; English language proficiency required for non‑native speakers | Award on Completion: Graduate Certificate in Precision Health; Graduate Diploma in Precision Health; Master of Precision Health Credits: Certificate 12 credits; Diploma 24 units; Master’s 33 units Programme Length: Certificate 10–12 months; Diploma 12 months; Master’s 2 years full‑time or 3 years part‑time Academic vs CPD: Academic programmes Stackable / Pathway: Laddered pathway enabling stepwise progression. | Applying genome sequencing and molecular tools; utilising omics in clinical practice; applying pharmacogenomics and precision oncology; integrating AI in precision health; applying ethics and law; managing complex projects and health‑system leadership; applying advanced precision medicine concepts; applying innovation, safety, data interpretation, and experiential learning in implementation and evaluation |
| 44 | Continent: North America Country: USA Type of Institution: University Level of Qualification: Postgraduate Certificate / MSc Programme Title/Subject: Precision Medicine Accreditation / Professional Recognition: Not stated Mode of Delivery: Online | Target Audience: Individuals working in healthcare or industry settings, researchers, teachers, post‑doctoral fellows, graduate students, and healthcare workers including nurses, nurse practitioners, physician assistants, pharmacists, medical doctors, and doctors of osteopathy Entry Requirements: Graduate Certificate has no stated academic requirements; Master’s requires a graduate or professional healthcare degree from a regionally accredited institution, upper‑division GPA of 3.0 or higher, statement of purpose, and current CV | Award on Completion: Graduate Certificate in Precision Medicine; MSc in Pharmacy (Precision Medicine specialization) Credits: Graduate Certificate 9 credits; MSc 31 credits Programme Length: Graduate Certificate completed in 12 months; MSc completed in 2–3 years Academic vs CPD: Academic programmes Stackable / Pathway: Graduate Certificate serves as standalone credential; MSc represents advanced pathway but stacking relationship not explicitly stated | Foundations in genomic technologies, molecular genetics, genetic epidemiology, pharmacogenomics, clinical pharmacogenomics case analyses, implementation of pharmacogenomics, clinical applications of precision health, oncology applications, individualized pharmacotherapy, AI and informatics applications, patient education, medication management, and capstone assessment of precision medicine competencies |
| 45 | Continent: North America Country: USA Type of Institution: University Level of Qualification: Graduate Certificate Programme Title/Subject: Health Care Genetics Accreditation / Professional Recognition: Not stated Mode of Delivery: On site | Target Audience: Health care workers seeking focused graduate‑level education in genetics Entry Requirements: Bachelor’s or MSc from ACEN, NLN, or CCNE accredited programme; minimum GPA 3.0; pre‑admission interview; online application; additional requirements for international applicants | Award on Completion: Graduate Certificate in Health Care Genetics Credits: 15 credits Programme Length: Approximately three academic terms (full‑time or part‑time) Academic vs CPD: Academic programme Stackable / Pathway: Not stated | Understanding genetic basis of disease; guiding patient understanding and education on genetic testing, interpretation, recurrence risks and therapies; advocating for patients with genetic conditions; using family histories for risk identification; accessing genetic health resources; identifying need for specialist referral; interpreting genetic research findings |
| 46 | Continent: North America Country: USA Type of Institution: University Level of Qualification: Graduate Certificate Programme Title/Subject: Pharmacogenomics & Personalized Healthcare Accreditation / Professional Recognition: Not stated Mode of Delivery: Online | Target Audience: Individuals seeking to work in pharmacogenomics or qualify for roles involving personalized healthcare Entry Requirements: Bachelor’s degree in biology, chemistry, or science‑related field; minimum 3.0 GPA; English language proficiency for international students. | Award on Completion: Graduate Certificate in Pharmacogenomics & Personalized Healthcare Credits: 15 credits Programme Length: Not stated Academic vs CPD: Academic programme Stackable / Pathway: Not stated | Metabolism and molecular biology; pharmacogenomics and clinical application of pharmacogenetics; biostatistics and research methods; pharmaceutical biotechnology; drug development; careers in pharmacogenomics; implementation science; nutrition and exercise genomics; cancer genomics; advanced pharmacogenomics; human genetics |
| 47 | Continent: North America Country: USA Type of Institution: University Level of Qualification: Graduate Certificate Programme Title/Subject: Personalized & Genomic Medicine Accreditation / Professional Recognition: Not stated Mode of Delivery: Online | Target Audience: Current and future healthcare professionals, research professionals, and recent graduates seeking exposure or expertise in personalized medicine and genomics Entry Requirements: Bachelor’s degree with minimum 3.0 GPA and proficiency in English language skills | Award on Completion: Graduate Certificate in Personalized & Genomic Medicine Credits: 12 credits Programme Length: Approximately 12 months (two semesters) Academic vs CPD: Academic programme Stackable / Pathway: Not stated | Understanding foundations of personalized health; applying pharmacogenomics; using multi‑omic approaches; analysing observational health data; integrating genomics with other omics and electronic health data; applying personalized medicine concepts in research, clinical, and industry contexts |
| 48 | Continent: North America Country: USA Type of Institution: University Level of Qualification: Graduate Certificate Programme Title/Subject: Genomics Accreditation / Professional Recognition: Not stated Mode of Delivery: Online | Target Audience: Individuals in business, science and healthcare fields and those preparing for advanced study in genomics Entry Requirements: Bachelor’s degree from a regionally accredited institution with a demonstrated record of academic success | Award on Completion: Graduate Certificate Credits: 12 credits Programme Length: Not stated (individual courses are seven weeks; total programme duration not explicitly stated) Academic vs CPD: Academic programme Stackable / Pathway: Credits count towards an online MSc in genomics | Critical analysis and application of genetics and genomics; understanding of epigenetics and pharmacogenomics; exploration of social, ethical and legal issues in genetics and genomics |
| 49 | Continent: North America Country: USA Type of Institution: University Level of Qualification: Graduate Certificate Programme Title/Subject: Precision Medicine Accreditation / Professional Recognition: Not stated Mode of Delivery: Online | Target Audience: Clinicians and healthcare professionals Entry Requirements: Undergraduate degree and completion of at least one 3‑credit genetics course or related field | Award on Completion: Graduate Certificate Credits: 12 credits Programme Length: Not stated Academic vs CPD: Academic programme Stackable / Pathway: Not stated | Understanding of precision medicine foundations; knowledge of bioethics in precision medicine; understanding of cancer precision medicine; understanding of medical genetics, rare diseases, pharmacogenomics and epigenomics |
| 50 | Continent: North America Country: USA Type of Institution: University Level of Qualification: MSc (M.S.) Programme Title/Subject: Precision Medicine Accreditation / Professional Recognition: Approved by the State of Arizona Board of Private Postsecondary Education; institutional accreditation by the Higher Learning Commission (HLC/NCA) Mode of Delivery: Online | Target Audience: Students in healthcare professional programmes seeking an applied genomic sciences dual degree, including DO, Dental Medicine, Optometry and Veterinary Medicine Entry Requirements: Acceptance to a primary degree program, minimum cumulative GPA 2.75, official transcripts, completed programme application, automatic dean approval request for current students, passage of criminal background check | Award on Completion: MSc Credits: 46 quarter‑credit hours (including up to 18 quarter‑credit hours transferred from the primary programme) Programme Length: 2 years Academic vs CPD: Academic programme Stackable / Pathway: Dual‑degree structure with transfer of credits from the primary programme; transfer from Post‑Graduate Certificate to MSc possible with programme director approval | Genetics and genomics foundations; bioinformatics, statistics and data interpretation; pharmacogenomics; ‘omics technologies (transcriptomics, proteomics, metabolomics); disease‑focused precision medicine (cancer, rare and complex, infectious, inflammatory, cardiovascular, neurological); ethical, legal and social issues; counselling and communication skills; personal genomic analysis and capstone report; interpretation of direct‑to‑consumer genetic testing |
| 51 | Continent: South America Country: Argentina Type of Institution: University Level of Qualification: Postgraduate Diploma Programme Title/Subject: Applied Genomics Accreditation / Professional Recognition: Not stated Mode of Delivery: Blended | Target Audience: General practitioners, paediatricians, neonatologists, obstetricians, neurologists, cardiologists and other healthcare professionals seeking to deepen their knowledge of genomics Entry Requirements: Not stated | Award on Completion: Postgraduate Diploma Credits: Not stated Programme Length: 10 months Academic vs CPD: Academic programme Stackable / Pathway: Not stated | Knowledge of basic and clinical genetics; understanding of genomic diagnostic techniques; interpretation of genetic variants; application of genomics in oncology, cardiology, neurology, paediatrics and perinatal care; ethical and legal aspects of genetics; communication and counselling in genomic medicine; understanding of gene therapy and reproductive genetics |
| 52 | Continent: South America Country: Brazil Type of Institution: University Level of Qualification: Postgraduate Certificate Programme Title/Subject: Bioinformatics Applied to Medical Genomics Accreditation / Professional Recognition: Not stated Mode of Delivery: Blended | Target Audience: Professionals with basic education in health sciences or computer sciences Entry Requirements: Basic education in health sciences or computer sciences; graduation date must be prior to programme start; no prior programming experience required; interest or aptitude for computational tools recommended; preparatory courses recommended for those without programming or molecular biology background | Award on Completion: Postgraduate Certificate (lato sensu) Credits: Not specified Programme Length: 12 months Academic vs CPD: Academic programme Stackable / Pathway: Not stated | Understanding of human molecular and medical genetics; understanding of evolutionary genetics; use of statistical concepts in genomics; understanding of NGS methodology; manipulation of raw NGS data; microbial genomics and metagenomics analysis; phylogenetic reconstruction; gene expression analysis; application of machine learning models to genomic datasets |
| 53 | Continent: South America Country: Brazil Type of Institution: University Level of Qualification: Postgraduate Certificate Programme Title/Subject: Bioinformatics Applied to Medical Genomics – Analysis of Germline and Somatic Variants Accreditation / Professional Recognition: Not stated Mode of Delivery: Blended | Target Audience: Professionals with basic education in health sciences or computer sciences Entry Requirements: Basic education in health sciences or computer sciences; prior programming experience not required; interest or aptitude for computational tools recommended; preparatory programming or molecular biology courses recommended; graduation date must be prior to programme start | Award on Completion: Postgraduate Certificate (lato sensu) Credits: Not stated Programme Length: 11 months Academic vs CPD: Academic programme Stackable / Pathway: Not stated | Knowledge of human molecular and medical genetics; understanding of evolutionary genetics; application of statistical concepts in genomics; understanding of NGS molecular basis; alignment and quality assessment of NGS data; qualitative and quantitative sequencing data interpretation; identification and interpretation of germline and somatic variants |
| 54 | Continent: South America Country: Brazil Type of Institution: University Level of Qualification: Postgraduate Certificate Programme Title/Subject: Cell Therapy Accreditation / Professional Recognition: Not stated Mode of Delivery: Blended | Target Audience: Professionals with higher education in health‑related fields including biologists, biomedical scientists, biochemists, dentists, nurses, biomedical engineers, pharmacists, physiotherapists, physicians and other health professionals involved in research, development or execution of advanced cell therapy protocols Entry Requirements: Higher education qualification with graduation date prior to programme start | Award on Completion: Postgraduate Certificate (lato sensu) Credits: Not stated Programme Length: 12 months Academic vs CPD: Academic programme Stackable / Pathway: Can be taken sequentially with the Postgraduate Program in Gene Therapy | Knowledge of cellular and molecular biology applied to cell therapy; understanding of translational research; understanding of manufacturing processes for advanced cell therapies; awareness of clinical trials and regulatory approvals; understanding of quality controls; understanding of ethical, social and economic issues in cell therapy |
| 55 | Continent: South America Country: Brazil Type of Institution: University Level of Qualification: Postgraduate Certificate Programme Title/Subject: Gene Therapy Accreditation / Professional Recognition: Not stated Mode of Delivery: Blended | Target Audience: Professionals with higher education degrees in medicine, biology, biomedicine, bioengineering, pharmacy, nursing, physiotherapy, dentistry, veterinary medicine and related fields working or intending to work in gene editing and/or gene therapy Entry Requirements: Higher education degree with graduation date prior to programme start | Award on Completion: Postgraduate Certificate (lato sensu) Credits: Not stated Programme Length: 12 months Academic vs CPD: Academic programme Stackable / Pathway: Can be taken sequentially with Postgraduate Program in Cell Therapy | Knowledge of cellular and molecular biology applied to gene therapy; understanding of gene editing development and methodologies; knowledge of delivery mechanisms and technological innovations; understanding of manufacturing challenges for gene therapy products; awareness of clinical trials in gene therapy; understanding of gene therapy applications across medical specialties; awareness of ethical, social and economic issues in gene therapy |
| 56 | Continent: South America Country: Chile Type of Institution: University Level of Qualification: Postgraduate Diploma Programme Title/Subject: Precision Healthcare Accreditation / Professional Recognition: Not stated Mode of Delivery: Blended | Target Audience: Healthcare professionals Entry Requirements: Being a healthcare professional; submission of identity card and professional title | Award on Completion: Diploma Credits: Not specified Programme Length: 250 hours (July 27–December 14, 2024) Academic vs CPD: Academic programme Stackable / Pathway: Not stated | Applications of precision health; pharmacogenomics and genetic variation in drug response; nutritional genomics and metabolomics; biomarkers and early diagnosis in cancer; interdisciplinary precision health approaches; strategies for prevention, diagnosis and monitoring based on personalized precision medicine |
| 57 | Continent: South America Country: Chile Type of Institution: University Level of Qualification: Postgraduate Diploma Programme Title/Subject: Genetic Counselling in Hereditary Cancer Syndromes Accreditation / Professional Recognition: Not stated Mode of Delivery: Blended (asynchronous online classes with mandatory final in‑person session) | Target Audience: Health professionals linked to hospital oncology units including doctors, nurses, midwives, psycho‑oncologists, biochemists and medical technologists Entry Requirements: Professional title or degree; identity document; curriculum vitae; basic English comprehension for reading clinical‑scientific texts | Award on Completion: Diploma Credits: 16 SCT credits Programme Length: Not clearly specified (delivered across 2 semesters) Academic vs CPD: Academic programme Stackable / Pathway: Not stated | Identification and evaluation of hereditary cancer syndromes; interpretation of genetic tests; support and follow‑up of patients and families; application of clinical recommendations; interdisciplinary collaboration; development of hereditary tumour programmes |
| 58 | Continent: Oceania Country: Australia Type of Institution: University Level of Qualification: MSc Programme Title/Subject: Genomics & Precision Medicine Accreditation / Professional Recognition: One genomics unit approved for a clinical genetics training requirement; programme-level accreditation not stated Mode of Delivery: Online | Target Audience: Clinicians seeking advanced knowledge in genomics and precision medicine, including those interested in clinical genetics, paediatrics, obstetrics, neurology, and oncology Entry Requirements: Open only to applicants already enrolled in the MSc; must have completed required coursework with specified credit thresholds; standard documentation required; broader entry requirements for streamed pathways noted but not specific to this advanced programme | Award on Completion: MSc Credits: 60 credit points Programme Length: 1 year full‑time Academic vs CPD: Academic programme Stackable / Pathway: Available as PGDip and PGCert, and graduates may proceed to an MSc (Advanced. | Foundations of clinical epidemiology and critical appraisal; application of genomic science to clinical practice, ethical frameworks, evidence interpretation and decision-making in genomics; genomic science, variant interpretation and clinical application across specialties; cancer genomics, hereditary cancer syndromes and precision oncology approaches; bioinformatics, omics methodologies and functional genomics; multidisciplinary genomic practice and integration of genomic diagnostics; advanced scholarly capability through structured project-based work. |
| 59 | Continent: Oceania Country: Australia Type of Institution: University Level of Qualification: MSc Programme Title/Subject: Genomics and Health Accreditation / Professional Recognition: Not stated Mode of Delivery: Blended (online plus face‑to‑face tutorials and workshops) | Target Audience: Individuals in clinical, health, education, public health, research or laboratory science fields; recent graduates in genetics or related disciplines; medical and allied health professionals; individuals with health or science backgrounds seeking roles in clinical trials, community organisations, government, research or education Entry Requirements: Undergraduate degree in a directly related discipline with required grades and biology/genetics prerequisites; or six years of relevant work experience plus prerequisites; or undergraduate degree in any discipline plus prerequisites and work experience; 400‑word personal statement, subject descriptions, and references required; shortlisted applicants interviewed; English‑language standards apply | Award on Completion: MSc Credits: 200 points Programme Length: 2 years full‑time Academic vs CPD: Academic programme Stackable / Pathway: Nested award with exit points at Graduate Certificate and Graduate Diploma levels | Human genetics, genomic mechanisms and disease complexity; genomic risk assessment tools, genome variant analysis, variant classification, genomic data interpretation; ethical, legal and social implications of genomics in clinical and public health contexts; foundational and advanced communication skills for genomic practice and counselling in healthcare contexts; clinical genomics including complex disease traits and cancer genetics; translational genomics, workforce considerations and implementation into practice; research design, methodology and supervised genomic research projects; bioinformatics, computational genomics and genomic data workflows; public health genomics, community engagement and digital health transformation. |
| 60 | Continent: Oceania Country: New Zealand Type of Institution: University Level of Qualification: Postgraduate Certificate Programme Title/Subject: Genomic Health & Medicine Accreditation / Professional Recognition: Not stated Mode of Delivery: Online | Target Audience: Healthcare professionals, government policy workers, scientists, statisticians, data modellers, health researchers Entry Requirements: Bachelor’s degree in a health‑related field or a health professional qualification requiring at least three years of tertiary study; experience or current employment in a relevant health field required for endorsed diploma options; admission subject to approval of the Pro‑Vice‑Chancellor; programme must be completed within two years | Award on Completion: Postgraduate Certificate in Health Sciences Credits: 60 points Programme Length: 1 semester full‑time (up to 2 years part‑time) Academic vs CPD: Academic programme Stackable / Pathway: Papers may be used as part of other qualifications | Introduction to genomics, pharmacogenomics, epigenetics and genetic testing; Case‑based genomic medicine principles and global and national genomic applications; practical implementation of genomic medicine, big‑data analysis and mini research project skills; clinical bioinformatics foundations, multi‑omic data analysis, analytical tool use and experimental design; ethical, legal, social and cultural considerations in genomic health practice; communication of genomic concepts, research skills and reflective practice |
| 61 | Continent: Europe Country: UK Type of Institution: University Level of Qualification: MSc Programme Title/Subject: Genomic Medicine Accreditation / Professional Recognition: Not stated Mode of Delivery: Blended | Target Audience: Recent graduates and healthcare professionals seeking specialisation in genomics; individuals working in clinical diagnostics, clinical trials, bioinformatics, research or health‑related roles Entry Requirements: First‑class or strong upper second‑class degree in relevant fields; relevant subject areas include biological or biomedical sciences, medicine, nursing, bioinformatics, computer science, statistics or quantitative disciplines; substantial professional experience may be accepted; English proficiency required at higher level; three academic references; interview required | Award on Completion: MSc Credits: Not specified Programme Length: One year full‑time Academic vs CPD: Academic programme Stackable / Pathway: Includes research project; optional modules allow customisation; no formal stackable pathway stated | Foundational genomics, genetics and inherited disease mechanisms; omic technologies, genomic data analysis and bioinformatics pipelines; academic and research skills including scientific communication and computational training; precision medicine, molecular pathology and gene‑based therapeutics; clinical genomics applications, translational genomics and health system integration; genome engineering, single‑cell and spatial omics, and therapeutic applications |
| 62 | Continent: Europe Country: Ireland Type of Institution: University Level of Qualification: MSc Programme Title/Subject: Technologies and Analytics in Precision Medicine Accreditation / Professional Recognition: Not stated Mode of Delivery: Blended | Target Audience: Individuals with undergraduate healthcare degrees in pharmacy or medicine; individuals with primary degrees in biological, chemical or mathematical/statistical sciences including computer science Entry Requirements: Undergraduate healthcare degree or 2.1 honours degree in biological, chemical or mathematical/statistical science; applicants with 2.2 honours plus industrial experience considered; short online interview required; visa support letter available | Award on Completion: MSc Credits: 90 ECTS Programme Length: 1 year Academic vs CPD: Academic programme Stackable / Pathway: Not stated | Genetics, genomics and precision medicine knowledge; data analytics, computational biology and programming in R and Python; connected health technologies, artificial intelligence and machine learning; innovation and leadership skills; application of analytic and genomic skills in a research project across industry or academia |
| 63 | Continent: Europe Country: Ireland Type of Institution: University Level of Qualification: MSc Programme Title/Subject: Genomic Medicine Accreditation / Professional Recognition: Not stated Mode of Delivery: Blended | Target Audience: Basic scientists, clinicians, and individuals working in life sciences, pharmaceutical industry, and related fields Entry Requirements: Not specified | Award on Completion: MSc Credits: 90 ECTS Programme Length: Full‑time 1 year or part‑time 2 years Academic vs CPD: Academic programme Stackable / Pathway: Optional modules enable tailored learning; research dissertation included | Human genomic variation, monogenic and polygenic disease processes; molecular and cellular mechanisms translating genomics to health outcomes; precision medicine and individualised treatments; genome-based disease risk prediction and drug discovery applications; host–environment and microbiome interactions; ethical and legal frameworks for genomics and health science research; computational genomic data analysis, NGS data processing and statistical methods; population genomics, epidemiological study design and genomic technologies; scientific communication, research skills, experimental design and research integrity |
| 64 | Continent: Europe Country: France Type of Institution: University Level of Qualification: Diploma Programme Title/Subject: Precision Diagnostics and Personalized Medicine Accreditation / Professional Recognition: Not stated Mode of Delivery: Blended | Target Audience: Doctors, pharmacists, dentists, midwives, medical interns, pharmacy interns, genetic counsellors, nurses, psychologists, ethics students, prescription assistants Entry Requirements: Enrolment in internal training or holding a diploma in health‑related training; selection by academic committee; application file including diploma, CV and cover letter | Award on Completion: University Diploma Credits: Not stated Programme Length: 105 hours Academic vs CPD: Academic programme Stackable / Pathway: Not stated | Mendelian and non‑Mendelian genetics principles; next ‑generation genetic tools and variant analysis; interpretation of variants using databases; genetics in constitutional pathologies, multifactorial oncological diseases and pharmacology; diagnostic and therapeutic applications of genomic data; ethical and societal implications of emerging genomic technologies |
| 65 | Continent: Europe Country: France Type of Institution: University Level of Qualification: University Diploma (CPD) Programme Title/Subject: Genomic Medicine – NGS for Genetic Diagnosis and Therapeutic Stratification Accreditation / Professional Recognition: Not stated Mode of Delivery: Blended | Target Audience: Healthcare professionals, medical biologists and scientists seeking upskilling in genomics for diagnostic, treatment and monitoring applications Entry Requirements: Admission by academic assessment; submission of CV, motivation letter and qualifying diplomas | Award on Completion: University Diploma Credits: Not stated Programme Length: 105 hours over 6 months Academic vs CPD: CPD/Academic programme Stackable / Pathway: Not stated | High ‑throughput sequencing approaches and technological principles; bioinformatics tools and computational methods for genomic data analysis; genomic medicine applications in rare diseases and cancer; variant analysis, classification and interpretation; NGS methodologies for Mendelian, mitochondrial, X‑linked and somatic conditions; cfDNA applications for oncology and prenatal screening; ethical and regulatory aspects of genetic testing; research dissertation presentation and defence |
